# Taxonomic review and phylogenetic analysis of fifteen North American *Entomobrya* (Collembola, Entomobryidae), including four new species

**DOI:** 10.3897/zookeys.525.6020

**Published:** 2015-10-05

**Authors:** Aron D. Katz, Rosanna Giordano, Felipe Soto-Adames

**Affiliations:** 1 Department of Entomology, University of Illinois, 320 Morrill Hall, 505 S. Goodwin Avenue, Urbana, IL 61801, USA; 2 Department of Biology, University of Puerto Rico, San Juan, PR 00931, USA

**Keywords:** Chaetotaxy, cryptic species, phylogeny, species diagnosis, *Willowsia*

## Abstract

The chaetotaxy of 15 species of eastern North American *Entomobrya* is redescribed in order to determine potential characters for the diagnosis of cryptic lineages and evaluate the diagnostic and phylogenetic utility of chaetotaxy. As a result, four new species (*Entomobrya citrensis* Katz & Soto-Adames, **sp. n.**, *Entomobrya jubata* Katz & Soto-Adames, **sp. n.**, *Entomobrya neotenica* Katz & Soto-Adames, **sp. n.** and *Entomobrya unifasciata* Katz & Soto-Adames, **sp. n.**) are described, and new diagnoses are provided for *Entomobrya assuta* Folsom, *Entomobrya atrocincta* Schött, *Entomobrya decemfasciata* (Packard), *Entomobrya ligata* Folsom, *Entomobrya multifasciata* (Tullberg), and *Entomobrya quadrilineata* (Bueker). Furthermore, previously undocumented levels of intraspecific variation in macrosetal pattern are reported, tempering the exclusive use of chaetotaxy for species delimitation. Phylogenetic relationships, estimated using both morphological and molecular data, indicate that *Entomobrya* is likely paraphyletic. The phylogenies also suggest that unreliable character homology, likely fostered by *Entomobrya*’s profusion of macrosetae, may limit the phylogenetic utility of chaetotaxy in groups characterized by an abundance of dorsal macrosetae.

## Introduction

Studies concerning species delimitation and taxonomy of Collembola have traditionally relied on comparative morphology. For some groups, however, uncertain homology, intraspecific variation, and characters of difficult observation have limited the utility of morphological characters as tools for species diagnosis and phylogenetic inference. Recent advances in DNA sequencing technology have provided scientists with additional ways to delimit groups that lack informative morphology, such as cryptic species complexes (Frati et al. 1994, 1995, 2000; Carapelli et al. 1995, 2005; Simonsen et al. 1999; Soto-Adames 2002; Burkhardt and Filser 2005; Cicconardi et al. 2010, 2013; Felderhoff et al. 2010; Porco et al. 2012; Zhang et al. 2014c; Katz et al. 2015), but in practice most species diagnosis remains dependent on fixed characters, only obtained by rigorous morphological examination (Will and Rubinoff 2004).

Taxonomy and species delimitation of the genus *Entomobrya* Rondani, 1861 has been especially problematic due to intraspecific morphological variation and a general lack of informative taxonomic characters (Christiansen 1958b; South 1961). Conspicuous dorsal color patterns exhibited by members of this genus originally served as a practical means for species diagnosis (Bonet 1934). Other characters such as claw structure, antennal sense organs, setae types, and chaetotaxy of the male genital plate were introduced in Christiansen’s (1958b) revision of North American *Entomobrya*, who attributed many color forms to intraspecific variants. These new characters marginalized color pattern as an exclusive diagnostic tool, but some are difficult to observe. Szeptycki’s (1979) publication on the phylogenetic significance of dorsal chaetotaxy, introduced standard nomenclature for dorsal setae, providing a practical system to assess element homology between species. This system established chaetotaxy as the most important tool for species diagnosis in the family Entomobryidae and in theory, provided many additional characters to infer phylogenetic relationships (Soto-Adames 2008). Christiansen and Bellinger (1980, 1998) later combined traditional morphology with chaetotaxy in order to clarify species boundaries. Jordana and Baquero (2005) then simplified Szeptycki’s (1979) system of body chaetotaxy and incorporated additional nomenclature for elements on the head later formalized by Soto-Adames (2008). Following this new nomenclature system, Jordana (2012) was able to delimit approximately 270 Palearctic species in subfamily Capbryinae and tribe Entomobryini using chaetotaxy.

Chaetotaxy has surely proven to be a valuable tool for springtail taxonomists, but not without complications. For some groups, especially those characterized by large numbers of setae, such as *Entomobrya*, the homology of macrosetae is not always clear. Intraspecific variation, apparent differences in setae arrangements, and differences of setae types make it difficult to determine homology between species (Soto-Adames 2008). Despite these complications, chaetotaxy still provides fixed observable differences that have been widely applied to successfully delimit species boundaries (Christiansen and Bellinger 1998; Carapelli et al. 2001; Jordana and Baquero 2005; Soto-Adames 2010; Jordana 2012). However, its utility as diagnostic and phylogenetic characters is clearly dependent on a detailed assessment of intraspecific variation and homology between species of interest.

Christiansen and Bellinger’s (1998) “The Collembola of North America” is the current authority for Nearctic Collembola species identification. However, some of their descriptions of *Entomobrya* express the need for re-examination due to a lack of informative characters for clear delineation of species boundaries, and high levels of observed intraspecific chaetotaxy and color pattern variation that suggest the presence of cryptic species complexes. In addition, their descriptions of chaetotaxy are limited and often unclear. Therefore, the primary goal of this study is to examine and document *Entomobrya* chaetotaxy to provide detailed descriptions and figures in order to clarify species boundaries and to simplify the diagnosis of eastern North American *Entomobrya* species. A total of 15 species of North American *Entomobrya* that occur east of the Mississippi River are examined and described with special emphasis on chaetotaxy and color pattern, including three cryptic species lineages identified by Katz et al. (2015) and two new species. Additionally, phylogenies, incorporating both the morphology described in this study and molecular COI sequences from Katz et al. (2015), are presented in order to explore how chaetotaxy and other morphological characters affect phylogenetic estimation.

## Methods

### Specimen collection and preparation

Approximately 146 specimens, representing 15 *Entomobrya* species (11 previously reported, 4 new), were examined in detail throughout the course of this study. Historical collections of *Entomobrya* from the Illinois Natural History Survey were also examined, but were not useful for the present study. Specimens preserved in 70% EtOH are old (e.g., collected prior to 1980) and in poor condition; although color pattern is often preserved, chaetotaxy and other small, but diagnostic, characters are extremely difficult to observe with confidence. Therefore, more recent material was needed to study details of chaetotaxy and other characters. Most specimens examined were collected by the senior author between 2011 and 2012 or otherwise provided by colleagues from localities throughout the USA, east of the Mississippi River. In the Material Examined sections it is assumed that the senior author collected all material, unless otherwise noted. Specimens were usually collected from leaf litter and extracted using a Berlese funnel or hand collected from bark and vegetation with an aspirator. Table 1 lists all *Entomobrya* species reported from North American.

**Table 1.** List of all *Entomobrya* species reported from North America. Of the 31 species, 15 were examined for this study and 16 were not included.

Species examined and described for this study	Species not included in study	
	eastern species^1^	western species^2^
*Entomobrya assuta*	*Entomobrya comparata*	*Entomobrya arnaudi*
*Entomobrya atrocincta*	*Entomobrya confusa*	*Entomobrya arula*
*Entomobrya bicolor*	*Entomobrya gisini*	*Entomobrya erratica*
*Entomobrya citrensis* sp. n.	*Entomobrya griseoolivata*	*Entomobrya kincaidi*
*Entomobrya clitellaria*	*Entomobrya sinelloides*	*Entomobrya nigriceps*
*Entomobrya decemfasciata*		*Entomobrya suzannae*
*Entomobrya intermedia*		*Entomobrya triangularis*
*Entomobrya jubata* sp. n.		*Entomobrya troglodytes*
*Entomobrya ligata*		*Entomobrya troglophila*
*Entomobrya multifasciata*		*Entomobrya washingtonia*
*Entomobrya neotenica* sp. n.		*Entomobrya zona*
*Entomobrya nivalis*		
*Entomobrya quadrilineata*		
*Entomobrya unifasciata* sp. n.		
*Entomobrya unostrigata*		

1 Species reported east of the Mississippi River that were not obtained for this study.

2 Species with western North American distributions that have not been reported east of the Mississippi River. These species were not obtained for this study.

Individuals sampled were sorted under a dissecting microscope to morphospecies according to color pattern and photographed to record dorsal thoracic and abdominal color patterns prior to slide mounting. All specimens were cleared with Nesbitt’s solution and mounted on Hoyer’s medium (Mari Mutt 1979) in preparation for light microscopy. The heads of specimens generally take longer to clear so they were dissected and mounted separately. Heads and bodies were both mounted dorsal-side up; the optimal position for observing important morphological characters. All illustrations were hand-drawn under a camera lucida then scanned and digitized with Adobe Illustrator CS6 software. Previously described species included in this study were identified based on morphological characters as delimited in Collembola of North America (Christiansen and Bellinger 1998).

All type specimens of *Entomobrya assuta*, *Entomobrya decemfasciata*, *Entomobrya ligata*, and *Entomobrya quadrilineata* deposited at the Illinois Natural History Survey, Champaign, IL, were examined. Types were either unavailable or their repository was unknown for most other species included in this study: *Entomobrya atrocincta*, unknown; *Entomobrya bicolor* Guthrie, unknown; *Entomobrya clitellaria* Guthrie, type stored at the Department of Animal Biology at the University of Minnesota (unavailable); *Entomobrya intermedia* Brook, unknown; *Entomobrya multifasciata*, unknown; *Entomobrya nivalis* (Linnaeus), unknown; and *Entomobrya unostrigata* Stach, type stored at the Institute of Systematics and Evolution of Animals of the Polish Academy of Sciences, Krakow, Poland (not examined). Additional types for *Entomobrya decemfasciata* are stored at the Museum of Comparative Zoology, Harvard (unavailable), and *Entomobrya ligata* and *Entomobrya assuta* at the American Museum of Natural History, New York (unavailable).

Four new species are described and named based on morphological and molecular differentiation: *Entomobrya unifasciata* sp. n., *Entomobrya citrensis* sp. n., *Entomobrya neotenica* sp. n. and *Entomobrya jubata* sp. n., originally identified as *Entomobrya ligata* color form B, *Entomobrya assuta* color form D, *Entomobrya* sp. n. 1 and *Entomobrya* sp. n. 2 in Katz et al. (2015). The cryptic lineages of *Entomobrya quadrilineata* color forms B, C, and D in Katz et al. (2015) are identified as *Entomobrya decemfasciata*.

## Discussion of characters

*Color pattern*: *Entomobrya* is characterized by distinct, but often complex and variable, color patterns. Given this diversity of color forms, color pattern was given a secondary diagnostic role partly due to confusion in the taxonomic literature caused by presumed occurrences of intra- and inter-population color pattern variation (Christiansen 1958b; South 1961). However, recent molecular phylogenetic studies have supported the resurgence of color pattern as a valid tool for species delimitation in some springtail groups (Carapelli et al. 1995, 2005; Frati et al. 1994, 1995, 2000; Simonsen et al. 1999; Soto-Adames 2002) including *Entomobrya* (Katz et al. 2015). For some species, color pattern serves as an easily observable and valid diagnostic tool when combined with traditional characters. Therefore, this study is mainly concerned with dorsal chaetotaxy and color pattern for species descriptions and diagnosis.

*Apical bulb of 4^th^ antennal segment*: The apical bulb of the 4^th^ antennal segment is present in all species of North American *Entomobrya* (except *Entomobrya sinelloides* Christiansen, 1958b) and has a number of different forms (e.g., single lobe, bi-lobed, tri-lobed). Christiansen and Bellinger (1980, 1998) incorporate these different character states into their species descriptions and diagnostic tables. However, apical bulbs are prone to misinterpretation of character states, being absent or irregular due to antennal regrowth, and moderate intraspecific variation. Therefore, this character is described here, but not considered useful for species diagnosis.

*Apical sense organ of 3^rd^ antennal segment*: This setal complex consists of two sense pegs, “guard setae”, and a number of differentiated setae (Chen and Christiansen 1993). These characters are very difficult to observe and lack useful variation for *Entomobrya* species diagnosis.

*Differentiated setae on ventral side of 1^st^ antennal segment*: Several small, spine-like setae occur on the ventral side of the 1^st^ antennal segment. However, no useful variation was observed for the diagnosis of species treated here.

**Figure 1.**
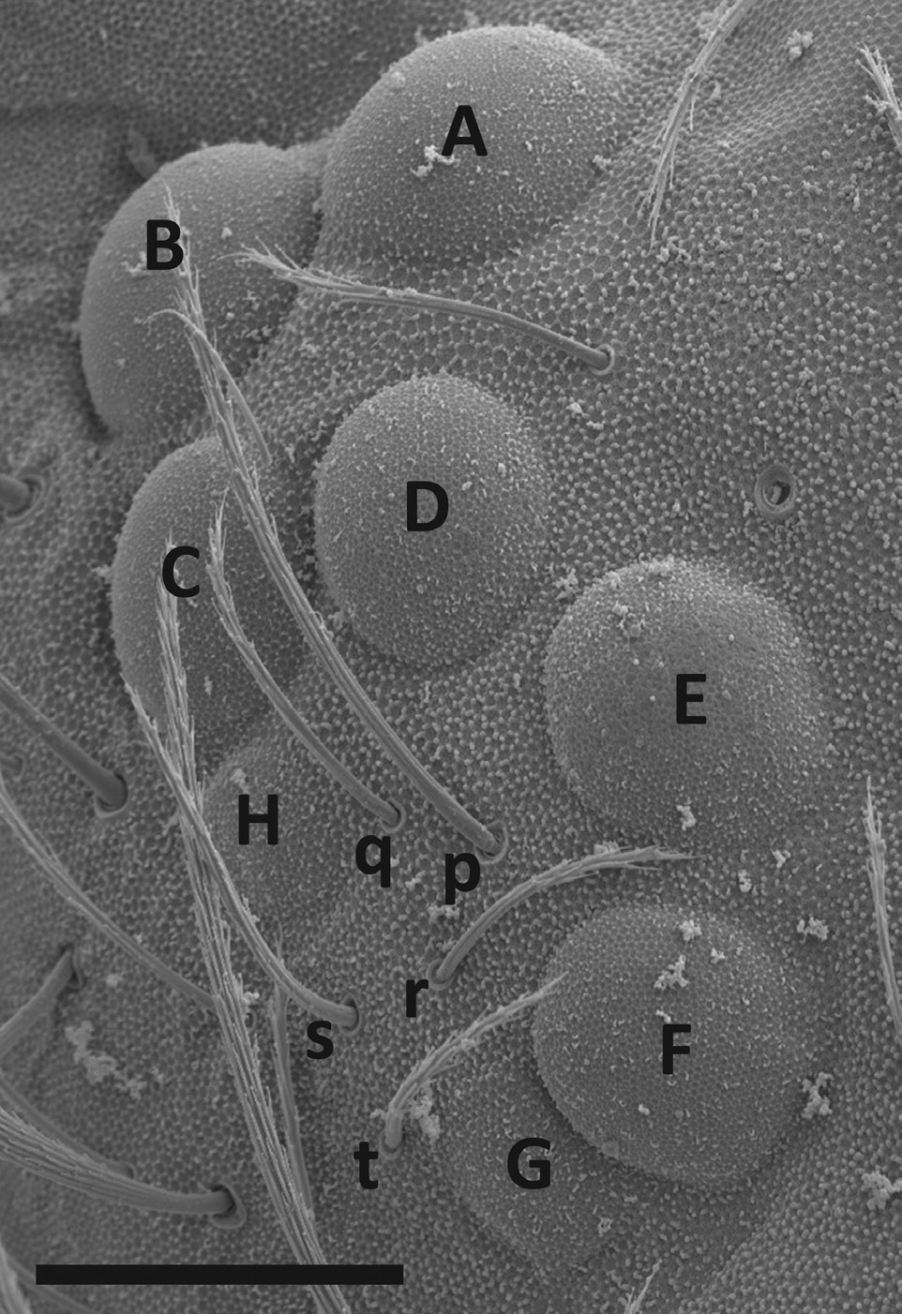
*Entomobrya atrocincta*. SEM photograph of right eye patch, eyes (A–H) and eye patch setae (p-t). Scale bar = 20 µm.

*Eye patch setae*: The number of setae in the eye patch (Fig. 1A) is important for distinguishing some species of *Entomobrya*. The number of setae vary from three to six, but five is most common. Eye patch setae nomenclature in this study follows Mari Mutt (1986).

*Prelabral and labral chaetotaxy*: The number of setae in the prelabral and three labral rows is 4,5,5,4, typical of Entomobryidae. However, the prelabral setae (row basal to labrum), which are usually ciliate, may appear to be smooth under low magnification for some species of *Entomobrya*.

**Figure 2.**
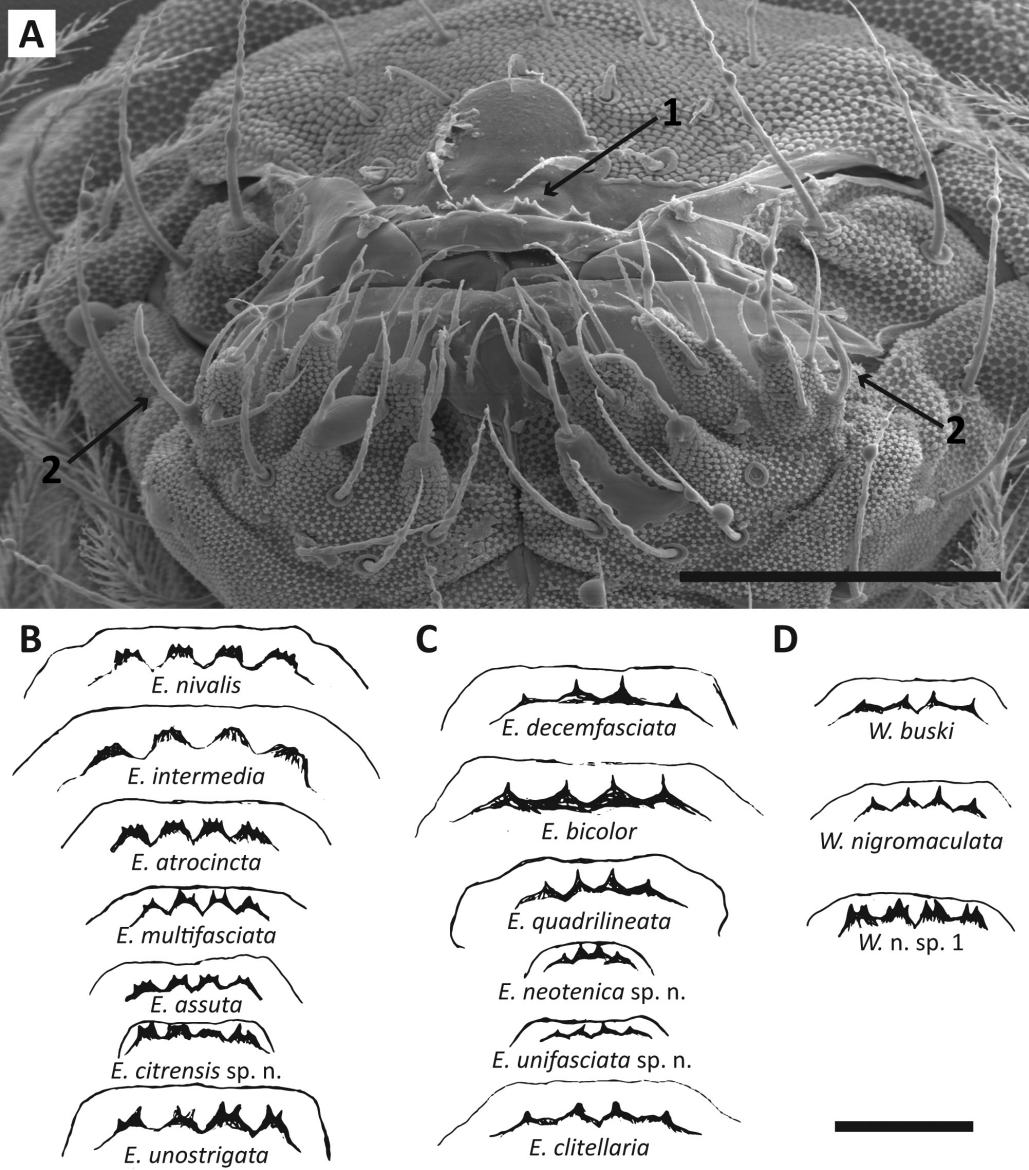
**A** *Entomobrya atrocincta*. SEM photograph of mouth cone, four labral papillae with multiple projections (1) (arrow points to 3^rd^ papilla from left to right) and labial appendage (2) **B–D** drawings of labral papillae of *Entomobrya* and *Willowsia* species: *Entomobrya* with multiple projections per papilla (column B); *Entomobrya* with a single projection per papillae (column C); labral papillae of all *Willowsia* species occurring in the Eastern United Sates (column D). Note *Willowsia* n. sp. 1 can be differentiated by the presence of multiple projections per papilla. *Entomobrya jubata* sp. n. and *Entomobrya ligata* are not included in figure but are similar to *Entomobrya clitellaria* and *Entomobrya unifasciata* sp. n. respectively. Scale bars = 20 µm.

*Labral papillae*: The morphology of the labral papillae (Fig. 2A–D) varies considerably among species of *Entomobrya* (Christiansen 1958b; South 1961) and is relatively easy to observe, thus, descriptions of the papillae are also included in the present study.

*Labial appendage*: Christiansen (1958b) incorporated the ratio of labial appendage on papilla E (Fig. 2A) to papilla length, even after recognizing the potential limitations of relative ratios (Christiansen 1954). The deformation of these soft body parts after slide mounting and pronounced variation between instars makes these measurements unreliable and not practical for diagnostic purposes (South 1961).

*Labial chaetotaxy*: Labial palp proximal setae, labial triangle setae, and post-labial setae have been shown to be taxonomically informative for other genera (Chen and Christiansen 1993, 1997; Soto-Adames 2010), but generally lack any useful variation in North American *Entomobrya*. For this study, all descriptions of labial chaetotaxy follow nomenclature developed by Chen and Christiansen (1993).

**Figure 3.**
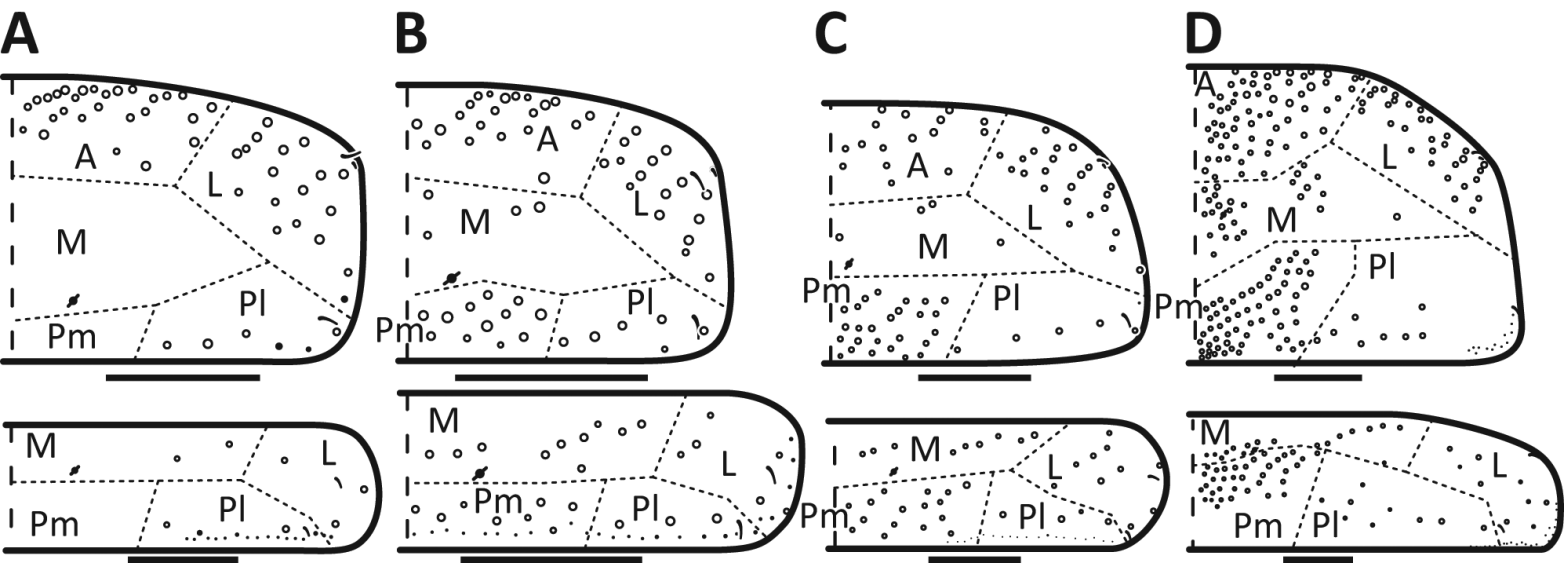
Differences of macrosetae abundance within thoracic chaetotaxy zones A, L, M, Pm, and Pl for Th. 2 (top) and Th. 3 (bottom): **A** *Entomobrya assuta* with the lowest number of macrosetae **B** *Entomobrya atrocincta* with a moderate number of macrosetae **C** *Entomobrya unostrigata* with a moderate number of macrosetae **D** *Entomobrya decemfasciata* with the most macrosetae. Marked differences in zone Pm for both segments clearly differentiate between some groups of *Entomobrya*. Scale bars = 100 µm.

*Dorsal chaetotaxy*: The introduction of “stable” character systems for dorsal macrosetae (Szeptycki 1979; Jordana and Baquero 2005; Soto-Adames 2008) have provided a large set of characters that are relatively easy to observe and compare among different species. *Entomobrya* are extremely setaceous (polychaetotic), providing many diagnostic characters. However, the abundance of macrosetae and observed variation among instars (including among adult instars) makes homology assessment of each element difficult. The macrosetae inserted external to the sensilla on abdominal segments (Abd.) 1-3 and external to the lateral bothriotricha on Abd. 4 were not included in descriptions or analysis due to extensive variation and difficulty of observation. The dorsal chaetotaxy of the head and along the dorsal median line on the mesothorax (Th. 2), metathorax (Th. 3), Abd. 1-3 and macrosetae internal to the lateral bothriotricha on Abd. 4 are easy to observe and relatively stable, thus are emphasized in the descriptions and analysis. The chaetotaxy of Abd. 5 lacks useful variation in specimens observed in this study. Thoracic zones (Fig. 3) originally described by Szeptycki (1979), differ in number of macrosetae among *Entomobrya* species and may provide some initial direction in the identification process.

*Trochanteral organ*: Differences in number and arrangement of small, spine-like setae on the trochanter, termed the trochanteral organ, have also been used to support the identification of some species (Christiansen 1958b). These setae are not only difficult to observe, but intraspecific variation limits their use for *Entomobrya* species delimitation (South 1961). However, the setal pattern, rather than the presences/absence or specific seta, seems to separate some species.

**Figure 4.**
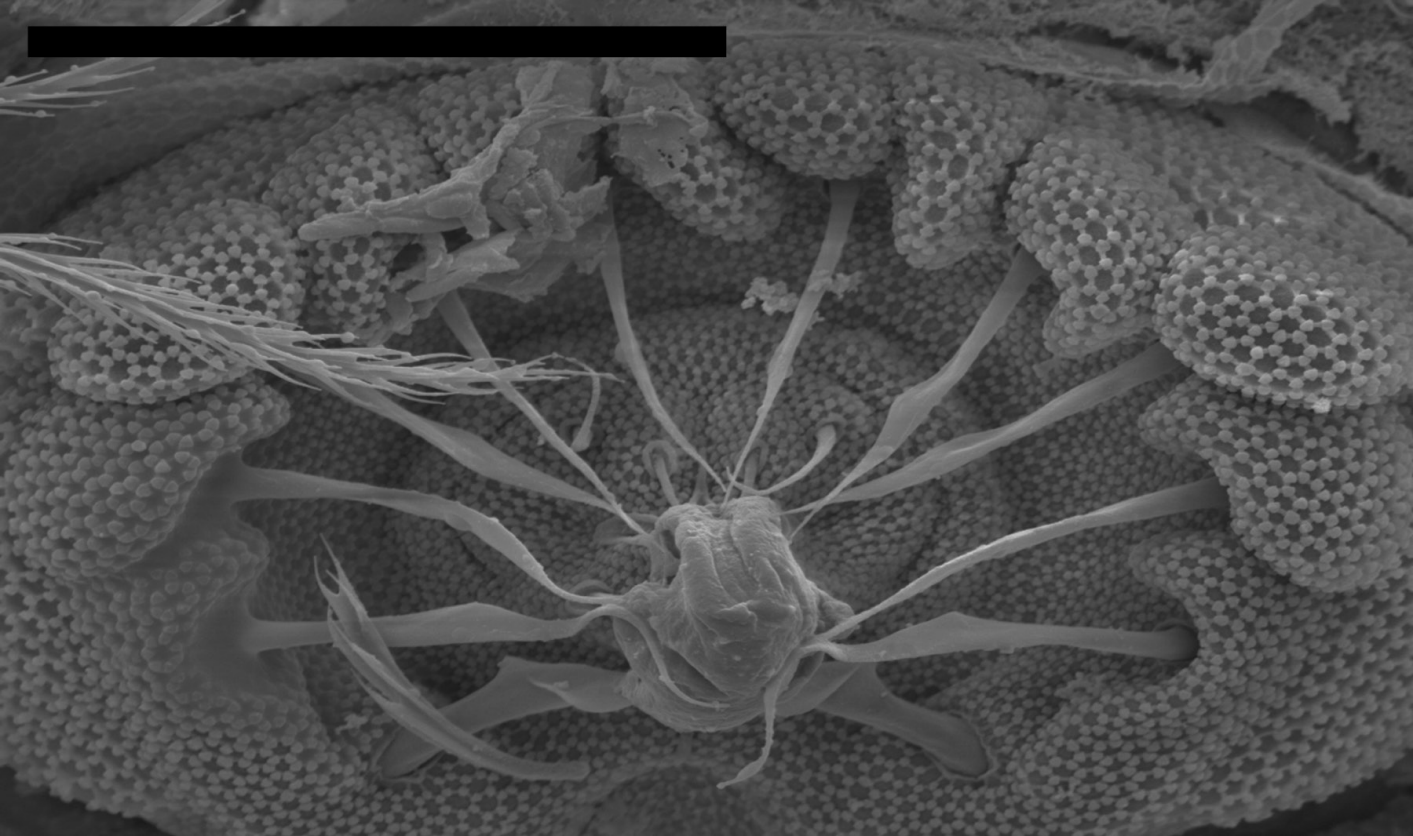
*Entomobrya atrocincta*. SEM photograph of papilate male genital plate. Scale bar = 20 µm.

*Male genital plate*: Differences in chaetotaxy of the male genital plate can accurately delineate many North American *Entomobrya* species (Christiansen 1958a, 1958b). However, males with a well-developed plate are uncommon in the samples examined and when present, plates are difficult to observe under light microscopy, requiring electron microscopy in order to easily discern characters with certainty (Fig. 4).

*Mucro and tarsal claw*: Ratios of relative positions of mucronal and ungual teeth do not usually deform by the mounting process, but they depend heavily on the angle or position of the slide mount in order to properly standardize relative measurements between individuals. Additionally, these characters present few discernable differences between *Entomobrya* species and have been noted to be of little taxonomic value for European *Entomobrya* (South 1961). There are marked differences in the distance between dorsal tooth and lateral teeth on the unguis between *Entomobrya* species. However, these measurements are difficult to quantify due to variation and mounting inconsistencies.

*Anatomical measurement ratios*: Some authors have used relative anatomical length or distance ratios for *Entomobrya* species separation (Christiansen and Bellinger 1998; Jordana 2012). Although measurement ratios may provide some level of diagnostic utility, high levels of variation in ratios (Christiansen 1954, 1958b), deformation of soft tissues, and variable final position of mounted specimens reduce character consistency and reliability, thus ratio measurements are not used here as diagnostic characters.

### Character nomenclature, abbreviations, and symbols

**Figure 5.**
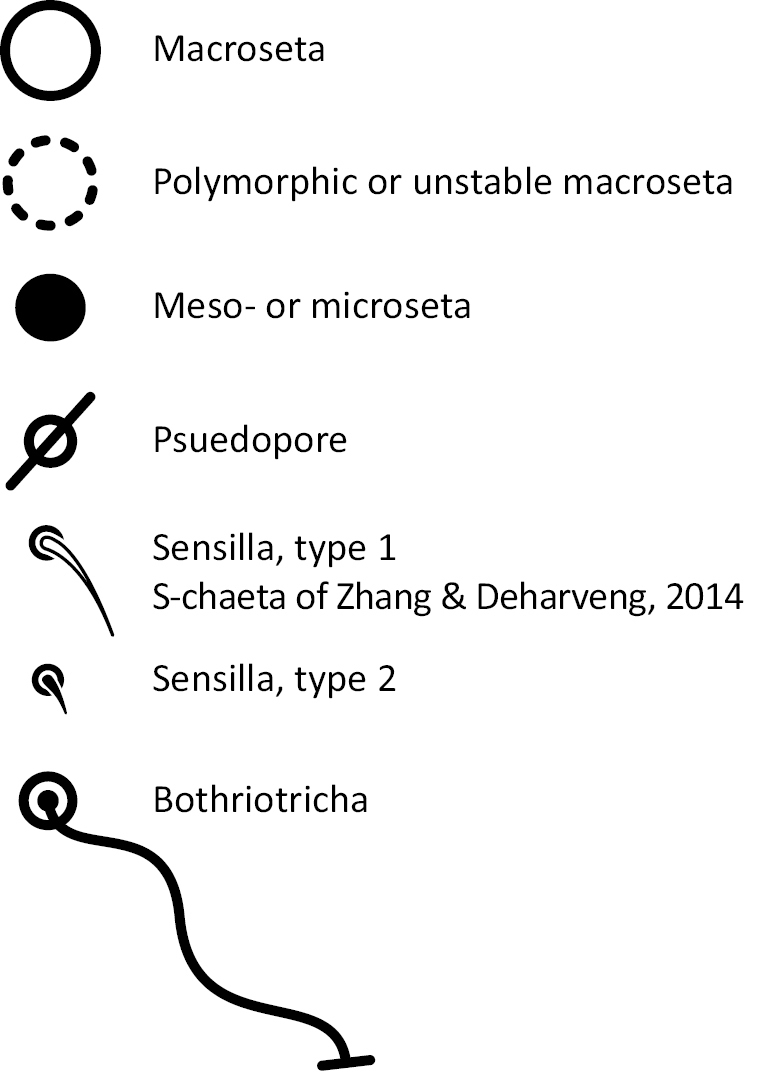
Symbol legend for diagrammatic figures of chaetotaxy presented in the species descriptions.

**Figure 6.**
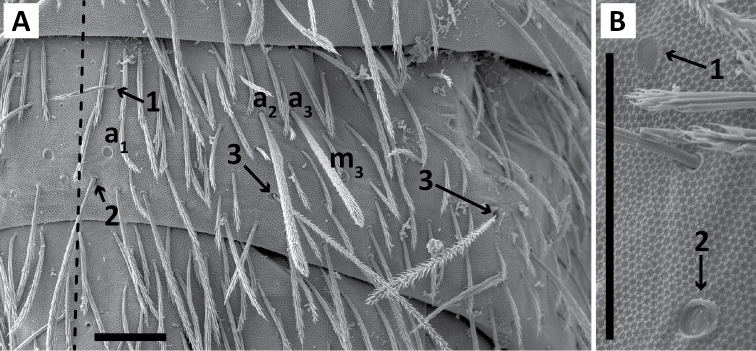
*Entomobrya atrocincta*. SEM photographs of selected morphological characters: **A** close-up view of 3^rd^ abdominal segment, macroseta (socket of a_3_ and m_3_ are labeled), type 5 microseta (1), mesoseta (2), bothriotricha (3), the dotted line represents the medial division of Abd. 3 **B** comparison between a pseudopore (1) and macroseta socket (2). Scale bars = 20 µm.

Descriptions of adult dorsal macrosetae provided in this study follow the dorsal trunk chaetotaxy nomenclature established by Szeptycki (1979) and the dorsal head chaetotaxy from Jordana and Baquero (2005) and Soto-Adames (2008). References to thoracic and abdominal segments are abbreviated as Th. (2-3) and Abd. (1-6) respectively. Symbols used in chaetotaxy descriptions are presented in Figure 5. There are seven general morphological structures, including three types of setae, recognized for the purpose of this study. Macrosetae are the primary setae, characterized by a large socket, long shaft, and are usually apically truncate or blunt (Fig. 6A). Microsetae are common type 5 (Christiansen 1958b), ciliate, short, thin, acuminate setae with very small sockets (Fig. 6A). Mesosetae are morphologically similar to microsetae, but are ostensibly longer, with larger sockets (Fig. 6A). They tend to be smaller and thinner than macrosetae, but substantial variation in length and size of both setae types cause overlap. Bothriotricha (Fig. 6A) are specialized setae characterized by unique morphology; long, thin, with conspicuous ciliation. There are also two types of short, smooth, acuminate, spine-like sensilla: type 1 (S-chaeta of Zhang and Deharveng 2015) is long and more common and occur on all thoracic and abdominal segments; while type 2 (S-microchaeta of Zhang and Deharveng 2015) is short, sometimes slightly blunted, usually paired with type 1, and is only present on Th. 2, Abd. 1, and Abd. 3. Pseudopores are relatively difficult to observe and resemble sockets of macroseta, but are generally shallower and lack a thickened socket wall (Fig. 6B).

### Phylogenetic analysis

In order to investigate the effects chaetotaxy and other morphological characters have on phylogenetic relationships, Bayesian and maximum likelihood phylogenetic analyses were conducted using MrBayes v. 3.2.1 (Ronquist et al. 2012) and RAxML v. 8.1.20 (Stamatakis 2014); one based only on morphological characters and an additional analysis based on combined morphology and molecular datasets.

The morphological analysis of 22 taxa, including 14 species of *Entomobrya* and 8 additional species, was based on 179 morphological characters. *Pseudosinella violenta* (Folsom) was selected as the outgroup. Character state assignments (Suppl. material 1) were attained through observation of material collected for this study, except for *Pseudosinella violenta* and *Seira dowlingi* (Wray), which were obtained from Soto-Adames (2010) and Soto-Adames (2008) respectively. Morphological characters were analyzed under the Mk model of evolution (Lewis 2001), with rate variation among characters (gamma distribution), four independent runs, starting from random trees, four Markov chains (temp=.5), and 25,000,000 generations, sampling every 1000^th^ generation. Default values were used for all other parameters.

The combined analysis incorporating complete COI sequences (1539 bp) for 89 exemplars (See Katz et al. 2015 for specific details regarding specimens, GenBank accession numbers, gene choice, DNA extraction, amplification, sequencing, and primer development) with *Pseudosinella violenta* as the outgroup. The appropriate model of sequence evolution (GTR+I+G), was selected using jModeltest (Posada 2008), whereas the Mk model (Lewis 2001) was implemented for the morphology dataset. Morphology was not examined for sequenced individuals due to the destructive process of DNA extraction; therefore character states were attained from other individuals of the same species or from descriptions in the literature (Soto-Adames 2008, 2010) and were added to corresponding OTU’s in the matrix. Bayesian analyses were conducted using MrBayes on the CIPRES Science Gateway (Miller et al. 2010), convergence was assessed by observation of average standard deviation of split frequencies values below p < 0.003, a 25% burn-in was used, and all posterior probabilities and consensus trees were computed in MrBayes. The maximum likelihood analyses were conducted using RAxML’s rapid bootstrap algorithm incorporating 1000 bootstrap replicates.

### Character state assignments

Dorsal setae were identified by their relative positions to bothriotricha, sensilla, pseudopores, and to neighboring setae, following descriptions provided by Szeptycki (1979), Jordana and Baquero (2005), and Soto-Adames (2008). All macrosetae labeled in figures for species descriptions were included as characters in the phylogenic analysis (See Suppl. material 1 for morphological character matrix). Macrosetae external to sensillum on Abd. 1-3 and external to bothriotricha on Abd. 4 were not included in descriptions or analysis due to uncertain homology. Characters states for setae were defined as present, absent, or polymorphic (i.e., variable between individuals of the same species). Macrosetae were considered absent if meso- or microsetae were present in corresponding position. Polymorphic states are indicated in the figures as dotted circles. Other morphological characters included in the phylogenetic analysis are number of eye patch setae, labral setae (smooth or ciliate), labral papillae (smooth, single projection, or multiple projections), setae within labial triangle (ciliate or smooth), antennal bulb (absent or present), scales (absent or present), and dental spines (absent or present). See Suppl. material 1 for a complete list of all 179 morphological characters and character states circumscription used in this analysis.

## Results

### Species descriptions and taxonomy

#### Family Entomobryidae Schäffer, 1896
Entomobryini Schäffer, 1896 sensu Soto-Adames et al. 2008
*Entomobrya* Rondani, 1861

This genus characterized by having 8+8 eyes within black or dark blue patches of pigment, a bidentate mucro with a smooth basal spine, basic chaetotaxy formed by type 5 microsetae and the absence of antennal sub-segmentation, scales, dental spines, and differentiated “smooth” setae on the inner surface of the hind tibiotarsus.

In addition, all species treated here have an apical antennal bulb; main sensilla on 3^rd^ antennal segment sense organ thin, smooth, blunt and peg-like; differentiated smooth setae on ventral side of 1^st ^antennal segment of two types, short and spine-like and long and seta-like; labral setae 5,5,4 and smooth; outer maxillary lobe of maxilla with subapical and apical setae smooth and subequal, and sublobal plate with three smooth seta-like appendages; lateral appendage of labial papilla E slightly curved, relatively thick, blunt; all post labial setae type 5; unguis with one outer, two lateral and four inner teeth, and a lanceolate unguiculus; and mucronal spine smooth; and most have all posterior setae of labial triangle ciliate, as M1, r, E, L1, L2, with r significantly smaller than other setae and A1-A5 smooth.

More general morphological descriptions of this genus are provided in Christiansen (1958b), Stach (1963), Christiansen and Bellinger (1998), and Jordana (2012). Chaetotaxy provided in the following descriptions follows the nomenclatural systems established by Szeptycki (1979) and Jordana and Baquero (2005) with some modifications.

Informative diagnostic adult chaetotaxy characters of all *Entomobrya* species treated here are listed in Table 2. These characters were specifically chosen for their ease of observation, stability (lack of polymorphisms), and confident homology.

**Table 2.** Informative diagnostic adult chaetotaxy for the separation of all *Entomobrya* species in this study^5,6^. These characters were specifically chosen for their ease of observation, stability, and lack of polymorphic states. Refer to species remarks for more specific diagnostic characters. Parentheses indicate a rarely observed state.

	Head macrosetae^1^					Th. 2 macrosetae^1^		Abd. 2 macrosetae^1^			Abd. 3 macrosetae^1^		Prelabral setae^2^	Number of eye patch setae^3^	Labral papilla^4^
Species	M_3i_	S’_0_	S_4i_	Ps_3_	Ps_5_	m_2_	m_5_	a_2_	a_3_	m_3ep_	a_1_	a_2_			
*Entomobrya assuta*	0	0	1	0	1	0	0	0	0	0	0	1	0	5	0
*Entomobrya atrocincta*	0(1)	0	1	0	1	1	0	1	1(0)	0(1)	1	1	1	5	0
*Entomobrya bicolor*	1	0	1	0	0	1	1	1	1	1	0	1	1	5	1
*Entomobrya citrensis* sp. n.	0	0	1	1	1	0	0	0	1	0	0	1	0	5	0
*Entomobrya clitellaria*	0	0	1	0	1	1	1	1	1	1	0	1	1	5	1
*Entomobrya decemfasciata*^5^	1	0	1	0	1	1	1	1	1	1	0	1	1	5	1
*Entomobrya intermedia*	0	0	0	0	1	1	1	1	1	0	1	0	1	5	0
*Entomobrya jubata* sp. n.	0	1	1	0	0	1	1	1	1	1	0	1	1	5	1
*Entomobrya ligata*^6^	0	0	1	0	1	1	0	1	0	1	0	0	0	3	1
*Entomobrya multifasciata*	0	0	0	0	1	1	0	1	1	0	1	1	1	5	0
*Entomobrya neotenica* sp. n.	0	0	0(1)	0	1	1	0	1	0	0	0	0	0	3	1
*Entomobrya nivalis*	0	0	1	P	1	1	1	1	1	1	1	0	1	5(6)	0
*Entomobrya quadrilineata*^5^	1	0	1	0	1	1	1	1	1	1	0	1	1	5(6)	1
*Entomobrya unifasciata* sp. n.^6^	0	0	1	0	1	1	0	1	0(1)	1	0	0	0	3	1
*Entomobrya unostrigata*	0	0	1	0	1	0	1	1	1	1	1	0	1	5	0

1 Character states: absent (0), present (1), polymorphic (P).

2 Character states: smooth or finely ciliate (0), ciliate (1).

3 Refer to Figure 1 for SEM photograph of eye patch setae.

4 Character states: multiple projections (0), single projection (1). Refer to Figure 2 for SEM photograph and drawings of labral papillae.

5 *Entomobrya decemfasciata* and *Entomobrya quadrilineata* cannot be separated by these diagnostic characters. Color pattern is critical for diagnosis.

6 *Entomobrya ligata* and *Entomobrya unifasciata* sp. n. cannot be separated by these diagnostic characters. Color pattern is critical for diagnosis.

##### Entomobrya assuta

Taxon classificationAnimaliaCollembolaEntomobryidae

Folsom, 1924

**Figure 7.**
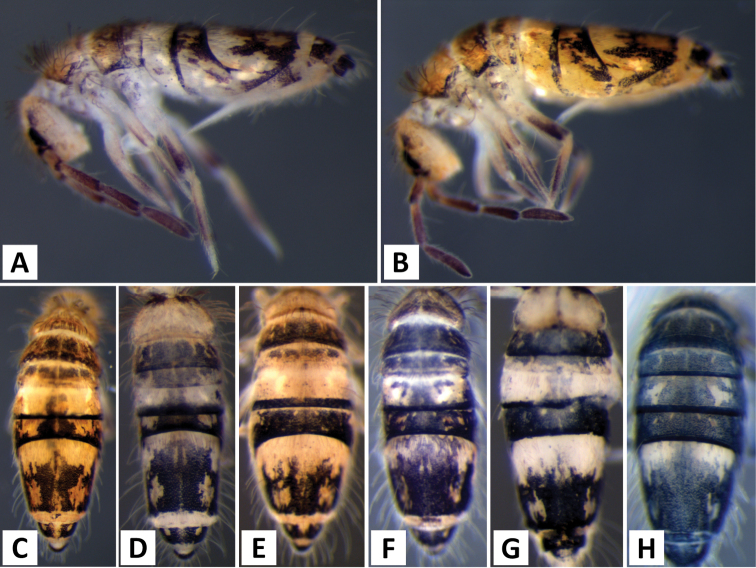
Color patterns of *Entomobrya assuta*. Photographed specimens are from the following localities: **A** Champaign Co., IL **B** Champaign Co., IL **C** Champaign Co., IL **D** Knox Co., TN **E** Knox Co., TN **F** Mason Co, IL **G** Knox Co., TN **H** Iroquois Co., IL.

**Figure 8.**
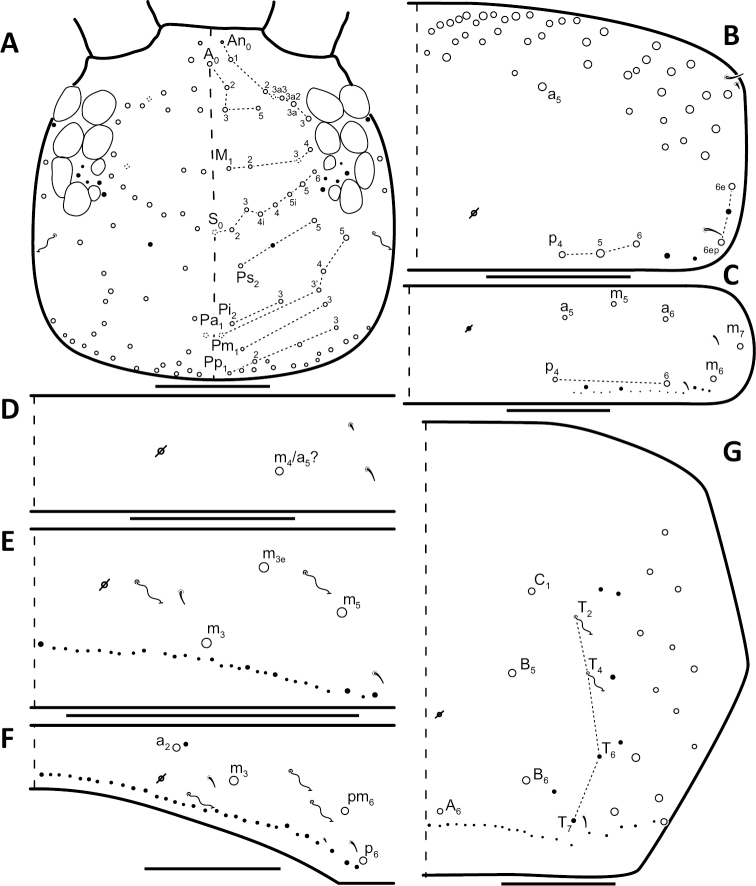
Dorsal chaetotaxy of *Entomobrya assuta*: **A** Head **B** Mesothorax **C** Metathorax **D** 1^st^ abdominal segment **E** 2^nd^ abdominal segment **F** 3^rd^ abdominal segment **G** 4^th^ abdominal segment. Scale bars = 100 µm. See Figure 5 for symbol legend.

Figs 2 , 3A , 7 , 8 , 39

###### Description.

*Body shape and color pattern*. Body dorso-ventrally flattened. Dorsal color pattern highly variable, and in many cases, without clear discrete forms (Fig. 7). Patterns usually consisting of black or dark blue pigment on a white, yellow, orange, or light purple background. Thorax pigmentation variable. Sometimes Th. 2 and Abd. 3 entirely dark, forming two strong transverse bands. All forms studied have a dark transverse band across the posterior margin of Abd. 2. Abd. 3 entirely dark or with two or three pale spots. Abd. 4 and 5 also with 2-3 pale spots each. Posterior margin of Abd. 4 usually lacking pigment, forming an irregular pale transverse band. Antennae usually entirely purple, but 1^st^ antennal segment at times considerably lighter.

*Head*. Apical bulb of 4^th^ antennal segment usually bilobed, sometimes simple or, rarely, trilobed. Long differentiated smooth setae on ventral side of 1^st ^antennal segment ≈3x short setae. Prelabral setae finely ciliated, seemingly smooth at low magnification under light microscopy. Distal margin of labral papillae with 2-3 small spine-like projections (Fig. 2). Dorsal head chaetotaxy reduced in comparison with other species (Fig. 8A); macrosetae An’_0_, A_6_, M_3i_, S’_0_, S_1_, Pi_1_, Pa_2_, Pa_3_, Pm_2_, and Pm_1i_ always absent; An_3a3_ seen in one individual; M_3_ present in 1/4 of individuals observed; S_0_ and Pa_1_ usually present. Eyes G and H small and subequal. Eye patch with 5 setae.

*Thorax*. Thoracic chaetotaxy extremely reduced but stable, without macrosetae variation in specimens studied. A row of microsetae present along entire posterior margin of Th. 2 and Th. 3 (not displayed in figures). Th. 2, with a_5_ and 5 posterior macrosetae (Fig. 8B): p_4_, p_5_, p_6_, p_6e_, and p_6ep_. Th. 3, with 7 macrosetae (Fig. 8C): a_5_, a_6_, m_5_, m_6_, m_7_, p_4_, and p_6_; macrosetae a_1_, a_2_, and a_3_ absent. Chaetotaxy of zone Pm extremely reduced for both thoracic segments, without macrosetae (Figs 3A; 8B,C).

*Legs*. Trochanteral organ with triangular setal pattern and up to 23 setae. Unguis with 4 inner teeth; basal teeth located approximately middle of inner claw length.

*Abdomen*. Abdominal chaetotaxy reduced but stable, no macrosetae variation observed. Abd. 1 with 1 macroseta only (Fig. 8D); row of microsetae along entire posterior margin present (not displayed in figure). Abd. 2 with 3 macrosetae: m_3_, m_3e_, and m_5_ (Fig. 8E). Abd. 3 with 4 macrosetae: a_2_, m_3_, pm_6_, and p_6_. Mesosetae a_2a_, inserted slightly anterior and exterior to a_2_, sometimes with relatively large socket resembling socket of macroseta a_3_, but due to mesoseta a_2a_’s close proximity to a_2_, it is most likely a duplicate of a_2_ rather than homologous to a_3_ (Fig. 8F). Abd. 4 with 4 inner macrosetae (Fig. 8G). Mucronal teeth subequal.

###### Remarks.

*Entomobrya assuta* is the only species with the color pattern as described above in combination with the absence of all the following macrosetae: head Ps_3_, Th. 2 m_2_ and m_3_, Abd. 2 a_2_, a_3_ and m_3ep_, and Abd. 3 a_1_ (see Table 2 for additional diagnostic characters). Among all species observed in this study, only *Entomobrya assuta* and *Entomobrya citrensis* sp. n. share this unique pattern of substantially reduced dorsal chaetotaxy. *Entomobrya assuta* and *Entomobrya citrensis* sp. n. share very similar chaetotaxy and color pattern. In fact, *Entomobrya citrensis* sp. n. was at first considered to be an undescribed color form of *Entomobrya assuta*, but molecular data provides evidence for their separation (Katz et al. 2015).

Subsequent comparative morphological observations between the two forms show that head macroseta Ps_3_ and Abd. 2 macroseta a_3_ are both absent in *Entomobrya assuta*, but present in *Entomobrya citrensis* sp. n.; additionally, *Entomobrya citrensis* sp. n. has a complete, dark transverse band located medially across Abd. 4, whereas this band is absent in *Entomobrya assuta*. Labral morphology also separates these species, *Entomobrya assuta* has relatively uniform labral papillae, each with two to three seta or spine-like projection, whereas *Entomobrya citrensis* sp. n. has up to five minute bumps or serrations on the two internal papillae and only two larger spine-like projections on the two external papillae (Fig. 2). It should be pointed out that the last character may be variable and should be used in combination with chaetotaxy and color pattern for diagnosis.

Christiansen and Bellinger (1998) reported seven different color forms of *Entomobrya assuta*, each occurring in separate localities across North America. Many of these color forms were not sampled for this study, and in view of the discovery of *Entomobrya citrensis* sp. n. it is possible that some of them may represent distinct species. Future determinations of *Entomobrya assuta* will have to be based on analysis of chaetotaxy and other morphological characters outlined in the present description and not just color pattern.

*Entomobrya assuta* appears to be an intrusion of a southern subtropical or tropical *Entomobrya* lineage into the Nearctic region. Christiansen and Bellinger (1998) noted that the dorsal and genital chaetotaxy of *Entomobrya assuta* is more similar to tropical rather than Nearctic species. In fact, the reduction in chaetotaxy approaches that seen in *Entomobrya longiseta* Soto-Adames and *Entomobrya linda* Soto-Adames from the Caribbean, more than other Nearctic forms.

###### Distribution.

Endemic to North America (Suppl. material 2: A).

###### Material examined.

USA: *Cotypes* (J. W. Folsom), 2 on slide, Vermont, Clarendon, under bark Mar-April 1898 (O. W. Barrett), INHS Cat. No. 528,332; *Cotypes* (J. W. Folsom), 5 in vial, same information as above; *Cotypes* (J. W. Folsom), 4 in vial, Geneva, N. Y., June 18, 1917, beneath apple bark (H. Glasgow). Other material examined: 2 in vial, Alabama, Covington Co., Conecuh National Forest, off Co. Rd. 11 (31.07900,-86.61203), under bark, 2.i.2012 (A. Katz & M. DuBray), AK12-6; 3 on slides, 20 in vial, Florida, Taylor Co., Econfina State Park (30.0656,-83.91066), under bark, 9.viii.2011, AK11-116; 2 in vial, Illinois, Champaign Co., Champaign, Kaufman Lake Park (40.11514,-88.29000), in bird nest, 8.v.2011, AK11-26; 3 on slides, 5 in vial, Illinois, Champaign Co., Urbana, Brownfield Woods (40.14462,-88.16543), on low-lying vegetation, 7.vii.2011, AK11-60; 1 on slide, Illinois, Champaign Co., Urbana, bird nest, 28.ix.1957 (R. Hurley); 5 on slides, Illinois, Iroquois Co., Iroquois County Forest Preserve (40.99279,-87.59734), under bark, 19.viii.1989 (F. Soto-Adames); 1 in vial, Illinois, Jasper Co., Sam Parr State Fish and Wildlife Area (39.03293,-88.12516), under bark, 15.vii.2011 (A. Katz & F. Soto-Adames), AK11-73; 2 in vial, Illinois, Jo Davies Co., South Blanding Rd., Stevenson Property (42.29895,-90.36967), under bark, 27.viii.2011, A11-148; 1 on slide, 6 in vial, Illinois, Jo Davies Co., Princess Mine 1 (42.30565,-90.39740), under bark, 26.viii.2011, AK11-142; 1 in vial, Illinois, Jo Davies Co., Princess Mine 1 (42.30565,-90.39740), beating vegetation, 26.viii.2011, AK11-143; 4 in vial, Illinois, Jo Davies Co., South River Rd., Asgard Vent 1 (42.30170,-90.40345), under bark, 27.viii.2011, AK11-153; 1 on slide, 7 in vial, Illinois, Jo Davies Co., South River Rd., Asgard Vent 2 (42.30378,-90.40108), under bark, 27.viii.2011, AK11-157; 1 on slide, Illinois, Kankakee Co., Kankakee River State Park (41.19482,-87.96875), from bird nest, 10.iv.2011, AK11-6; 1 on slide, Illinois, Kankakee Co., Kankakee River State Park (41.19482,-87.96875), from squirrel nest, 10.iv.2011, AK11-8; 1 in vial, Illinois, Mason Co, Revis Hill Nature Prairie Reserve (40.15246,-89.85330), beating vegetation, 18.vii.2011 (A. Katz & F. Soto-Adames), AK11-86; 2 on slides, 4 in vial, Illinois, Mason Co., Sand Ridge State Park (40.40892,-89.87590), beating branches, 18.vii.2011 (A. Katz & F. Soto-Adames), AK11-82; 2 on slides, 8 in vial, Illinois, Piatt Co., Lodge Park (40.06709,-88.56596), under bark, 23.vii.2011, AK11-100; 1 in vial, Illinois, Pike Co., Lincoln’s New Salem State Park (39.96868,-89.83386), beating grasses, 18.vii.2011 (A. Katz & F. Soto-Adames), AK11-90;1 on slide, 3 in vial, Illinois, Pope Co., Lake Glendale (37.41350,-88.65982), under bark, 24.ix.2011, AK11-160; 1 on slide, Illinois, Vermilion Co., University of Illinois Observatory, Nixon Fork, leaf littler, 25.iv.2009 (F. Soto-Adames & L. Deem), FD09-25; 1 in vial, Illinois, Vermilion Co., Kennekuk Cover County Park, Windfall Prairie Nature Preserve (40.20995,-87.74181), aspirated from bushes, 16.vi.2011 (A. Katz & F. Soto-Adames), AK11-59; 3 on slides, Illinois, Will Co., Braidwood (41.25118,-88.19494), soil and leaf litter, 6-8.ix.2011 (F. Soto-Adames); 2 on slides, Michigan, Ingham Co., Michigan State University, Baker Wdlt. (42.66527,-84.36264) under bark of standing dead pine, 24.vii.2008 (E. C. Bernard), BW-11; 1 on slide, Michigan, St. Clair Co., Algonac State Park (42.65447,-82.52430), under bark of recently fallen maple, 25.vii.2008 (E. C. Bernard), ASP-15; 8 in vial, Pennsylvania, Chester Co., Wayne, sweep of *Forsythia* sp., 29.vi.2012, AK12-50; 7 on slides, 30+in vial, Tennessee, Knox Co., University of Tennessee, Ag. Campus, Morgan Hall (36.01023,-83.93829), on moist fallen bark, April 2010 (E. C. Bernard).

##### Entomobrya atrocincta

Taxon classificationAnimaliaCollembolaEntomobryidae

Schӧtt, 1896

Figs 1 , 2 , 3B , 4 , 6 , 9 , 10 , 39

###### Description.

**Figure 9.**
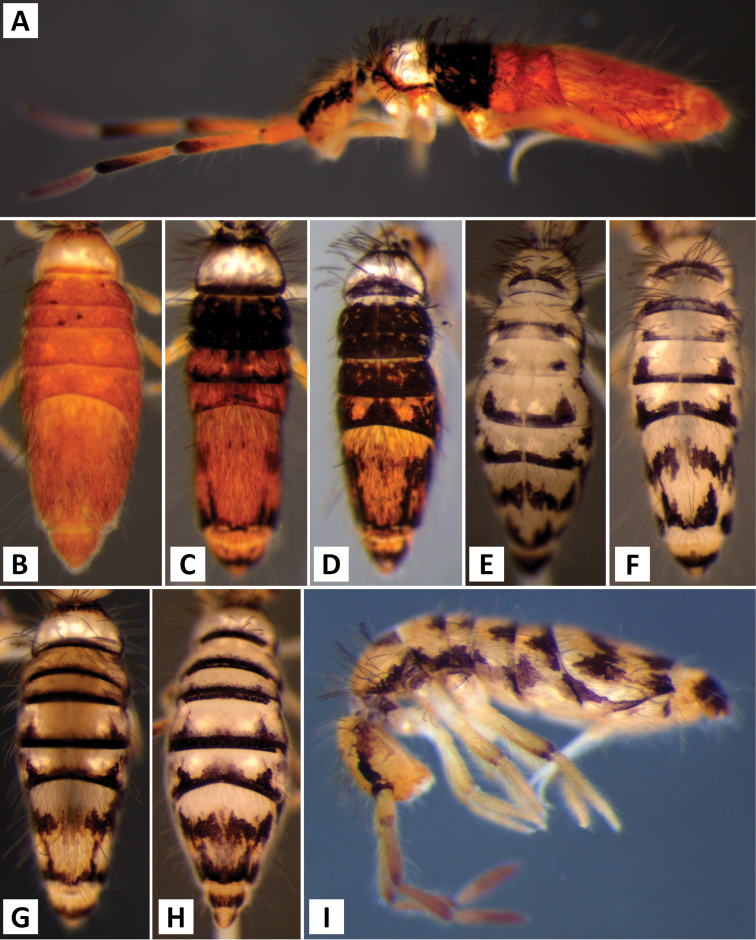
Color patterns of *Entomobrya atrocincta*: **A–D** Male color forms **E–I** Female color forms. All photographed specimens were collected from Champaign Co., IL.

*Body shape and color pattern*. Sexually dimorphic in color pattern and body shape. Males and females with variable but characteristically different color patterns (Fig. 9). Male body relatively cylindrical, slender, with bright orange background with black pigment usually forming a thick and complete transverse dorsal band covering posterior margin of Th. 2 and all of Th. 3 and Abd. 1, band sometimes absent. Male light form without dark pigment on Th. 2 through Abd. 6 (except sometimes along anterior margin of Th. 2). Male dark form with band covering Th. 2 through Abd. 2, irregular pigment patterns sometimes forming 1+1 orange spots on Abd. 3, and two narrow longitudinal stripes connected by transverse band on posterior margin of Abd. 4 (Fig. 9A–D). Females with slightly dorso-ventrally flattened body and slightly larger than males. Female color pattern strikingly different from males, white or light yellow background with black, dark blue or purple pigment forming transverse bands across the posterior margins of Th. 2 through Abd. 4. All females with two longitudinal stripes or triangular extensions connected by two transverse bands on Abd. 4; one incomplete medial band and another complete band on posterior margin of Abd 5 (Fig. 9E–I). Mesonotum white in both sexes, lacking pigment except for a small irregular band across anterior margin. Medial area of Th. 2 relatively transparent and fat bodies visible through cuticle under a dissecting microscope. Both males and females with purple pigment usually extending from apical end of 2^nd^ antennal segment through apex of 4^th^ antennal segment.

**Figure 10.**
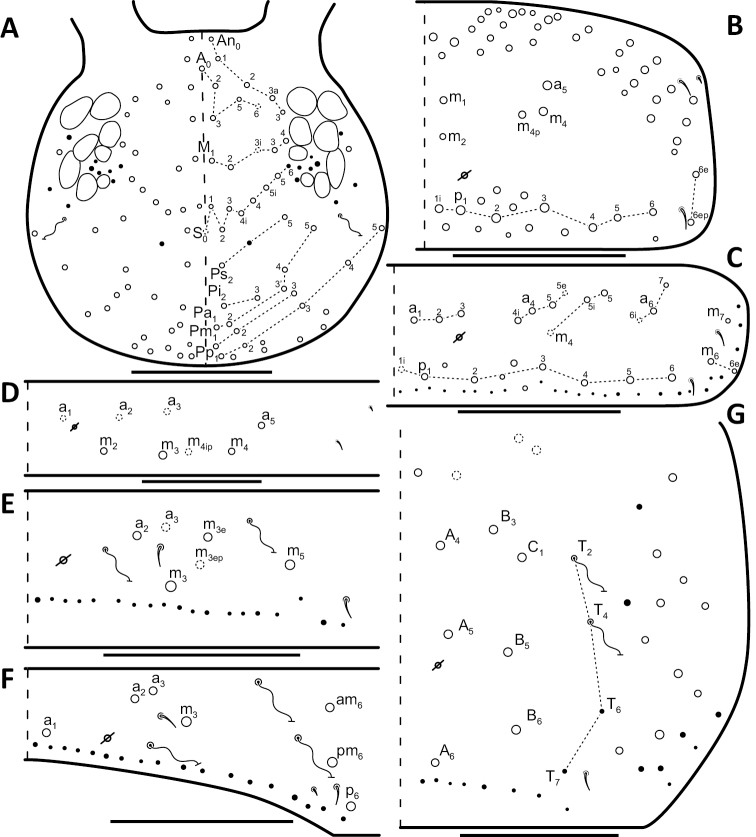
Dorsal chaetotaxy of *Entomobrya atrocincta*: **A** Head **B** Mesothorax **C** Metathorax **D** 1^st^ abdominal segment **E** 2^nd^ abdominal segment **F** 3^rd^ abdominal segment **G** 4^th^ abdominal segment. Scale bars = 100 µm. See Figure 5 for symbol legend.

*Head*. Apical bulb of 4^th^ antennal segment located in deep pit, usually simple, sometimes with up to four distinct lobes. Long differentiated smooth setae on ventral side of 1^st ^antennal segment ≈2.5x short setae. Prelabral setae ciliate. Ornamentation of distal margin of labral papillae with 3-4 small seta or spine-like projections (Fig. 2). Dorsal head chaetotaxy variable (Fig. 10A); macrosetae An’_0_, An_3a2_, An_3a3_, S’_0_, S_6_, Ps_3_, Pi_1_, Pa_3_, and Pm_1i_ always absent; S_0_ usually present, M_3i_ usually absent, A_6_ present in roughly half of observed specimens. Eyes G and H small and subequal; eye patch with 5 setae.

*Thorax*. Chaetotaxy of Th. 2 stable, without variation in number of macrosetae. A row of microsetae occurs along entire posterior margin of Th. 2 (not displayed in figure); a_5_, m_1_, m_2_, m_4_, m_4p_, and all posterior macrosetae (series Pi, Pa, Pm, and Pp) present (Fig. 10B). Chaetotaxy of Th. 3 variable: 8 macrosetae always present in zone M, 5 in zone L, and 3 in zone Pl (Fig. 3B); macroseta a_5e_, m_4_, a_6i_, and p_1i_ usually present (Fig. 10C).

*Legs*. Trochanteral organ with triangular setal pattern and up to 30 setae. Unguis with 4 internal teeth; basal teeth located approximately middle of inner claw length.

*Abdomen*. Abdominal chaetotaxy variable. Abd. 1, with 4-8 macroseta (Fig. 10D), row of microsetae present along entire posterior margin (not displayed in figure). Abd. 2 with 4-6 macrosetae (Fig. 10E): a_2_, m_3_, m_3e_, and m_5 _always present; a_3 _and m_3ep_ polymorphic. Abd. 3 chaetotaxy stable, with 7 macrosetae: a_1_, a_2_, a_3_, m_3_, am_6_, pm_6_, and p_6_ (Fig. 10F). Abd. 4 inner macrosetae varying from 8-11 (Fig. 10G). Basal mucronal tooth slightly larger.

###### Remarks.

*Entomobrya atrocincta* can be distinguished by the male or female color patterns as described above combined with the presence of macrosetae head S_4i_ and Abd. 3 a_1_ and a_2_ and the absence of macrosetae head ps_3_ and Th. 2 m_5_ (see Table 2 for additional diagnostic characters). The color pattern of female *Entomobrya atrocincta* is virtually indistinguishable from that of *Entomobrya multifasciata* and very similar to *Entomobrya intermedia*, and *Entomobrya nivalis*. Though these species may exhibit slight differences in color pattern, it is easier to differentiate them with the characters outlined in Table 3. Female *Entomobrya multifasciata* and *Entomobrya atrocincta* can be separated by the presence of head macroseta S_4i_ in *Entomobrya atrocincta* and its absence in *Entomobrya multifasciata*, and morphology of the labral papillae; *Entomobrya multifasciata* has 2-3 large seta or spine-like projections per papillae, whereas *Entomobrya atrocincta* has 3-4 small, seta or spine-like projections per papillae (Fig. 2).

**Table 3.** Diagnostic characters to separate species within the nivalis complex: *Entomobrya atrocincta*, *Entomobrya multifasciata*, *Entomobrya intermedia*, and *Entomobrya nivalis*. Diagnostic characters of *Entomobrya clitellaria* are also included in relation to *Entomobrya atrocincta* due to similarity in color pattern.

Species	Head macroseta S_4i_^1^	Th. 2 macroseta m_5_^1^	Abd. 2 macroseta m_3ep_^1^	Abd. 3 macroseta a_1_^1^	Abd. 3 macroseta a_2_^1^	Labral papillae^2^
*Entomobrya atrocincta*	1	0	0(1)^3^	1	1	0
*Entomobrya multifasciata*	0	0	0	1	1	0
*Entomobrya intermedia*	0	1	0	1	0	0
*Entomobrya nivalis*	1	1	1	1	0	0
*Entomobrya clitellaria*	1	1	1	0	1	1

1 Character states: absent (0), present (1).

2 Character states: multiple projections (0), single projection (1).

3 Parentheses indicate a rarely observed state.

The male form always has a unique orange color, but the distribution of purple pattern is variable. Christian and Bellinger (1998) report four discrete color forms. An additional male color form was collected during this study. The different color forms can commonly be found together within the same population. There are no significant morphological or COI DNA sequence differences between male forms and variation in color pattern (male and female) is attributed to intraspecific variation (Katz et al. 2015). The most common male form collected in this study (Fig. 9A) is somewhat similar to *Entomobrya clitellaria*. However, the two forms can be easily separated by chaetotaxy (Table 3).

Sexual dimorphism in this species has caused serious taxonomic confusion due to the similarity of female pattern to *Entomobrya multifasciata* and *Entomobrya nivalis*. Ramel et al. (2008) first described the sexual dimorphism of *Entomobrya atrocincta* from Greece and even noted that records of *Entomobrya multifasciata* may be misidentified *Entomobrya atrocincta* females. However, Jordana (2012) separated most European specimens and classified them as *Entomobrya nigrocincta* Denis based on chaetotaxy, synonymizing Ramel et al.’s (2008) descriptions with *Entomobrya nigrocincta*. Jordana (2012) attributes the sexual dimorphism (the same displayed by the specimens collected in North America and included in this study) to *Entomobrya nigrocincta* only, keeping the description by Christiansen (1958b) valid for *Entomobrya atrocincta*. Molecular data confirm that different color forms represent different sexes of the same species (Katz et al. 2015), demonstrating the presence of sexual dimorphism in North American *Entomobrya atrocincta*. However, species diagnosis remains unclear. Chaetotaxy outlined for both *Entomobrya atrocincta* and *Entomobrya nigrocincta* by Ramel et al. (2008) and Jordana (2012) do not match the specimens examined in this study (Table 4). The excessive intraspecific variation in chaetotaxy observed in these specimens raises concern about basing species diagnosis strictly on discrete chaetotaxic characters. Further molecular analysis of European populations is needed in order to elucidate the correct taxonomic status and distribution of these two species.

**Table 4.** Comparison between descriptions of *Entomobrya atrocincta* (and *Entomobrya nigrocincta*) provided by this study with those provided by Jordana (2012), Ramel et al. (2008), and Christiansen and Bellinger (1998). Characters represent the number of dorsal macrosetae present in fields (H1-A10) outlined by Jordana and Baquero (2005) and Jordana (2012). Clear differences between descriptions are highlighted in bold and underlined. Parentheses indicate a rarely observed state. Question marks indicate the characters were not included in the description.

Species description	Sexual dimorphism	H1	H2	H3	H4	H5	T1	T2	A1	A2	A3	A4	A5	A6–A10
*Entomobrya atrocincta*^1^	Yes	3	2(1)	0	3	2	2	3	2(1)	2(3)	1	2	1	8-11
*Entomobrya nigrocincta*^2^	Yes	3	1	0	**2**	2	2	3	1	2	1	**1**	1	9(8?)
*Entomobrya atrocincta*^3^	**No**	**2**	1	0	3	2(3)	2	3	2	3	1	2	1	**13**
*Entomobrya atrocincta*^4^	**No**	?	?	?	?	?	?	?	2	2	1	2	1	?

1 Description of *Entomobrya atrocincta* specimens observed in the present study.

2 Description of *Entomobrya nigrocincta* provided by Jordana (2012) and Ramel et al. (2008).

3 Description of *Entomobrya atrocincta* provided by Jordana (2012).

4 Description of *Entomobrya atrocincta* provided by Christiansen and Bellinger (1998).

The original description of *Entomobrya atrocincta* by Schӧtt (1896) was based on the male form collected from California. Christiansen’s (1958b) descriptions and methods for species delimitation placed heavy emphasis on the male genital plate, which may have led to his inadvertent omission of the female form. Given that the combination *Entomobrya atrocincta* Schӧtt, 1896 has priority over *Entomobrya nigrocincta* Denis, 1923, and also because the holotype designated for *Entomobrya atrocincta* was collected in North America, the specimens along with the descriptions outlined in this study have been assigned to *Entomobrya atrocincta*.

###### Distribution.

North America, Hawaii and possibly Europe. Records of *Entomobrya multifasciata* in North America and Hawaii (Christiansen and Bellinger 1992) are suspect due to their similarity to the female *Entomobrya atrocincta* color form. A considerable number of collections of *Entomobrya atrocincta* from Hawaii also include *Entomobrya multifasciata*, indicating the species is also sexually dimorphic in the Pacific Islands (Christiansen and Bellinger 1992). Palearctic records of *Entomobrya atrocincta* may be *Entomobrya nigrocincta* provided by Jordana (2012). The distribution of *Entomobrya atrocincta* in North America is shown in Suppl. material 2: B.

###### Material examined.

USA: 1♂ in vial, Alabama, Clay Co., Talladega National Forest, on CR6000-1 off of Hwy148 (33.19723,-86.06325), in moss on forest floor, 2.i.2012 (A. Katz & M. DuBray), AK12-5; 6♂ & 2♀ in vial, Illinois, Champaign Co., Champaign, Kaufman Lake Park (40.11514,-88.29000), in bird nest, 8.v.2011, AK11-26; 2♂ on slides, 4♂ & 6♀ in vial, Illinois, Champaign Co., Champaign, Kaufman Lake Park (40.11514,-88.29000), squirrel nest, 8.v.2011, AK11-27; 7♂ & 7♀ on slides, 50+♂ & 50+♀ in vial, Illinois, Champaign Co., Urbana, Natural Resource Building (40.10071,-88.22812), in leaf litter under bush by parking lot, 22.iv.2011, AK11-17; 1♂ on slides, Illinois, Champaign Co., Urbana, Meadowbrook Park (40.08063,-88.20828), in leaf litter, 14.iv.2012, AK12-24; 3♀ in vial, Illinois, Champaign Co., Urbana, South side of Natural Resource Building (40.10110,-88.22963), in sweet gum seed pods and leaf litter, 23.iv.2011 (A. Katz & M. DuBray), AK11-15; 3♀ on slides, 20♂ & 10♀ in vial, Illinois, Champaign Co., Urbana, Natural Resource Building (40.10047,-88.22840), in leaf litter under bush along Pennsylvania Ave, 5.v.2011, AK11-25; 1♂ & 1♀ on slides, 50+♂ & 50+♀ in vial, Tennessee, Knox Co., University of Tennessee, Ag. Campus, Morgan Hall (36.01023,-83.93829), on moist fallen bark, April 2010 (E. C. Bernard); 1♂ & 1♀ in vial, Tennessee, Knox Co., Farragut, 12108 Ridgeland Drive (36.01023,-83.93829), in old bald-faced hornet nest, 2.iii.2008 (E. C. Bernard).

##### Entomobrya bicolor

Taxon classificationAnimaliaCollembolaEntomobryidae

Guthrie, 1903

**Figure 11.**
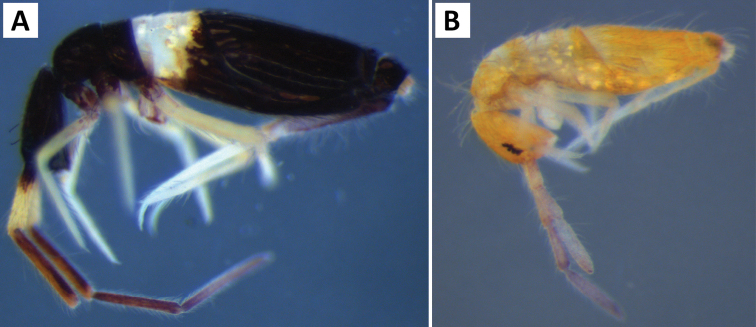
Color patterns of *Entomobrya bicolor*: **A** adult **B** young immature instar. Both photographed specimens were collected from Henderson Co., IL.

**Figure 12.**
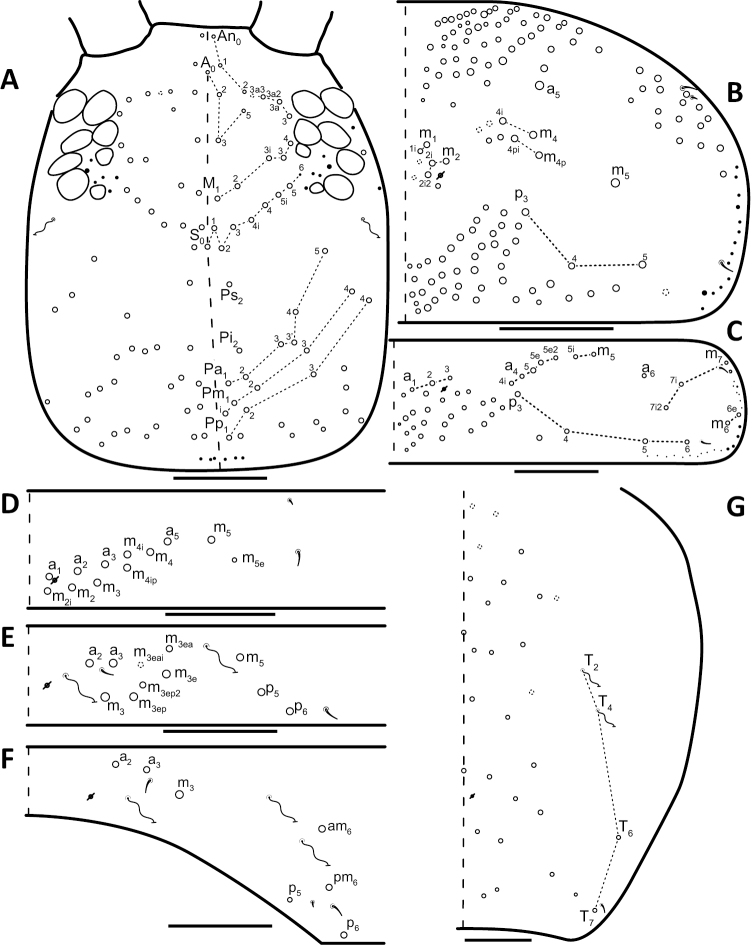
Dorsal chaetotaxy of *Entomobrya bicolor*: **A** Head **B** Mesothorax **C** Metathorax **D** 1^st^ abdominal segment **E** 2^nd^ abdominal segment **F** 3^rd^ abdominal segment **G** 4^th^ abdominal segment. Scale bars = 100 µm. See Figure 5 for symbol legend.

**Figure 13.**
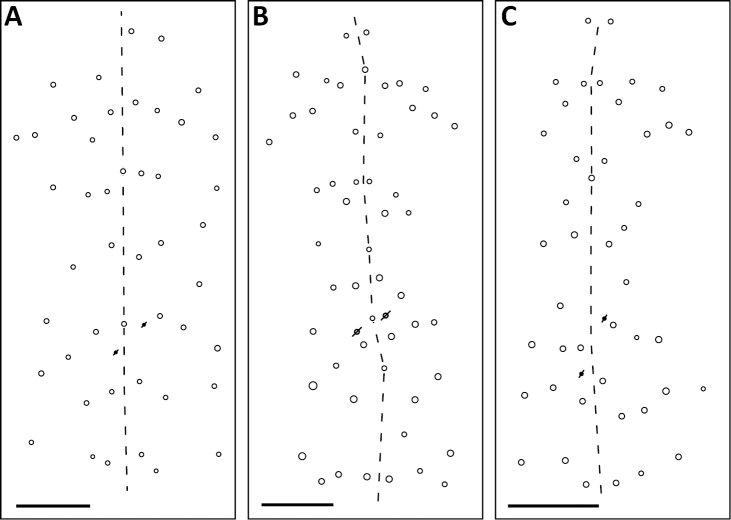
Macrosetae variation and asymmetrical polymorphism of Abd. 4 in the *Entomobrya bicolor* complex: **A** *Entomobrya bicolor* **B** *Entomobrya decemfasciata* **C** *Entomobrya quadrilineata*. Only medial macrosetae (those which occur between bothriotricha T_2_ and T_4_) are included in diagram. The dotted line represents the medial division of Abd. 4. Scale bars = 100 µm.

Figs 2 , 11 , 12 , 13A , 39

###### Description.

*Body shape and color pattern.* Body elongate and cylindrical. Only one reported color form in adults (Fig. 11A), dark brown or black pigment covering whole body except for a white band across Abd. 1, 2 and medial area of Abd. 3; Abd. 5 sometimes with 1+1 pale spots; legs, furcula, Abd. 6 pale, lacking all dark pigmentation; antennae lightly colored with brown or purple pigment, usually with a white area on distal half of 1^st^ antennal segment. Juvenile pattern distinct from adult, yellow background without dark pigment except for eye patch; legs, furcula, Abd. 6 pale, lacking all dark pigmentation; antennae with light purple pigment. (Fig. 11B)

*Head*. Apical bulb of 4^th^ antennal segment usually bilobed, rarely simple. Long differentiated smooth setae on ventral side of 1^st ^antennal segment ≈ 3–4× short seta. Prelabral setae ciliate. Ornamentation of the distal margin of the labral papillae with a single seta or spine-like projection (Fig. 2). Labial papilla E with lateral appendage reaching just above tip of papilla. Labial triangle in one individual with 2 small supplementary ciliate microsetae internal to M1. Dorsal head chaetotaxy as in Figure 12A: macrosetae An’_0_, A_6_, S’_0_, S_6_, Ps_3_, Ps_5_, Pi_1_, Pi_3_, and Pm_1i_ always absent; An_3a3_ usually absent (present on one side in one specimen). Eyes G and H small and subequal. Eye patch with 5 setae.

*Thorax*. Thoracic chaetotaxy abundantly developed, highly variable with many supplemental macrosetae. Th. 2 macrosetae p_6e_ and p_6ep_ absent and macrosetae m_4i2_, m_4i3_ polymorphic (Fig. 12B). Th. 3 macrosetae a_5e3_, m_4_, m_5p_, a_6i_, a_7_, and m_7_ are absent (Fig. 12C). If present, m_7_ is always a mesoseta. Chaetotaxy of zone Pm of both thoracic segments densely packed with many supplemental macrosetae, forming wing-like patches of posterior setae extending near anterior row, typical for species within the *Entomobrya bicolor* complex (Fig. 3D). Position of pseudopores on Th. 3 atypical for *Entomobrya*, displaced anteriorly and closer to macrosetae a_1_, a_2_, and a_3_ than in other species.

*Legs.* Trochanteral organ with rectangular setal pattern and up to 37 setae. Unguis with 4 internal teeth; basal teeth enlarged and located approximately middle of inner claw length.

*Abdomen*. Abdominal chaetotaxy highly developed. Row of microsetae along entire posterior margin present in all segments (not displayed in figure). Abd. 1 with 12 macrosetae (Fig. 12D). Abd. 2 with 10-11 macrosetae: a_2_, a_3_, m_3_, m_3e_, m_3ep_, m_3ep2_, m_3ea_, m_5_, p_5_, and p_6_ always present. Abd. 2 macroseta m_3eai_ sometimes present, m_3ei_ always absent (Fig. 12E). Abd. 3 macrosetae a_2_, a_3_, m_3_, am_6_, pm_6_, p_5_, p_6_ present; a_1_ always absent (Fig. 12F). Abd. 4 elongated, with at least 22 inner macrosetae (Fig. 12G), number of macrosetae extremely variable between individuals and within individuals (Fig. 13A). Position of pseudopores on Abd. 4 unstable, even varying with respect to macroseta and bothriotricha between sides on same individual (Fig. 13A). Mucronal teeth subequal; mucronal spine enlarged.

###### Remarks.

*Entomobrya bicolor* adults can be easily distinguished by the unique color pattern described above combined with the absence of head macroseta Ps_5_ (see Table 2 for additional diagnostic characters). Adults have a single, highly stable color form that is easily recognizable and perfectly acceptable for diagnosis (Fig. 11A). Molecular data from Katz et al. (2015) revealed that a small specimen found with *Entomobrya bicolor*, which was completely yellow and lacking all dark pigment, is the juvenile form of *Entomobrya bicolor* (Fig. 11B). The *Entomobrya bicolor* juvenile color form has never been reported in the literature. Unfortunately, it may be difficult to diagnose juvenile members of this species if collected without adults present in the sample.

Christiansen (1958b) placed this species in what he termed “the *Entomobrya bicolor* group”; a complex of three closely related species; *Entomobrya quadrilineata*, *Entomobrya decemfasciata*, and *Entomobrya bicolor*. This group is characterized by a highly elongate, cylindrical body, a relatively long furcula, antennae and legs, and by their abundant and highly variable chaetotaxy; many duplicate, supplementary macrosetae, distinctive and augmented setal patterns in thoracic zone Pm, expanded chaetotaxy of Abd. 4, and high levels of asymmetry (Fig. 13A). The extreme setal variation obscures potentially informative characters and results in a lack of discrete, useful diagnostic chaetotaxy between species in this complex. The absence of head macroseta Ps_5_ is the only character (other than color pattern) that differentiates *Entomobrya bicolor* from *Entomobrya decemfasciata* and *Entomobrya quadrilineata*. Color pattern is critical for species diagnosis within this complex. See Table 5 for a summary of the important diagnostic characters to separate species in this complex.

**Table 5.** Important diagnostic characters for the separation of species within the bicolor complex: *Entomobrya bicolor*, *Entomobrya decemfasciata*, and *Entomobrya quadrilineata*. Character states: absent (0), present (1).

Species	Head macrosetae Ps_5_	Parallel, longitudinal bands on thorax
*Entomobrya bicolor*	0	0
*Entomobrya decemfasciata*	1	0
*Entomobrya quadrilineata*	1	1

###### Distribution.

Endemic to North America (Suppl. material 2: C).

###### Material examined.

USA: 9 on slides, 6 in vial, Illinois, Henderson Co., Big River State Forest (41.03435,-90.91620), vacuum sand prairie, 8.vi.2011 (C. H. Dietrich).

##### Entomobrya citrensis

Taxon classificationAnimaliaCollembolaEntomobryidae

Katz & Soto-Adames sp. n.

http://zoobank.org/4C33A32D-EFF4-4B53-8880-2C9C3844076C

**Figure 14.**
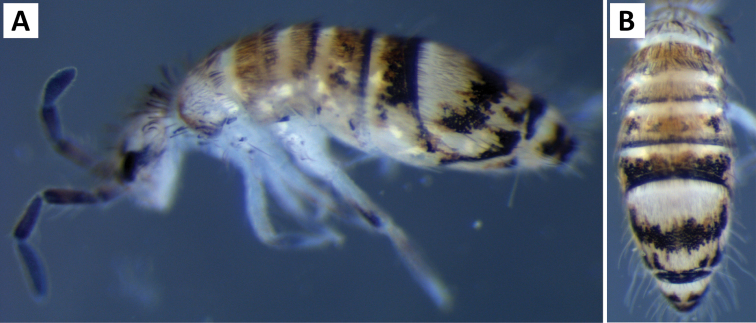
Color pattern of *Entomobrya citrensis* sp. n. Lateral (**A**) and dorsal (**B**) views of specimen collected from Citrus Co., FL.

**Figure 15.**
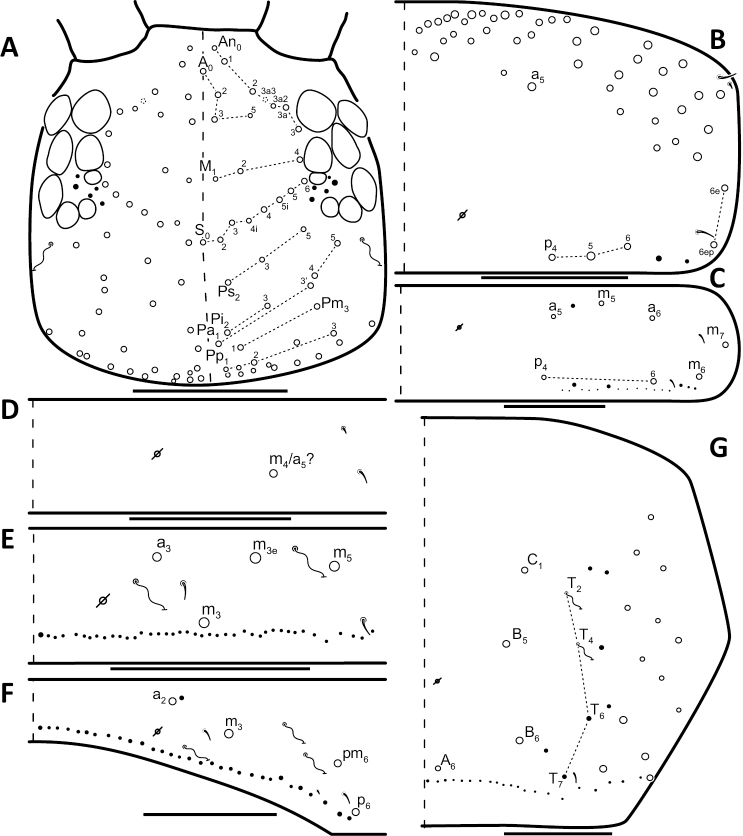
Dorsal chaetotaxy of *Entomobrya citrensis* sp. n.: **A** Head **B** Mesothorax **C** Metathorax **D** 1^st^ abdominal segment **E** 2^nd^ abdominal segment **F** 3^rd^ abdominal segment **G** 4^th^ abdominal segment. Scale bars = 100 µm. See Figure 5 for symbol legend.

**Figure 16.**
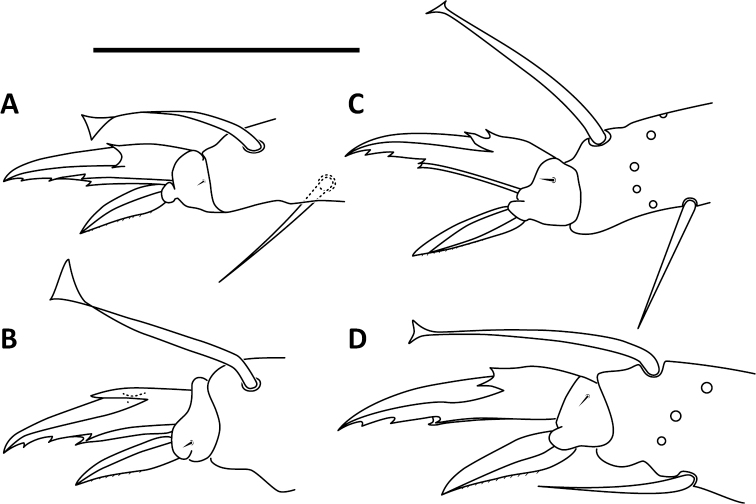
Unguis (top claw) and unguiculus (bottom claw) on 3^rd^ pair of legs. **A** *Entomobrya citrensis* sp. n. **B** *Entomobrya jubata* sp. n. **C** *Entomobrya neotenica* sp. n. **D** *Entomobrya unifasciata* sp. n.

Figs 2 , 14 , 15 , 16A

###### Etymology.

This species is named after the locality it was collected in: Citrus County, Florida. Citrensis is Latin for “from the place of citrus”.

###### Type material.

*Holotype*, ♂, USA: Florida, Citrus County, Chassahowitzka National Wildlife Refuge (28.75997,-82.57583), beating vegetation, 12.viii.2011 (A. Katz & J. Cech), AK11-134.

*Paratypes*, USA: 1 on slide, Florida, Citrus Co., Chassahowitzka National Wildlife Refuge (28.75997,-82.57583), under bark, 12.viii.2011 (A. Katz & J. Cech), AK11-136; 1 on slide, Florida, Citrus Co., Chassahowitzka National Wildlife Refuge (28.75997,-82.57583), beating vegetation and in leaf litter, 5.i.2014 (A. Katz & M. DuBray), AK14-1.

###### Description.

*Body shape and color pattern.* Body dorso-ventrally flattened. Length up to 1.4mm. Color pattern monomorphic (Fig. 14): background white, or slightly yellow, with black and traces of dark brown pigmentation forming transversal bands and spots. Thorax dorsal pigmentation patchy and irregular. Abd. 2 usually with 2 dark lateral spots. Posterior margin Abd. 2 and the anterior margin of Abd. 4 with a dark transverse band. Abd. 4 with a conspicuous irregular dark transverse band medially. The antennae are completely pigmented purple.

*Head*. Apical bulb of 4^th^ antennal simple. Long differentiated smooth setae on ventral side of 1^st ^antennal segment ≈2.5× short setae. Prelabral setae with very fine ciliations that look smooth at low magnification under light microscopy. Ornamentation of the distal margin of the labral papillae with 3-4 spine-like projections on the inner papillae and 2 spine-like projections on the external papillae. Lateral appendage of labial papilla E slightly curved, relatively thin, reaching just below tip of papilla. Dorsal head chaetotaxy slightly reduced (Fig. 15A): macrosetae An’_0_, A_6_, M_3_, M_3i_, S’_0_, S_1_, Pi_1_, Pa_2_, Pa_3_, Pm_2_, and Pm_1i_ always absent; An_3a3_ present, asymmetrically absent in one specimen. Eyes G and H small and subequal. Eye patch with 5 setae.

*Thorax*. Thoracic chaetotaxy reduced and fixed, no macrosetae variation observed. Row of microsetae along entire posterior margin of Th. 2 and Th. 3 (not displayed in figures). Th. 2, with a_5_ and posterior macrosetae p_4_, p_5_, p_6_, p_6e_, and p_6ep_ (Fig. 15B). Th. 3, with 7 macrosetae present: a_5_, a_6_, m_5_, m_6_, m_7_, p_4_, and p_6_; macrosetae a_1_, a_2_, and a_3_ absent (Fig. 15C). Zone Pm (Fig. 3) without macrosetae.

*Legs*. Trochanteral organ with triangular setal pattern and up to 14 setae. Unguis with 4 internal teeth; basal teeth located approximately middle of inner claw length (Fig. 16A).

*Abdomen*. Abdominal chaetotaxy reduced and stable; no macrosetae variation observed. Abd. 1 with 1 macroseta only (Fig. 15D). Abd. 1 row of microsetae along posterior margin is present (not displayed in figure). Abd. 2 with 4 macrosetae: a_3_, m_3_, m_3e_, and m_5_ (Fig. 15E). Abd. 3 with 4 macrosetae: a_2_, m_3_, pm_6_, and p_6_; mesosetae a_2a_ sometimes present, inserted slightly anterior and exterior to a_2_; socket of a_2a_ large, resembling that of macroseta a_3_, but its close proximity to a_2 _suggests a duplication of a_2_ rather than homologous to a_3_ (Fig. 15F). Abd. 4 with 4 inner macrosetae (Fig. 15G). Mucronal teeth subequal.

###### Remarks.

*Entomobrya citrensis* sp. n. can be distinguished by its color pattern in combination with the absence of Th. 2 macrosetae m_2_ and m_5_, Abd. 2 a_2_ and m_3ep_, and the presence of Abd. 2 a_3_ (see Table 2 for additional diagnostic characters).

This species is closely related to *Entomobrya assuta*; both have highly compressed, or dorso-ventrally flattened bodies, reduced chaetotaxy, and similar color patterns. Head macroseta Ps_3_ and Abd. 2 macroseta a_3_ are both present in *Entomobrya citrensis* sp. n., but are absent in *Entomobrya assuta*. These species can also be separated by color pattern and morphology of the labral papillae (Fig. 2).

See remarks for *Entomobrya assuta* for additional diagnosis information. Only a few specimens of *Entomobrya citrensis* sp. n. were observed from one locality. Additional sampling may reveal more variation in color pattern or chaetotaxy.

###### Distribution.

Endemic to North America. Reported from a single locality: Chassahowitzka National Wildlife Refuge in Citrus County, Florida (Suppl. material 2: D).

##### Entomobrya clitellaria

Taxon classificationAnimaliaCollembolaEntomobryidae

Guthrie, 1903

**Figure 17.**
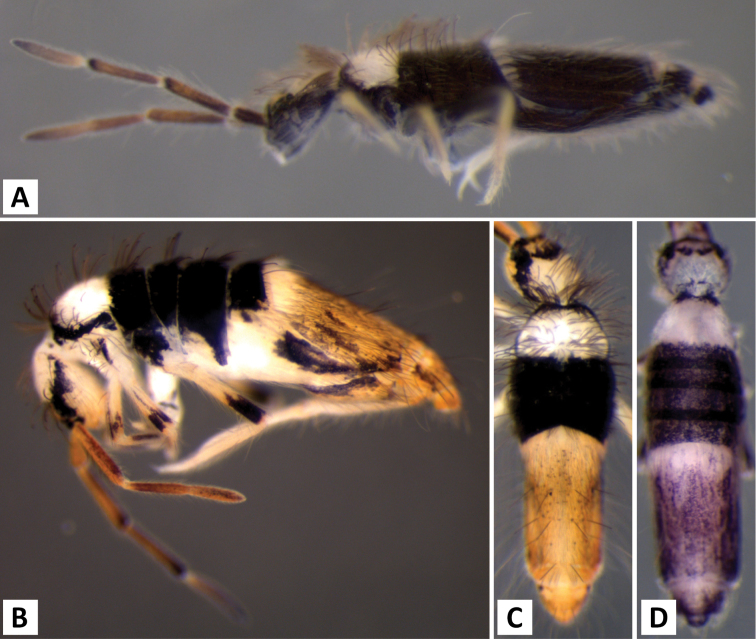
Color patterns of *Entomobrya clitellaria*. Photographed specimens are from the following localities: **A** Monroe Co., IL **B** Jasper Co., IL **C** Kankakee Co., IL **D** Monongalia Co., WV.

**Figure 18.**
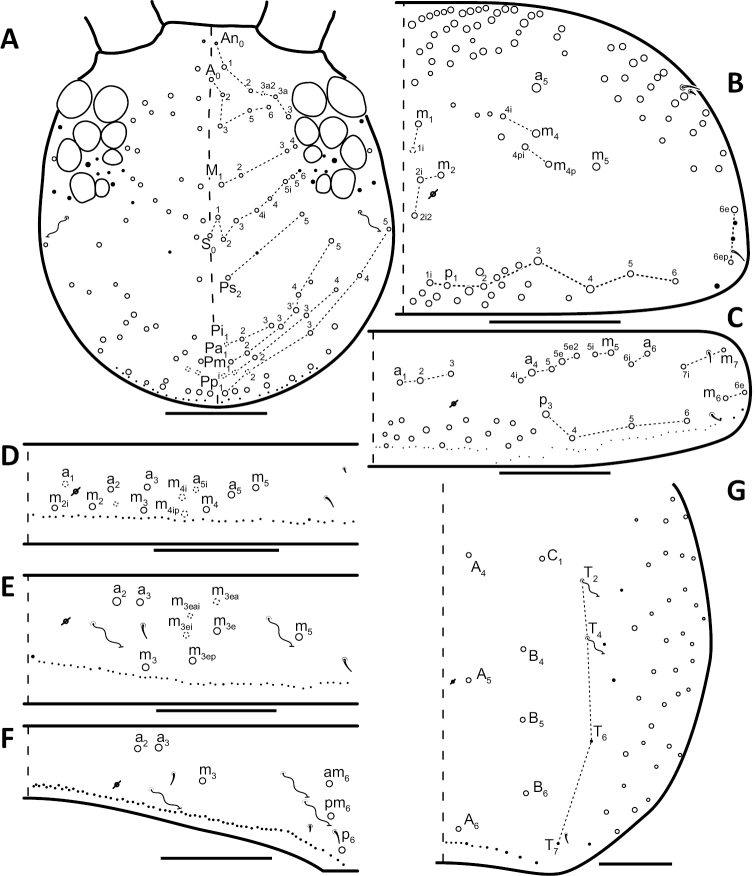
Dorsal chaetotaxy of *Entomobrya clitellaria*: **A** Head **B** Mesothorax **C** Metathorax **D** 1^st^ abdominal segment **E** 2^nd^ abdominal segment **F** 3^rd^ abdominal segment **G** 4^th^ abdominal segment. Scale bars = 100 µm. See Figure 5 for symbol legend.

Figs 2 , 17 , 18 , 39

###### Description.

*Body shape and color pattern*. Body dorso-ventrally flattened. Dimorphic color pattern, unrelated to sex (Fig. 17): dark blue, purple, or black pigment covers Th. 3 and Abd. 1-3. Abd. 4 usually white, yellow, or orange with variable levels of pigment ranging from an irregular medial transverse band, randomly distributed irregular patches, or completely pigmented. Th. 2 always white, with a dark band along the anterior margin; medial area of Th. 2 almost transparent in some specimens and internal fat bodies can be observed through the cuticle under a dissecting microscope. Darker specimens with head mostly covered by dark pigment. Antennae usually entirely covered by purple pigment, but some specimens have a mixture of orange, brown, and purple coloration.

*Head*. Apical bulb of 4^th^ antennal segment usually simple, sometimes bilobed. Long differentiated smooth setae on ventral side of 1^st^ antennal segment ≈3× short setae. Prelabral setae ciliate. Ornamentation of the distal margin of the labral papillae with a single seta or spine-like projection (Fig. 2). Lateral appendage of labial papilla E slightly curved, relatively thin, nearly reaching tip of papilla. Labial triangle in one individual with 1 small supplementary ciliate microsetae internal to M1. Dorsal head chaetotaxy (Fig. 18A) with macrosetae An’_0_, A_3a3_, M_3i_, S’_0_, S_6_, and Ps_3_ always absent; An_3a2_ usually present; Pi_1_, Pm_1_, Pm_1i_, and Pp_2_ present or absent. Eyes G and H small and subequal. Eye patch with 5 setae.

*Thorax*. Thoracic chaetotaxywell-developed, with some slight variation. Th. 2 macrosetae m_4i3_ absent, m_1i_ usually present (Fig. 18B). Th. 3 macrosetae a_5e3_, m_4_, m_5p_, and a_7 _absent (Fig. 18C). Both thoracic segments with many supplemental macrosetae in zone Pm (18B,C).

*Legs*. Trochanteral organ with triangular setal pattern and up to 26 setae. Unguis with 4 inner teeth; basal teeth located approximately middle of inner claw length.

*Abdomen*. Abdominal chaetotaxy highly developed. Abd. 1 with 8-13 macrosetae (Fig. 18D). Abd. 2 macroseta a_2_, a_3_, m_3_, m_3ep_, m_3e_, and m_5_ present; M_3ei_ and M_3ea _usually present and M_3eai_ usually absent (Fig. 18E). Abd. 3 macrosetae a_2_, a_3_, m_3_, am_6_, pm_6_, and p_6_ present; a_1_ always absent (Fig. 18F). Abd. 4 chaetotaxy stable, with 7 macrosetae between bothriotrichal complexes (Fig. 18G). Mucronal teeth subequal.

###### Remarks.

*Entomobrya clitellaria* can be distinguished by the combination of color pattern, absence of macroseta head S’_0_ and Abd. 3 a_1_ and presence of head Ps_5_, Th. 2 m_5_, Abd. 2 m_3ep_, and Abd. 3 a_2 _(see Table 2 for additional diagnostic characters). This species has a relatively conspicuous and diagnostic color pattern, but may be confused with the male *Entomobrya atrocincta*. However, there are obvious differences in their morphology outlined in Table 3. Christiansen and Bellinger (1998) described six different color forms, but after extensive examination of material collected for this study, it was determined that many (but not all) of the color forms they described were most likely variants within a continuous gradient of color pattern variation, without clear genetic isolation (Katz et al. 2015). The two lighter forms (labeled E and F in Christiansen and Bellinger 1998) proved to be elusive and were not collected during this study.

This species is closely related to *Entomobrya jubata* sp. n., but can be easily separated by color pattern, chaetotaxy, and morphology of the labral papillae. The absence of head macrosetae S’_0_ and the presence of head macrosetae Ps_5_ separate *Entomobrya clitellaria* from *Entomobrya jubata* sp. n. Labral papillae morphology also differs between these species: *Entomobrya jubata* sp. n. has two or three seta or spine-like projection on each papilla, while *Entomobrya clitellaria* only has one seta or spine-like projection per papilla (Fig. 2).

###### Distribution.

Endemic to North America (Suppl. material 2: E).

###### Material examined.

USA: 1 on slide, Florida, Taylor Co., Econfina State Park (30.0656,-83.91066), under bark, 9.viii.2011, AK11-116; 1 in vial, Illinois, Champaign Co., Urbana, Brownfield Woods (40.14462,-88.16543), on bushes and low-lying shrubs, 7.vii.2011, AK11-60; 1 on slide, Illinois, Champaign Co., Urbana, Brownfield Woods (40.14391,-88.16468), under bark, 10.ix.2009 (F. Soto-Adames); 1 in vial, Illinois, Coles Co., Fox Ridge State Park (39.40248,-88.14893), under bark, 15.vii.2011 (A. Katz & F. Soto-Adames), AK11-81; 3 on slides, 20 in vials, Illinois, Jasper Co., Sam Parr State Fish and Wildlife Area (39.03275,-88.12474), under bark, 15.vii.2011 (A. Katz & F. Soto-Adames), AK11-73, AK11-74 & AK11-75; 1 in vial, Illinois, Jasper Co., Sam Parr State Fish and Wildlife Area (39.03275,-88.12474), in leaf litter, 15.vii.2011 (A. Katz & F. Soto-Adames), AK11-77; 1 on slide, 1 in vial, Illinois, Jo Davies Co., Princess Mine 1 (42.30565,-90.39740), under bark, 26.viii.2011, AK11-142; 1 in vial, Illinois, Jo Davies Co., S Blanding Rd, Stevenson Property (42.29895,-90.36967), under bark, 27.viii.2011, AK11-148; 1 on slide, Illinois, Kankakee Co., Kankakee River State Park (41.19482,-87.96875), from bird nest, 10.iv.2011, AK11-6; 4 on slides, Illinois, Monroe Co., Bat Sump Cave, Berlese of bark, 1-3.xi.2009 (S. Taylor & F. Soto-Adames), sjt09-130; 4 on slides, 50+ in vial, Illinois, Piatt Co., Lodge County Forest Preserve Park (40.06709,-88.56596), dead logs, cavity in tree, and under bark, 23.vii.2011, AK11-100; 2 on slides, 10 in vial, Illinois, Pope Co., Bell Smith Springs (37.51927,-88.65738), leaf litter, 24.ix.2011, AK11-166; 2 on slides, Illinois, Vermilion Co., Kickapoo State Park (40.16576,-87.74746), on rotten log, 30.xi.1989 (F. Soto-Adames); 1 in vial, Illinois, Vermilion Co., Kennekuk Cover County Park, Windfall Prairie Nature Preserve (40.20995,-87.74181), aspirated from bushes, 16.vi.2011 (A. Katz & F. Soto-Adames), AK11-59; 1 in vial, Michigan, Ingham Co., Michigan State University, Baker Wdlt. (42.66527,-84.36264) under bark of standing dead pine, 24.vii.2008 (E. C. Bernard), BW-11; 1 on slide, 3in vial, Michigan, St. Clair Co., Algonac State Park (42.65447,-82.52430), under bark of recently fallen maple, 25.vii.2008 (E. C. Bernard), ASP-15; 1 on slide, West Virginia, Monongalia Co., Tyrone (39.62746,-79.86567), under collected on the lower branches of eastern hemlock, 19.vii.2005 (R. M. Turcotte), ID#44-GE-21-5-4-A.

##### Entomobrya decemfasciata

Taxon classificationAnimaliaCollembolaEntomobryidae

(Packard), 1873

**Figure 19.**
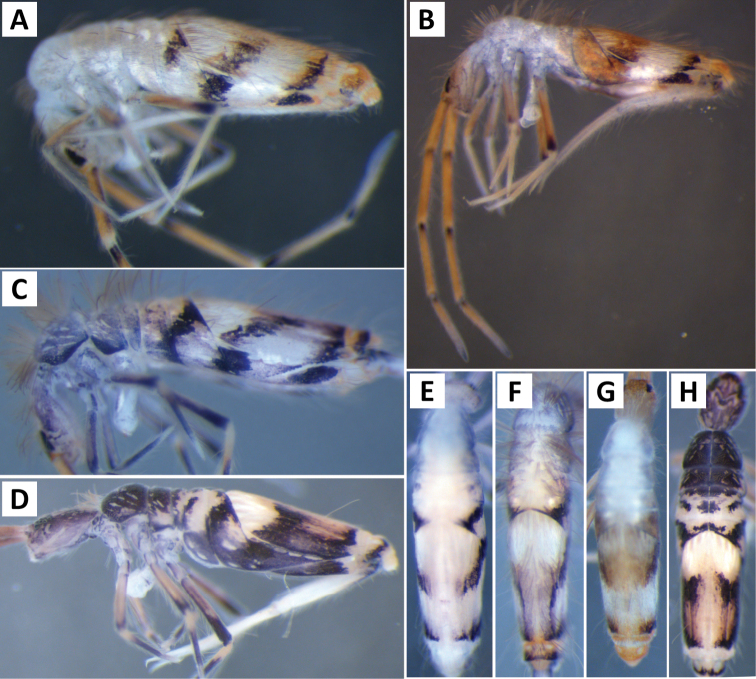
Color patterns of *Entomobrya decemfasciata*. Photographed specimens are from the following localities: **A** Stewart Co., TN **B** Stewart Co., TN **C** Clay Co., AL **D** Sevier Co., TN **E** Sequatchie Co., TN **F** Clay Co., AL **G** Union Co., IL **H** Sevier Co., TN.

**Figure 20.**
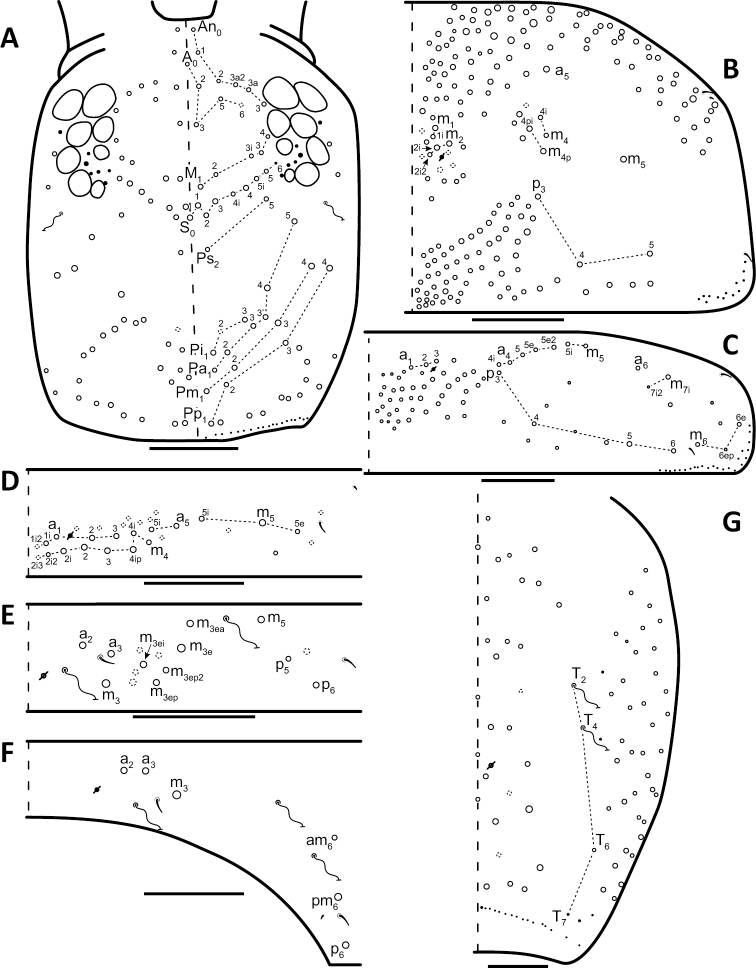
Dorsal chaetotaxy of *Entomobrya decemfasciata*: **A** Head **B** Mesothorax **C** Metathorax **D** 1^st^ abdominal segment **E** 2^nd^ abdominal segment **F** 3^rd^ abdominal segment **G** 4^th^ abdominal segment. Scale bars = 100 µm. See Figure 5 for symbol legend.

Figs 2 , 3D , 13B , 19 , 20 , 39

###### Description.

*Body shape and color pattern*. Body very elongate and cylindrical with mesothorax forming a slight hump behind head. Color pattern remarkably variable with continuous variation and many intermediate forms (Fig. 19). Typical pattern without thoracic bands but with 2-4 irregular, angled bands on lateral margins of abdomen. Color also variable ranging from white, yellow, orange, or sometimes light blue or purple background with black, dark blue, or brown pigment forming bands. Dark bands outlining posterior and lateral margins of Th. 2 and Th. 3 sometimes present. Apex of femora usually with a dark patch. Head either entirely blue or purple, or lacking all pigment except for the eye patches. Dark patches of pigment usually occur on distal end of antennal segments 2-4. Juveniles usually with light blue pigment background and faint brown abdominal banding.

*Head*. Apical bulb of 4^th^ antennal segment usually bilobed or simple, but with up to 6 distinct lobes. Long differentiated smooth setae on ventral side of 1^st ^antennal segment ≈3× short setae. Ornamentation of the distal margin of the labral papillae with single seta or spine-like projection (Fig. 2). Lateral appendage of labial papilla E slightly curved, almost twice as long as papilla. Labial triangle chaetotaxy slightly irregular and atypical for this genus: M1, r, E, L1, L2 ciliate; r significantly smaller than other setae; A1-A5 smooth; sometimes 2 additional ciliate setae inserted internal to M1 and A1, respectively, often relatively difficult to observe. Post labial setae abundant, all type 5. Dorsal head macrosetae (Fig. 20A) An’_0_, An_3a3_, S’_0_, S_6_, Ps_3_, and Pm_1i_ absent; A_6_ sometimes present. Eyes G and H small and subequal. Eye patch with 5 setae.

*Thorax*. Thoracic chaetotaxy developed, highly variable, with many supplemental macrosetae. Th. 2 macrosetae p_6_, p_6e_ and p_6ep_ absent. Macrosetae in Zone A so abundant that usually merge with medial macrosetae, forming a single, large patch of setae (Figs 3D; 20B). Th. 3 macrosetae a_5e3_, m_4_, m_5p_, a_6i_, a_7_, and m_7_ absent (Fig. 20C). Macrosetae in Zone Pm also very abundant on both thoracic segments, with many supplemental macrosetae present forming wing-like patches of posterior setae extending near the anterior row (Fig. 3D). Insertion of pseudopore on Th. 3 different from most *Entomobrya*, displaced anteriorly, very close to macrosetae a_1_, a_2_, and a_3_. Additional duplicate or supplementary setae often form columns internal to a_1_.

*Legs*. Trochanteral organ with rectangular setal pattern and up to 86 setae. Unguis with 4 internal teeth; basal teeth located approximately middle of inner claw length.

*Abdomen*. Abdominal chaetotaxy extensively developed. Row of microsetae along entire posterior margin present in all segments (not displayed in figure). Abd. 1 with 16-30 macrosetae (Fig. 20D). Abd. 2 macroseta M_3ei_ always present, with up to 4 additional supplementary macrosetae (possibly including M_3eai_) internal to m_3e_; macrosetae a_2_, a_3_, m_3_, m_3e_, m_3ep_, m_3ep2_, m_3ea_, m_5_, p_5_, and p_6_ present (Fig. 20E). Abd. 3 with macrosetae a_2_, a_3_, m_3_, am_6_, pm_6_, and p_6_; a_1_ absent (Fig. 20F). Abd. 4 very elongated, with at least 25 inner macrosetae (Fig. 20G), but number of macrosetae extremely variable between individuals and even within a single individual (Fig. 13B). Insertion of pseudopores on Abd. 4 varying with respect to macroseta and bothriotricha even in same individual (Fig. 13B). Basal mucronal tooth enlarged.

###### Remarks.

*Entomobrya decemfasciata* can be distinguished by the absence of parallel, longitudinal bands on the thorax, the presence of 2-4 irregular, angled bands on lateral margins of abdomen, and the presence of head macroseta Ps_5_ (see Table 2 for additional diagnostic characters). This species belongs to the *Entomobrya bicolor* group (see remarks for *Entomobrya bicolor*), and exhibits high levels of variation in both color pattern and chaetotaxy. *Entomobrya decemfasciata* is perhaps the most setaceous species of *Entomobrya* reported for North America, clothed in hundreds of macrosetae. This abundant and hyper-variable chaetotaxy provides few characters to differentiate *Entomobrya decemfasciata* from *Entomobrya bicolor* and *Entomobrya quadrilineata*. However, clear differences in color pattern can be observed between these species; *Entomobrya bicolor* lacks band or stripes, *Entomobrya quadrilineata* always has two parallel longitudinal stripes extending from the thorax through Abd. 2 and *Entomobrya decemfasciata* never has bands or stripes on the thorax. Table 5 details important diagnostic characters to separate species within this species complex.

The considerable variation in chaetotaxy and color pattern and relatively high molecular divergences between *Entomobrya decemfasciata* color forms suggest the presence of a cryptic species complex (Katz et al. 2015). However, the lack of diagnostic morphological characters between color forms does not allow the circumscription of new species at this time. More specimens and additional molecular and morphological analyses are needed for further action.

This species has a long history of taxonomic issues (Christiansen 1958b) and its separation from *Entomobrya quadrilineata* only became evident after a thorough molecular analysis (Katz et al. 2015). Christiansen (1958b) separated *Entomobrya decemfasciata* from *Entomobrya quadrilineata* using color pattern, male genital plate, antennal ratios, and later added chaetotaxy in *The Collembola of North America* (Christiansen and Bellinger 1998). High variability in color pattern and chaetotaxy, deformation of antennae after slide mounting, and the difficulty of discerning the morphology of setae on the genital plate, lessens the utility of these characters for diagnosis. Christiansen and Bellinger (1998) even suggested *Entomobrya quadrilineata* may be a variant form of *Entomobrya decemfasciata* and seemed uncertain regarding differences in chaetotaxy. Furthermore, Christiansen (1958b) reported three distinct color forms for *Entomobrya decemfasciata* (all of which had a V-shape or two angled lateral bands on Abd. 2 and lacking thoracic stripes) and three distinct color forms for *Entomobrya quadrilineata*, one of which also lacks thoracic stripes. During the course of this study, many color forms were collected, including forms both with and without thoracic stripes occurring together in the same sample. These sympatric forms lacked a V-shape or two lateral angled bands on the 2^rd^ abdominal segment, and, following Christiansen and Bellinger’s (1998) concept, were diagnosed as *Entomobrya quadrilineata*. However, large molecular distances made it apparent that forms with and without thoracic stripes were separate species (Katz et al. 2015), warranting further diagnostic inquiry. Type specimens from the Illinois Natural History Survey were attained for both *Entomobrya quadrilineata* and *Entomobrya decemfasciata*. Though both types, preserved in alcohol, were in relatively poor condition, two longitudinal thoracic stripes were clearly observed on the *Entomobrya quadrilineata* specimen (Suppl. material 3: A, B). Thoracic banding was not observed on the type specimen of *Entomobrya decemfasciata* (Suppl. material 3: C). Based on molecular evidence (Katz et al. 2015) and the observations of types, specimens with the morphology corresponding to both *Entomobrya quadrilineata* and *Entomobrya decemfasciata* can be diagnosed by the presence or absence of parallel thoracic longitudinal stripes. Christiansen’s (1958b) report of an *Entomobrya quadrilineata* color form lacking thoracic stripes may have been a case of misidentification of *Entomobrya decemfasciata*. If individuals with and without longitudinal bands were collected together, similar morphology and abdominal pigmentation may have led him to conclude that they were both *Entomobrya quadrilineata*. However, we now know that both species can occur in sympatry.

###### Distribution.

Endemic to North America (Suppl. material 2: F).

###### Material examined.

USA: “*Type material*”, 1 on slide, Knoxville, Tennessee (Dr. Curtis), INHS Cat. No. 528,321; *Cotype*, 1 on slide, no locality information; 1 on slide, 17 in vial, Alabama, Clay Co., Talladega National Forest, CR-7 & Hwy148 (33.19723,-86.06325), leaf litter, 2.i.2012, AK12-2; 3 on slides, 20 in vial, Illinois, Union Co., Anna, Shawnee National Forest, Rich’s Cave, vestibule of back high entrance, moist leaf litter along the wall and some dry litter from the center of the vestibule, 21.vi.2012 (F. Soto-Adames, S. Taylor & A. Katz); 16 in vial, Illinois, Union Co., Anna, Shawnee National Forest, Rich’s Cave, humid and cool litter in side niche near main entrance, 21.vi.2012 (F. Soto-Adames, S. Taylor & A. Katz); 4 on slides, 2 in vial, Tennessee, Sequatchie Co., Moonshadow (35.64167,-83.76359), forest leaf litter, 14.i.1997 (M. M. Gibbs); 2 on slides, Tennessee, Sevier Co., Great Smoky Mountains National Park, ATBI plot, Goshen Prong (35.67961,-83.50021), malaise 22 83 32 34 35 36 38, 12.xi.2001-5.xii.2001 (Parker, Stocks & Peterson); 2 on slides, 3 in vial, Tennessee, Stewart Co., Land Between the Lakes National Recreation Area, Fox Ridge Rd (36.66392,-87.98596), leaf litter, 7.viii.2011, AK11-105; 2 on slides, 50+ in vial, Tennessee, Stewart Co., Land Between the Lakes National Recreation Area, Fox Ridge Rd. (36.66392,-87.98596), leaf litter, 7.viii.2011, AK11-107; 2 on slides, 11 in vial, Tennessee, Stewart Co., Land Between the Lakes National Recreation Area (36.53830,-87.91428), leaf litter, 7.viii.2011, AK11-108.

##### Entomobrya intermedia

Taxon classificationAnimaliaCollembolaEntomobryidae

Brook, 1883

**Figure 21.**
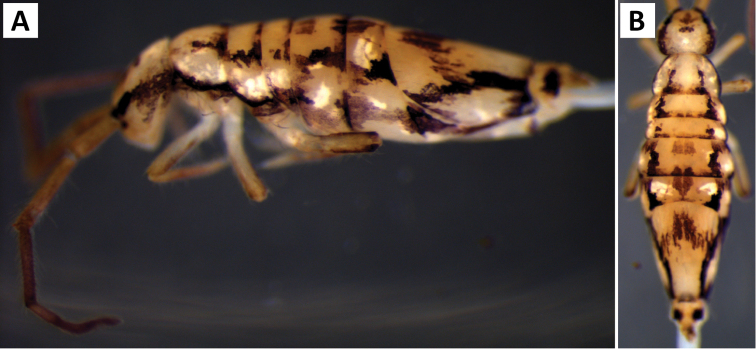
Color pattern of *Entomobrya intermedia*. Lateral (**A**) and dorsal (**B**) views of specimen collected from Chester Co., PA.

**Figure 22.**
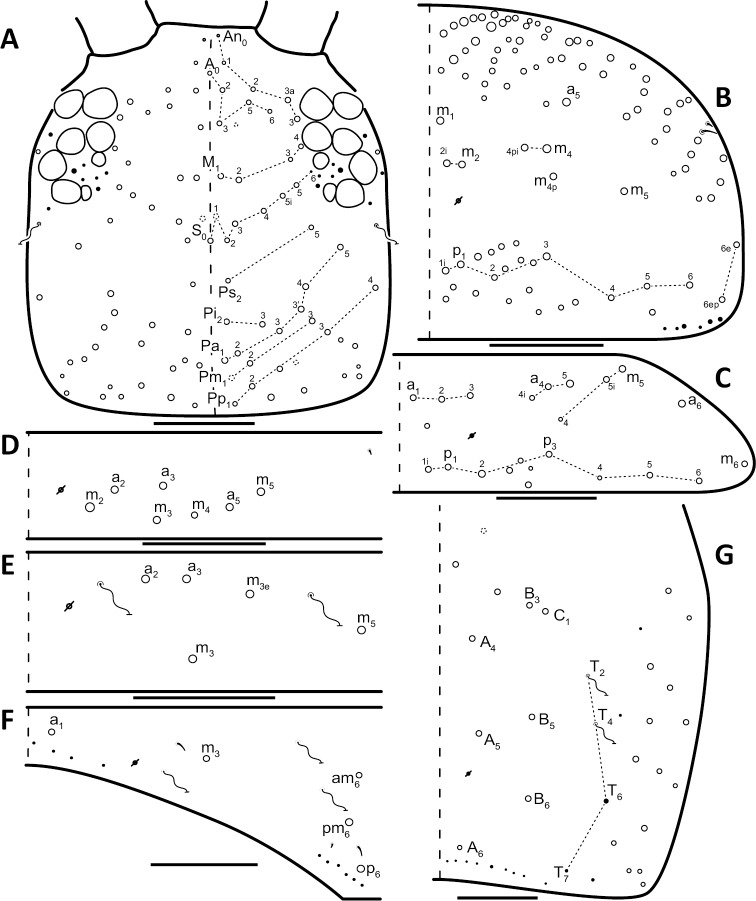
Dorsal chaetotaxy of *Entomobrya intermedia*: **A** Head **B** Mesothorax **C** Metathorax **D** 1^st^ abdominal segment **E** 2^nd^ abdominal segment **F** 3^rd^ abdominal segment **G** 4^th^ abdominal segment. Scale bars = 100 µm. See Figure 5 for symbol legend.

Figs 2 , 21 , 22 , 39

###### Description.

*Body shape and color pattern*. Body oval and cylindrical. Color pattern monomorphic (Fig. 21): yellow background with black, dark brown or purple pigment forming two incomplete, broken, longitudinal bands from Th. 2 through Abd. 3 and a conspicuous W-shaped mark on Abd. 4. Usually with dark pigment covering the lateral margins of the head. Antennae light brown or purple becoming increasingly dark towards the apex.

*Head*. Apical bulb of 4^th^ antennal segment simple. Long differentiated smooth setae on ventral side of 1^st ^antennal segment ≈5× short setae. Prelabral setae ciliate. Ornamentation of the distal margin of the labral papillae with 2-4 small spine-like projections (Fig. 2). Lateral appendage of labial papilla E slightly curved, thin, nearly reaching tip of papilla. Dorsal head chaetotaxy (Fig. 22A) with macrosetae An’_0_, A_3a2_, A_3a3_, M_3i_, S’_0_, S_4i_, S_6_, Ps_3_, Pi_1_, and Pm_1i_ absent; an additional macroseta external to A_3_ present in some specimens; S_1_ and Pm_1_ usually present, but may be asymmetrical. Eyes G and H small and subequal. Eye patch with 5 setae.

*Thorax*: Thoracic chaetotaxy well-developed and relatively stable. Th. 2 macrosetae a5, m_1_, m_2_, m_2i_, m_4_, m_4p_, m_4i_, and m_5_ present (Fig. 22B). Lateral sensilla obscured in all specimens examined. Posterior macrosetae (series P) present. Th. 3 macrosetae a_1_, a_2_, a_3_, a_4_, a_4i_, a_5_, a_6_, m_4_, m_5_, m_5i_, and m_6 _present (Fig. 22C). Posterior macrosetae (series P) present. The chaetotaxy of zone Pm with a moderate number of supplemental macrosetae in both thoracic segments (Fig. 22B,C). Typical sensilla present (not shown in figure).

*Legs*. Trochanteral organ with triangular setal pattern and up to 31 setae. Unguis with 4 internal teeth; basal teeth located approximately middle of inner claw length.

*Abdomen*. Abdominal chaetotaxy stable. Abd. 1 with 7 macrosetae (Fig. 22D). Abd. 2 macroseta a_2_, a_3_, m_3_, m_3e_, and m_5_ present (Fig. 22E). Abd. 3 with macrosetae a_1_, m_3_, am_6_, pm_6_, and p_6_ (Fig. 22F). Abd. 4 with 9-10 inner macrosetae (Fig. 22G). Mucronal teeth subequal. Typical sensilla present (not shown in figure).

###### Remarks.

*Entomobrya intermedia* can be easily identified by the presence of two longitudinal stripes, a W-shaped mark on Abd. 4 combined with the presence of Th. 2 macrosetae m_5_ and Abd. 3 a_1_, and the absence of head macrosetae S_4i_, Abd. 2 m_3ep_, and Abd. 3 a_2_ (see Table 2 for additional diagnostic characters). Historically, this species was considered a synonym of *Entomobrya nivalis*. However, the clear differences in chaetotaxy (see Table 3) and color pattern separate *Entomobrya intermedia* from *Entomobrya nivalis* and other similar forms such as *Entomobrya multifasciata* and female *Entomobrya atrocincta*. Furthermore, molecular data supports the separation of this species from *Entomobrya nivalis* (Katz et al. 2015). The combination of chaetotaxy outlined in Table 3 should be used in conjunction with color pattern characters to differentiate this species. Note that observation of chaetotaxy was only examined for specimens collected from Chester County, Pennsylvania. It is likely that specimens from additional localities may reveal more variation in chaetotaxy than described here, especially since the chaetotaxy reported in Palearctic specimens (Jordana 2012) is different than those observed in North American specimens.

###### Distribution.

North America and Europe. The actual distribution of *Entomobrya intermedia* in North America is unclear, as before Christiansen and Bellinger’s (1998) monograph the species was included within *Entomobrya nivalis* (Christiansen 1958b). In fact, some records of *Entomobrya nivalis*, *Entomobrya multifasciata* and female *Entomobrya atrocincta*, regardless of date, may actually refer to *Entomobrya intermedia*. Suppl. material 1: G shows the distribution of *Entomobrya intermedia* in North American as currently understood.

###### Material examined.

USA: 2 on slides, Pennsylvania, Chester Co., Wayne, sweep of *Forsythia* sp., 23.v.2011, AK11-32; 8 on slides, 12 in vial, Pennsylvania, Chester Co., Wayne, sweep of *Forsythia* sp., 29.vi.2012, AK12-50.

##### Entomobrya jubata

Taxon classificationAnimaliaCollembolaEntomobryidae

Katz & Soto-Adames sp. n.

http://zoobank.org/B178F030-EA9E-43C6-99B3-98C89487F3EB

**Figure 23.**
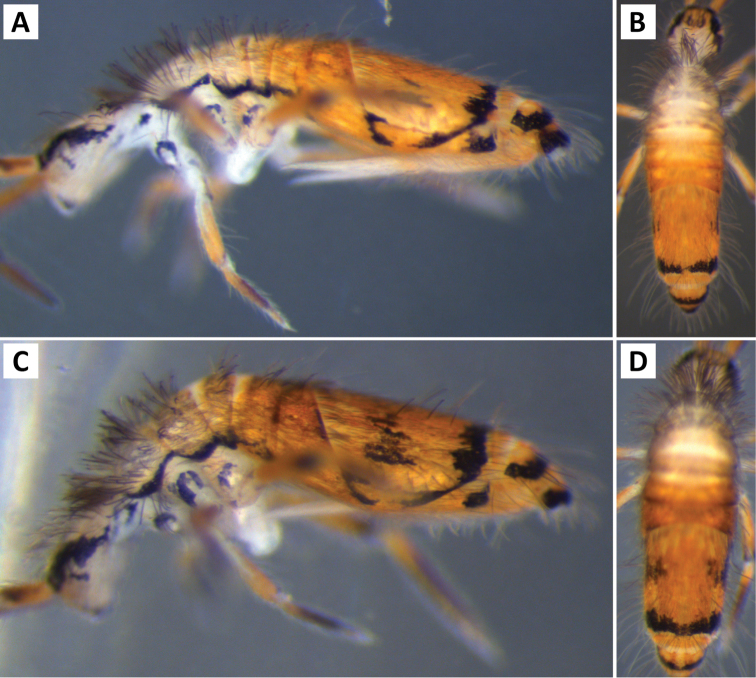
Dorsal color patterns of *Entomobrya jubata* sp. n. collected for this study. Lateral and dorsal views for two individuals (**A, B** and **C, D**) collected from Covington Co., AL.

**Figure 24.**
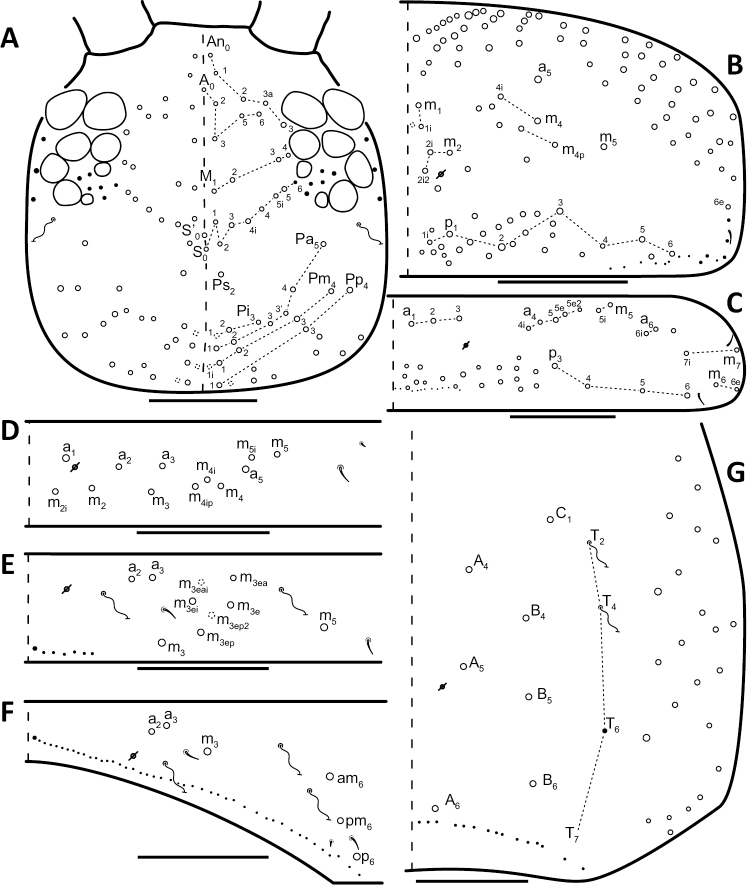
Dorsal chaetotaxy of *Entomobrya jubata* sp. n.: **A** Head **B** Mesothorax **C** Metathorax **D** 1^st^ abdominal segment **E** 2^nd^ abdominal segment (Abd. 2) **F** 3^rd^ abdominal segment **G** 4^th^ abdominal segment. Scale bars = 100 µm. See Figure 5 for symbol legend.

Figs 16B , 23 , 24 , 39

###### Etymology.

The word jubatus is Latin for maned, or crested, and refers to the abundance of dorsal macrosetae on the thoracic segments.

###### Type material.

*Holotype*, ♀, USA: Alabama, Covington County, Conecuh National Forest (31.07900,-86.61203), under bark, 2.i.12 (A. Katz & M. DuBray), AK12-9 & AK12-6.

*Paratypes*, USA: 7 on slides, 20 in vials, Alabama, Covington Co., Conecuh National Forest (31.07900,-86.61203), under bark, 2.i.12 (A. Katz & M. DuBray), AK12-9 & AK12-6.

###### Description.

*Body shape and color pattern.* Body cylindrical, slightly dorso-ventrally flattened. Length up to 2 mm. Color pattern monomorphic (Fig. 23): light brown background with black pigment forming dark transverse bands across the posterior margins of Abd. 4, Abd. 5, and Abd. 6; dark pigment present along lateral margins of Th. 2 through Abd. 2, forming two lateral stripes. Two patches of dark pigment usually present medially on Abd. 4 and may appear to form an incomplete irregular transverse band; Th. 2 entirely white except for black pigment lining anterior and lateral margins; legs range in color from white to light brown to purple near the apex.

*Head*. Apical bulb of 4^th^ antennal segment usually simple, rarely bilobed. Long differentiated smooth seta on ventral side of 1^st ^antennal segment 3× as long as short setae. Prelabral setae ciliate. Distal margin of the labral papillae with 2-3 seta or spine-like projections. Labial papilla E with lateral appendage almost straight, reaching tip of papilla. Dorsal head chaetotaxy as in Figure 24A: macrosetae An’_0_, A_3a2_, A_3a3_, M_3i_, S_6_, Ps_3_, and Ps_5_ always absent; Pi_1_ and Pm_1i_ present or absent; S’_0_ always present. Eyes G and H small and subequal. Eye patch with 5 setae.

*Thorax*. Thoracic chaetotaxy well-developed (Fig. 24B). Th. 2 with all described macrosetae present, except m_4i3_. Th. 3 macrosetae a_5e3_, m_4_, m_5p_, and a_7 _absent (Fig. 24C). Zone Pm with many supplemental macrosetae present in both thoracic segments.

*Legs*. Trochanteral organ with triangular setal pattern and up to 25 setae. Unguis with 4 inner teeth; basal teeth located approximately middle of inner claw length (Fig. 16B).

*Abdomen*. Abdominal chaetotaxy abundantly developed. Abd. 1 with 12 macrosetae (Fig. 24D). Abd. 2 macrosetae variable: a_2_, a_3_, m_3_, m_3ep_, m_3e_, m_3ei_, m_3ea_, and m_5_ always present; m_3ep2_ and m_3eai_ sometimes present (Fig. 24E). Abd. 3 macrosetae a_2_, a_3_, m_3_, am_6_, pm_6_, and p_6_ present; a_1_ always absent (Fig. 24F). Abd. 4 chaetotaxy stable, with 7 inner macrosetae (Fig. 24G). Mucronal teeth subequal.

###### Remarks.

*Entomobrya jubata* sp. n. can be easily distinguished by the unique color pattern described above combined with the absence of head macroseta Ps_5_, the presence of head macroseta S’_0_ and Th. 2 macrosetae m_2_ and m_5_ (see Table 2 for additional diagnostic characters). This species is closely related to *Entomobrya clitellaria* and both share similar chaetotaxy. However, these species can be easily separated by color pattern alone; *Entomobrya jubata* sp. n. does not have dark pigment on Th. 3 through Abd. 3. The presence of head macrosetae S’_0_ and the absence of head macrosetae Ps_5_ also separate *Entomobrya jubata* sp. n. from *Entomobrya clitellaria*. The color pattern and chaetotaxy exhibited by this species have not been reported in the literature.

###### Distribution.

Endemic to North America. *Entomobrya jubata* sp. n. was collected from a single locality in Covington County, Alabama (Suppl. material 2: H).

##### Entomobrya ligata

Taxon classificationAnimaliaCollembolaEntomobryidae

Folsom, 1924

Figs 25 , 26 , 39

###### Description.

**Figure 25.**
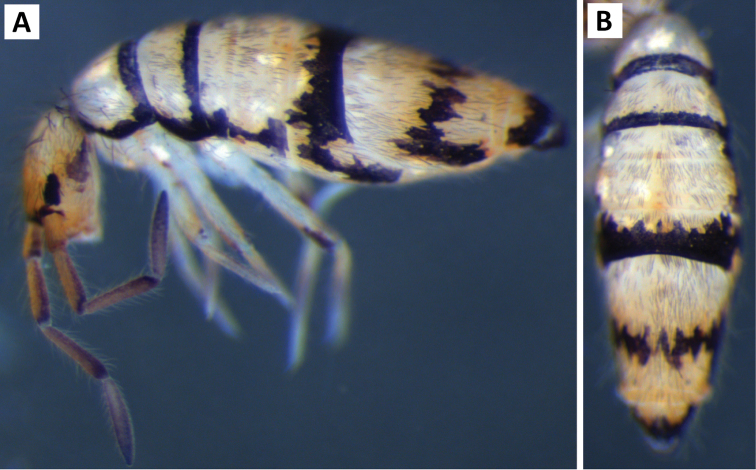
Color pattern of *Entomobrya ligata*. Lateral (**A**) and dorsal (**B**) views of specimen collected from Chester Co., PA.

*Body shape and color pattern.* Body oval and cylindrical. Color pattern stable, monomorphic (Fig. 25), always with four transverse bands; two thin regular bands along the posterior margin of Th. 2 and Th. 3 respectively, an irregular, patchy, thick band covering most of Abd. 3, and a highly irregular and sometimes broken band across the medial section of Abd. 4. A small patch of pigment covers Abd. 5 and Abd. 6. Patterns usually consisting of black or dark blue pigment with a yellow background. Dark pigment usually in patches along lateral margins of Th. 2 through Abd. 4. Antennae with purple pigment, darken near apex. Legs usually white, with small purple patches on apical end of femora.

**Figure 26.**
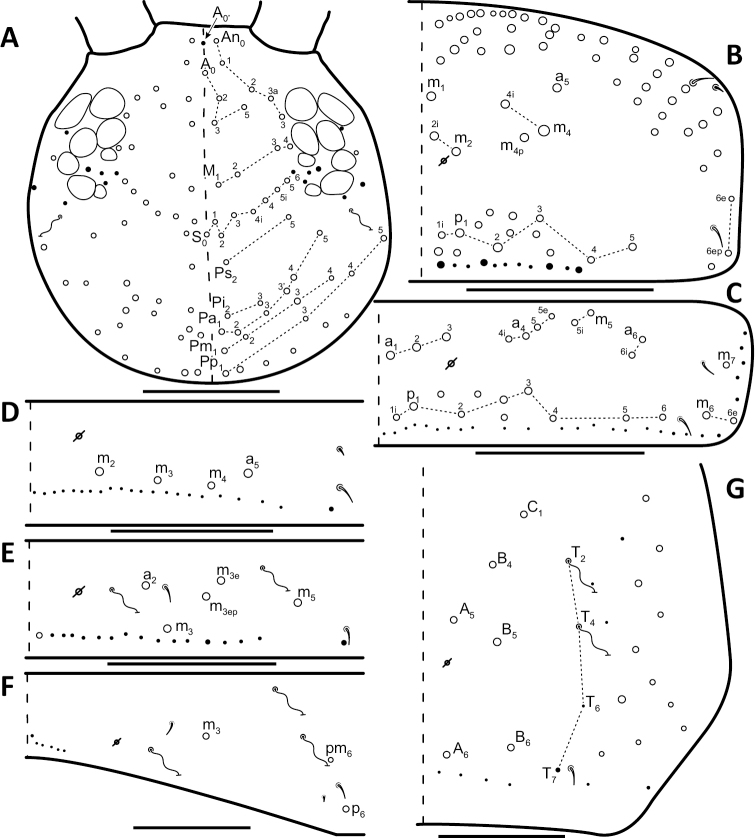
Dorsal chaetotaxy of *Entomobrya ligata*: **A** Head **B** Mesothorax **C** Metathorax **D** 1^st^ abdominal segment **E** 2^nd^ abdominal segment **F** 3^rd^ abdominal segment **G** 4^th^ abdominal segment. Scale bars = 100 µm. See Figure 5 for symbol legend.

*Head.* Apical bulb of 4^th^ antennal segment usually bilobed, sometimes multilobed. Long differentiated smooth setae on ventral side of 1^st ^antennal segment ≈3× short setae. Prelabral setae finely ciliated, seemingly smooth at low magnification under light microscopy. Ornamentation of the distal margin of the labral papillae with single seta or spine-like projections. Lateral appendage of labial papilla E slightly curved, relatively thick and short, extending only ¾ papilla length. Dorsal head macrosetae (Fig. 26A) An_3a2_, An_3a3_, A_6_, M_3i_, S’_0_, S_6_, Ps_3_, Pi_1_, Pm_1i_, and Pp_2_ absent; An’_0_, a short mesoseta located medially between both An_0_ present. Eyes G and H small and subequal. Eye patch with 3 setae.

*Thorax*. Th. 2 macrosetae a5, m_1_, m_2_, m_2i_, m_4_, m_4p_, and m_4i_ present (Fig. 26B). Th. 3, macrosetae a_1_, a_2_, a_3_, a_4_, a_4i_, a_5_, a_5e_, a_6_, a_6i_, m_5_, m_5i_, m_6_, m_6e_, and m_7 _present (Fig. 26C); all posterior macrosetae (series P) present. Zone Pm in both thoracic segments with a moderate number of supplemental macrosetae (Fig. 26B,C).

*Legs*. Trochanteral organ with triangular setal pattern and up to 17 setae. Unguis with 4 internal teeth; basal teeth located approximately middle of inner claw length.

*Abdomen*. Abdominal chaetotaxy reduced; no macrosetae variation observed. Abd. 1 with 4 macroseta: a_5_, m_2_, m_3_, and m_4_ (Fig. 26D). Abd. 2 with 5 macrosetae: a_2_, m_3_, m_3e_, m_3ep_, and m_5_ (Fig. 26E). Abd. 3 with 3 macrosetae: m_3_, pm_6_, and p_6_ (Fig. 26F). Abd. 4 with 6 inner macrosetae (Fig. 26G). Mucronal teeth subequal.

###### Remarks.

This species can be identified by the presence of four transverse bands, head mesoseta An’_0 _present, four macrosetae on Abd. 1, and only three eye patch setae (see Table 2 for additional diagnostic characters). *Entomobrya unifasciata* sp. n. and *Entomobrya neotenica* sp. n. are closely related to this species and form the *Entomobrya ligata* complex, characterized by the presence of only three microsetae in the eye patch, four macrosetae on Abd. 1, and six macrosetae on Abd. 4. *Entomobrya ligata* can be separated from *Entomobrya unifasciata* sp. n. and *Entomobrya neotenica* sp. n. by characters outlined in Table 6.

**Table 6.** Diagnostic characters to separate species within the ligata complex: *Entomobrya ligata*, *Entomobrya unifasciata* sp. n., and *Entomobrya neotenica* sp. n. Character states: absent (0), present (1).

Species	Head mesoseta An’_0_	Abd. 3 macroseta m_3ep_	1 dark transverse band on posterior margin of Th. 2	2 dark triangular patches on Abd. 3
*Entomobrya ligata*	1	1	1	0
*Entomobrya unifasciata* sp. n.	1	1	0	0
*Entomobrya neotenica* sp. n.	0	0	0(1)^1^	1

1 Parentheses indicate a rarely observed state.

*Entomobrya ligata* was described by Folsom (1924) and redescribed by Christiansen (1958b). Both descriptions depict *Entomobrya ligata* with four dorsal transverse bands (Folsom described five bands; he considered the pigment on Abd. 5 and 6 an additional band), two of which occur on the posterior margins of the Th. 2 and Th. 3, respectively. Samples were originally diagnosed as *Entomobrya ligata* based on chaetotaxy described by Christiansen and Bellinger (1998). However, Katz et al. (2015) showed that a population from Chester Co., Pennsylvania was highly divergent and genetically isolated from other populations. The Pennsylvania population differs from all other populations by the presence of a dark transverse band along the posterior margin of Th. 2. This dark band is present in all individuals collected in Pennsylvania and absent in individuals from all other localities. The original descriptions by Folsom (1924) and Christiansen (1958b) described *Entomobrya ligata* as having this band present and noted a lack of additional variations in color form. Multiple type specimens deposited at the INHS were examined; all collected in the state of New York, and all carry a dark band along the posterior margin of Th. 2 (Suppl. material 3: D–G). Therefore, the combination *Entomobrya ligata* is reserved for populations in which individuals carry the posterior band on Th. 2, whereas populations without this band are referred to *Entomobrya unifasciata* sp. n. (see below).

###### Distribution.

Endemic to North America. The species has been reported as having a wide distribution, occurring east of the Mississippi River to the Atlantic coast (Suppl. material 2: I). However, in light of the new circumscription provided above and the possible confusion with *Entomobrya unifasciata* sp. n., most historical reports are questionable, especially those between the western Smokey Mountains and the Mississippi River. The syntypic series of *Entomobrya ligata* was collected at different localities in New York State and all fresh material was collected in Chester Co., Pennsylvania, suggesting the species may be restricted to the northeast section of the country.

###### Material examined.

USA: *Syntypes*, 1 on slide, Karner, N.Y., 7-14-23 (A. Wolf) INHS Cat. No. 528,351; *Cotypes*: 1 in vial, Karner, N.Y., July, 14 1923 (A. Wolf); *Cotypes*: 2 in vial, N.Y., July 8, 1923 (A. Wolf); *Syntypes*, 1 on slide, Mineola, L. I., N.Y., July 8, 1923 (O. W. Barrett) INHS Cat. No. 528,350; *Cotypes*, 1 in vial, Roorhesville, N. Y., Aug 30, 1923 (M. S. Leonard); *Cotypes*: 2 in vial, Roorhesville, N. Y., Aug 30, 1923 (M. S. Leonard). Other material: 1 on slide, 1 in vial, Pennsylvania, Chester Co., Wayne, McKaig Nature Education Center (40.06923,-75.37903), leaf litter, 23.v.2011, AK11-33; 1 on slide Pennsylvania, Chester Co., Wayne, McKaig Nature Education Center (40.06923,-75.37903), bark, 23.v.2011, AK11-34.

##### Entomobrya multifasciata

Taxon classificationAnimaliaCollembolaEntomobryidae

(Tullberg), 1871

**Figure 27.**
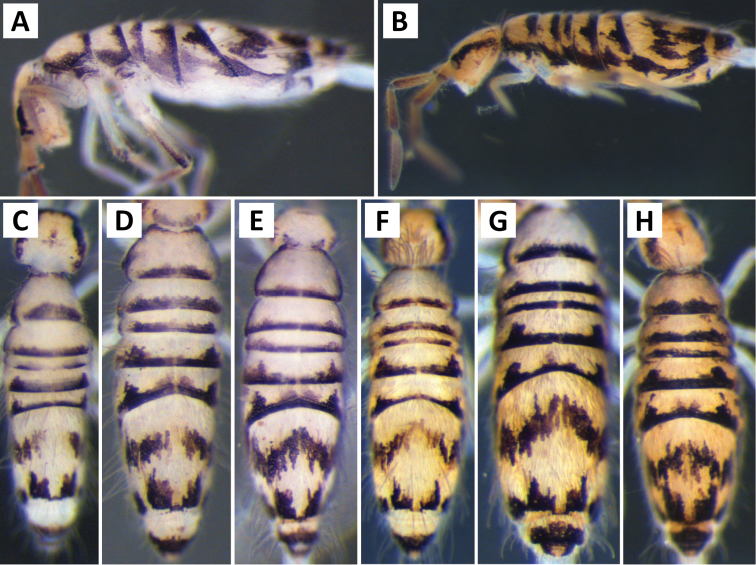
Color patterns of *Entomobrya multifasciata*. All photographed specimens (**A–H**) are from São Miguel Island, Azores, Portugal.

**Figure 28.**
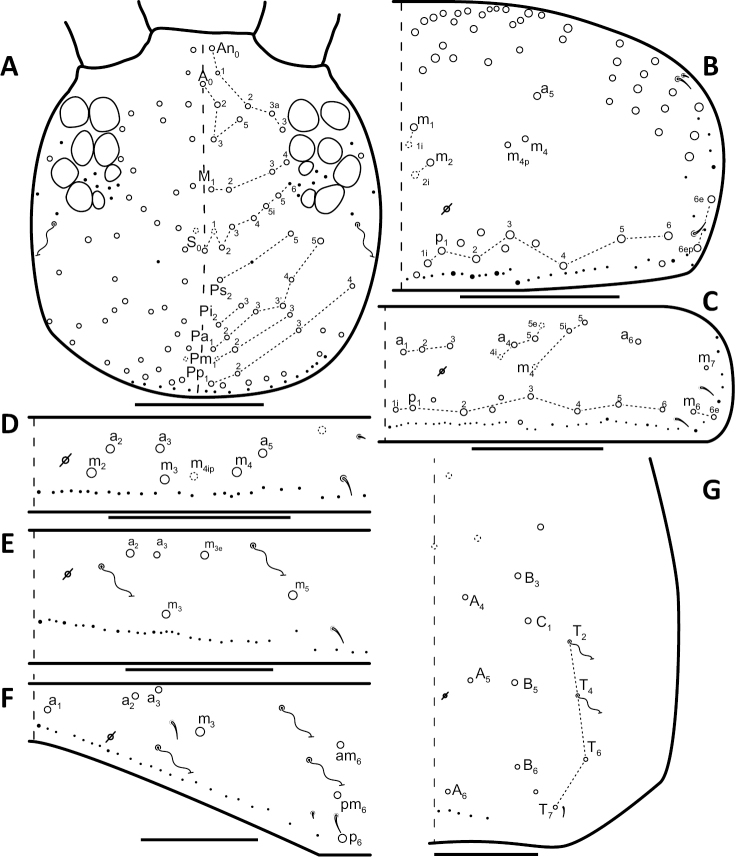
Dorsal chaetotaxy of *Entomobrya multifasciata*: **A** Head **B** Mesothorax **C** Metathorax **D** 1^st^ abdominal segment **E** 2^nd^ abdominal segment **F** 3^rd^ abdominal segment **G** 4^th^ abdominal segment. Scale bars = 100 µm. See Figure 5 for symbol legend.

Figs 2 , 27 , 28 , 39

###### Description.

*Body shape and color pattern*. Body oval and cylindrical. One primary color form, with slight variations (Fig. 27); yellow background with black, dark brown or purple pigment forming 5 transverse bands along posterior margins of Th. 2 through Abd. 3. Abd. 4 pattern variable, but usually with 2 triangular patches of pigment along posterior margin of segment that point anteriorly toward a W-shaped mark or broken and irregular transverse band. Abd. 5 and Abd. 6 mostly covered with dark pigment. Dark pigment present along lateral margins of all segments, sometimes in broken patches. Antennae light brown or purple pigment increasingly dark towards the apex.

*Head*. Apical bulb of 4^th^ antennal segment usually bilobed, sometimes simple. Long differentiated smooth setae on ventral side of 1^st ^antennal segment ≈2–3× short setae. Four prelabral setae ciliate. Ornamentation of the distal margin of the labral papillae with 2-3 seta or spine-like projections (Fig. 2). Labial lateral appendage slightly curved, relatively thin, reaching just beyond tip of papilla. Dorsal head macrosetae (Fig. 28A) An’_0_, A_3a2_, A_3a3_, M_3i_, S’_0_, S_4i_, S_6_, Ps_3_, Pi_1_, and Pm_1i_ absent; S_1_ and Pm_1 _usually present, but may be asymmetrical. Eyes G and H small and subequal. Eye patch with 5 setae.

*Thorax*. Th. 2 macrosetae a5, m_1_, m_2_, m_4_, and m_4p_ present (Fig. 28B); m_1i_ and m_2i_ sometimes present; posterior macrosetae (series P) present. Th. 3 macrosetae a_1_, a_2_, a_3_, a_4_, a_5_, a_6_, m_5_, m_5i_, m_6_, m_6e_, and m_7_ present (Fig. 28C); a_4i_, a_5e_, and m_4_ sometimes present; posterior macrosetae (series P) present. Zone Pm of both thoracic segments with moderate number of supplemental macrosetae (Figs 3; 39B,C).

*Legs*. Trochanteral organ with triangular setal pattern and up to 18 setae.

*Abdomen*. Abd. 1 with 6-8 macrosetae (Fig. 28D). Abd. 2 macroseta a_2_, a_3_, m_3_, m_3e_, and m_5_ are present (Fig. 28E). Abd. 3 macroseta a_1_, a_2_, a_3_, m_3_, am_6_, pm_6_, and p_6_ present (Fig. 28F). Abd. 4 with 8-12 inner macroseta (Fig. 28G). Mucronal teeth subequal.

###### Remarks.

*Entomobrya multifasciata* can be distinguished by the color pattern described above combined with the presence of Abd. 3 macrosetae a_1_ and a_2_ and the absence of macrosetae head S_4i_, Th. 2 m_5_, and Abd. 2 m_3ep _(see Table 2 for additional diagnostic characters). Chaetotaxy and color pattern observed in this species is almost indistinguishable from that in female *Entomobrya atrocincta*. Furthermore, variation in chaetotaxy displayed in *Entomobrya atrocincta* obscures most potentially diagnostic characters needed to distinguish *Entomobrya multifasciata* from *Entomobrya atrocincta*. However, *Entomobrya multifasciata* can be recognized by the presence of head macroseta S_4i_ and the morphology of labral papillae; *Entomobrya multifasciata* has two to three larger seta or spine-like projections per papillae, while *Entomobrya atrocincta* has three to four smaller seta or spine-like projections per papillae (Fig. 2). Table 3 provides additional diagnostic characters separating *Entomobrya multifasciata* from *Entomobrya nivalis* and *Entomobrya intermedia*, which share superficially similar color patterns but can be separated by chaetotaxy.

We were unable to obtain North American samples of *Entomobrya multifasciata* and the description and diagnosis provided above are based on specimens from São Miguel Island, Azores, Portugal. The Nearctic distribution of this species remains unclear in light of the sexual dimorphism of *Entomobrya atrocincta* described in this study (see remarks for *Entomobrya atrocincta*). Christiansen and Bellinger (1998) report a widespread distribution, with localities found across the United States, but they also note that their records may be questionable as a result of likely misidentification. Christiansen and Bellinger (1998) describe the labral papillae of *Entomobrya multifasciata* as having three to four small seta or spine-like projections, a condition that resembles those in *Entomobrya atrocincta*, possibly indicating an identification error. In the Collembola of North America, Christiansen and Bellinger (1998) report a male genital plate, presumably from an individual with the *Entomobrya multifasciata* pattern collected in Massachusetts, which eliminates the possibility that the specimen could have been a female *Entomobrya atrocincta*. Since a number of samples were collected in the Northeastern United States (Christiansen and Bellinger 1998), the presence of *Entomobrya multifasciata* in North America cannot be ruled out. However, in the course of the present study, all specimens collected bearing the banded color pattern were female *Entomobrya atrocincta* and were usually accompanied by male *Entomobrya atrocincta*.

###### Distribution.

North America (Christiansen and Bellinger 1998), Hawaii (Christiansen and Bellinger 1992), Europe and Russia (Jordana 2012). Records from North America and Hawaii are questionable due to likely misidentification of *Entomobrya atrocincta* females. See Suppl. material 2: J for a distribution map and below for a list of material examined with collection and locality information.

###### Material examined.

PORTUGAL: 2 on slides, 10 in vial, Azores, São Miguel Island, (J. Marcelino), vial #119; 6 on slides, 10 in vial, Azores, São Miguel Island, (J. Marcelino), vial #86; 5 in vial, Azores, São Miguel Island, (J. Marcelino), vial #55; 4 in vial, Azores, Terceira Island, (J. Marcelino), vial #9; 4 in vial, Madeira Island, (J. Marcelino), vial #2.

##### Entomobrya neotenica

Taxon classificationAnimaliaCollembolaEntomobryidae

Katz & Soto-Adames sp. n.

http://zoobank.org/C70B2A90-547A-4A0F-9A4E-E5F33B83BF8A

**Figure 29.**
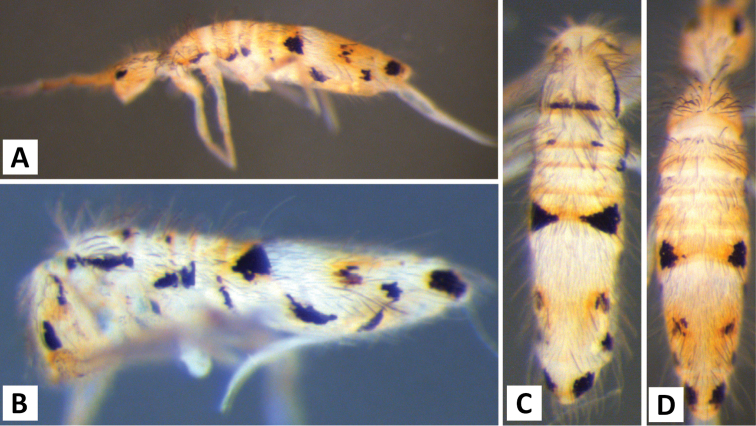
Color pattern of *Entomobrya neotenica* sp. n. Lateral and dorsal views of specimens collected from Lawrence Co., AL (**A** and **D**) and Clay Co., AL (**B** and **C**).

**Figure 30.**
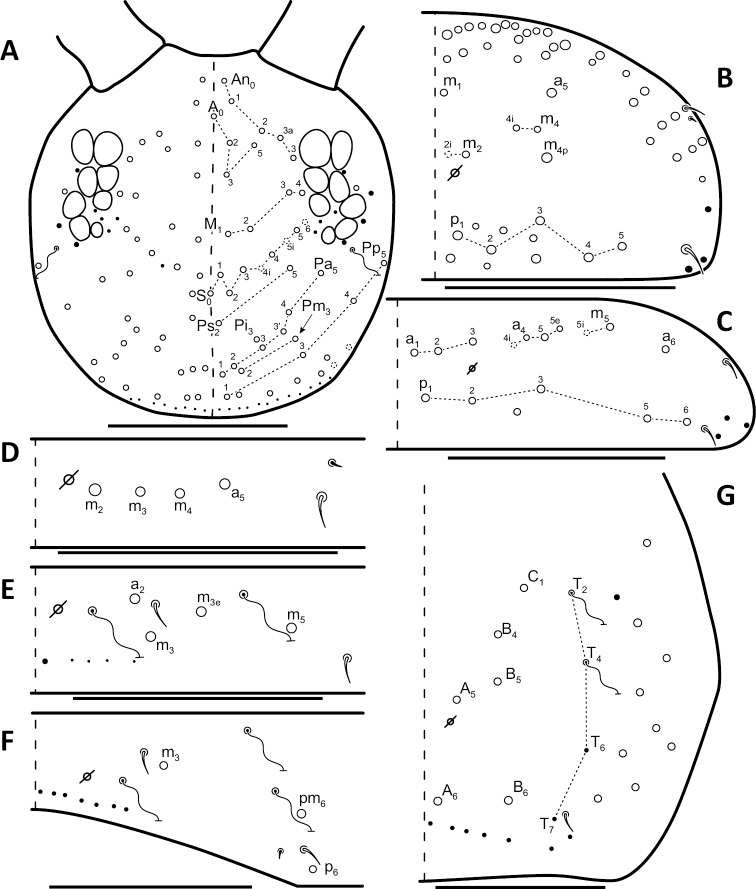
Dorsal chaetotaxy of *Entomobrya neotenica* sp. n.: **A** Head **B** Mesothorax **C** Metathorax **D** 1^st^ abdominal segment **E** 2^nd^ abdominal segment **F** 3^rd^ abdominal segment **G** 4^th^ abdominal segment. Scale bars = 100 µm. See Figure 5 for symbol legend.

Figs 2 , 16C , 29 , 30 , 39

###### Etymology.

This species is named for its apparent neoteny; small size and reduced chaetotaxy.

###### Type material.

*Holotype*, ♂, USA: Alabama, Lawrence County, William B. Bankhead National Forest (34.3369,-87.3461), leaf litter, 9.viii.2011, AK11-112.

*Paratypes*, USA: 1 on slide, 1 in vial, Alabama, Clay Co., Talladega National Forest (33.19723,-86.06325), moist leaf litter, 2.i.12 (A. Katz & M. DuBray), AK12-2; 1 in vial, Alabama, Lawrence Co., William B. Bankhead National Forest (34.3369,-87.3461), leaf litter, 9.viii.2011, AK11-112; 1 on slide, Florida, Taylor Co., Econfina River State Park (30.0656,-83.9107), under bark, 9.viii.2011, AK11-116; 2 on slides, 18 in vial, Illinois, Union Co., Anna, Shawnee National Forest, Rich’s Cave, moist leaf litter under bark on fallen tree, 21.vi.2012 (F. Soto-Adames, S. Taylor & A. Katz); 1 on slide, Tennessee, Stewart Co., Land Between the Lakes National Recreation Area (36.5354,-87.9214), forest floor leaf litter, 3.v.2011, AK-43.

###### Description.

*Body shape and color pattern*. Body oval and cylindrical. Length up to 1.15 mm. One primary color form (Fig. 29): white and yellow or orange background with black or dark purple pigments forming two lateral triangles on the sides Abd. 3; triangles sometimes reduced to irregular patches. Additional irregular patches of pigment usually on lateral margins of all segments. A band sometimes present along posterior margin of Th. 2. Abd. 5 with 2 dark spots, sometimes forming irregular triangular shapes. Antennae usually light purple near apex and relatively long. Legs usually white, with small purple patches on apical end of femora.

*Head*. Apical bulb of 4^th^ antennal segment simple or bilobed. Long differentiated smooth setae on ventral side of 1^st ^antennal segment ≈2–2.5× short setae. Four prelabral setae finely ciliate, appearing smooth under light microscopy. Ornamentation of the distal margin of the labral papillae with single seta or spine-like projections (Fig. 2). Labial lateral appendage slightly curved, relatively thick, not reaching tip of papilla. Dorsal head chaetotaxy reduced (Fig. 30A), macrosetae An’_0_, An_3a2_, An_3a3_, A_6_, M_3i_, S’_0_, Ps_3_, Pi_1_, Pi_2_, Pm_1_, Pm_1i_, and Pp_2_ absent; S_5i_ usually present; S_4i_ and S_6_ usually absent. Eyes G and H smaller than A-F but enlarged; G slightly larger than H. Eye patch with 3 setae.

*Thorax*. Thoracic chaetotaxy reduced, with relatively few supplementary setae. Th. 2 macrosetae a5, m_1_, m_2_, m_4_, m_4p_, and m_4i_ present (Fig. 30B); all posterior macrosetae (series P) present. Th. 3 macrosetae a_1_, a_2_, a_3_, a_4_, a_4i_, a_5_, a_5e_, a_6_, a_6i_, m_5_, m_5i_, m_6_, m_6e_, and m_7_ present (Fig. 30C). Both thoracic segments with few supplemental macrosetae present in zone Pm (Fig. 30B,C).

*Legs*. Trochanteral organ with triangular setal pattern and up to 20 setae. Unguis with 4 inner teeth (Fig. 16C). Unguiculus acuminate with small serrations on inner edge.

*Abdomen*. Abdominal chaetotaxy reduced; no macrosetae variation observed. Abd. 1 with 4 macrosetae; a_5_, m_2_, m_3_, and m_4_ (Fig. 30D). Abd. 2 with 4 macrosetae: a_2_, m_3_, m_3e_, and m_5_ (Fig. 30E). Abd. 3 with 3 macrosetae: m_3_, pm_6_, and p_6_ (Fig. 30F). Abd. 4 with 6 inner macrosetae (Fig. 30G). Mucronal teeth subequal; mucronal spine smooth.

###### Remarks.

*Entomobrya neotenica* sp. n. can be diagnosed by the presence of two lateral dark triangular shaped or irregular spots on Abd. 3, only 3 setae in eye patch, and the absence of head mesoseta An’_0_ and Abd. 3 macroseta m_3ep_ (see Table 2 for additional diagnostic characters). This species is included in the *Entomobrya ligata* complex (see remarks for *Entomobrya ligata*) and has a unique color pattern and chaetotaxy never before reported in the literature. *Entomobrya neotenica* sp. n. is exceptionally small compared to most Nearctic *Entomobrya*. In fact, most individuals were thought to be juvenile forms of *Entomobrya ligata* prior to the observation of the male genital plate. *Entomobrya neotenica* sp. n. is very similar to *Entomobrya ligata* and *Entomobrya unifasciata* sp. n., but can be separated by characters outlined in Table 6.

###### Distribution.

Endemic to North America (Suppl. material 2: K)

##### Entomobrya nivalis

Taxon classificationAnimaliaCollembolaEntomobryidae

(Linnaeus), 1758

**Figure 31.**
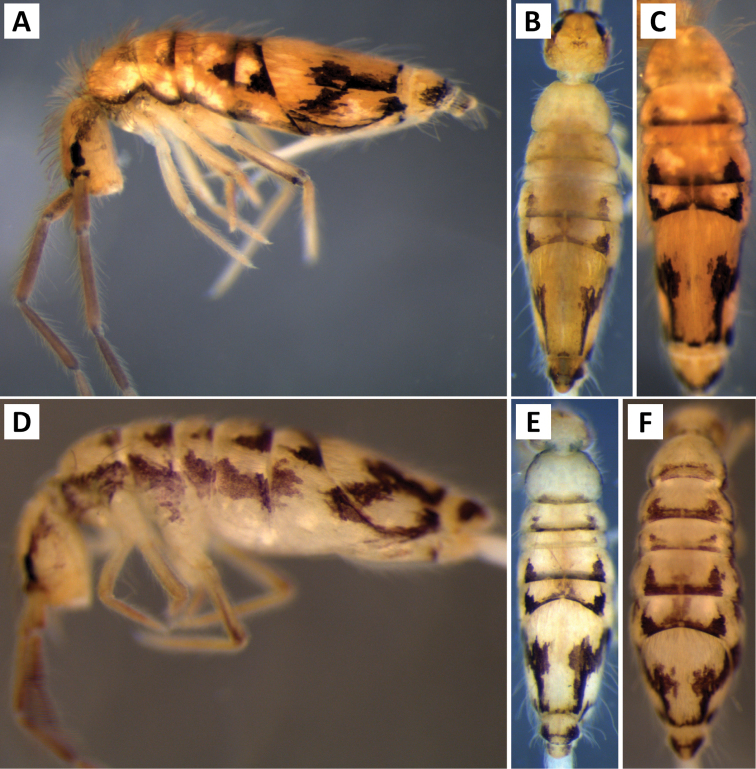
Color pattern of *Entomobrya nivalis* collected from: **A** Lamoille Co., VT **B** Sauk Co., WI **C** Hancock Co., ME **D** Lamoille Co., VT **E** Hancock Co., ME **F** Sauk Co., WI.

**Figure 32.**
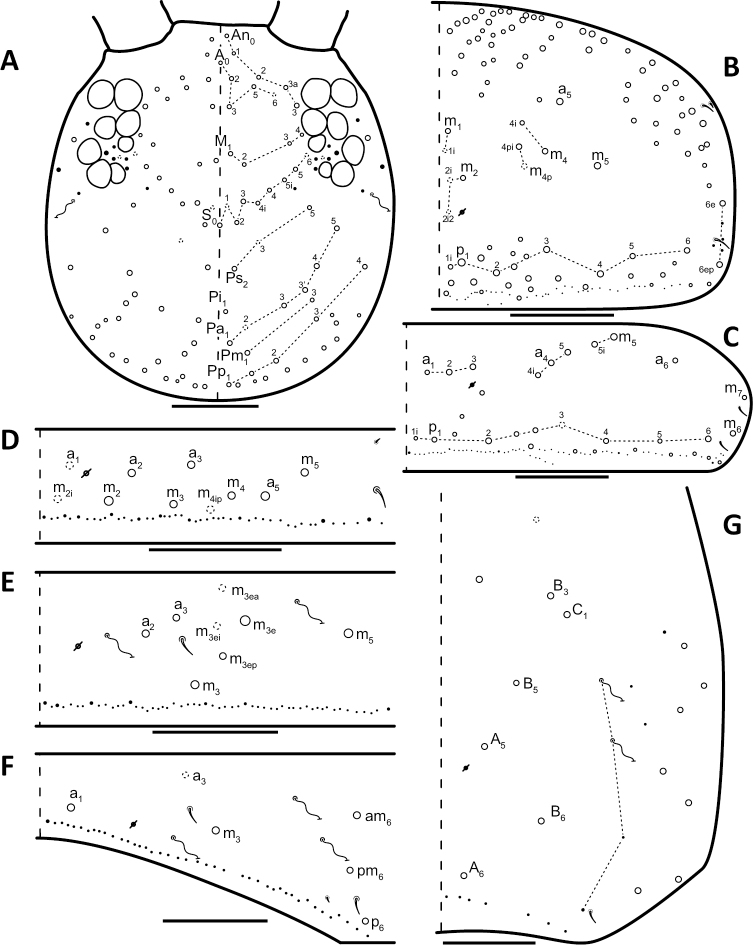
Dorsal chaetotaxy of *Entomobrya nivalis*: **A** Head **B** Mesothorax **C** Metathorax **D** 1^st^ abdominal segment **E** 2^nd^ abdominal segment **F** 3^rd^ abdominal segment **G** 4^th^ abdominal segment. Scale bars = 100 µm. See Figure 5 for symbol legend.

Figs 2 , 31 , 32 , 39

###### Description.

*Body shape and color pattern*. Body cylindrical. One primary, but variable, color form (Fig. 31): yellow or white background with black, dark brown or purple pigment always forming thin transverse bands along the posterior margin of Th. 3, and Abd. 2 through Abd. 6. Additional transverse bands present or absent on Th. 2 and Abd. 1. Abd. 4 usually with U-shaped or “11”-shaped pattern connecting basally with band along posterior margin. Antennae usually lack dark pigmentation, sometimes with light brown or purple pigment, darkening near the apex.

*Head*. Apical bulb of 4^th^ antennal segment usually bi- or trilobed. Long differentiated smooth setae on ventral side of 1^st^ antennal segment ≈3× short setae. Ornamentation of the distal margin of the labral papillae with 3-4 small seta or spine-like projections per papilla (Fig. 2). Labial lateral appendage slightly curved, relatively thick, nearly reaching tip of papilla. Labial triangle chaetotaxy normal, one specimen with a supplemental ciliate seta internal to M1 (one side only). Dorsal head chaetotaxy (Fig. 32A) with macrosetae An’_0_, A_3a2_, A_3a3_, M_3i_, S’_0_, Ps_3_, Pi_2_, Pi_3_, Pm_1i_, and Pm_2_ absent; S_1_ usually present, A_6_, S_6_, Ps_3_, and Pa_2_ sometimes present. Eyes G and H small and subequal. Eye patch with 5 or 6 setae.

*Legs*. Trochanteral organ with triangular setal pattern and up to 34 setae. Unguis with 4 internal teeth; basal teeth located approximately 60% of inner claw length. Unguiculus acuminate with small serrations on internal edge.

*Thorax*. Th. 2 macrosetae a5, m_1_, m_2_, m_4_, m_4i_, m_4pi_, and m_5_ present (Fig. 32B); m_2i_ and m_2i2_ usually present; m_1i_ and m_4p_ sometimes present; all posterior macrosetae (series P) are present. Th. 3 macrosetae a_1_, a_2_, a_3_, a_4_, a_4i_, a_5_, a_6_, a_7_, m_5_, m_5i_, m_6_, and m_7 _present (Fig. 32C); most posterior macrosetae (series P) present, p3 present or absent. Both thoracic segments with a moderate number of supplemental macrosetae in zone Pm (Fig. 32B,C).

*Abdomen*. Abd. 1 with 7-10 macrosetae (Fig. 32D). Abd. 2 macroseta a_2_, a_3_, m_3_, m_3e_, m_3ep_, and m_5_ present; m_3ei_ and m_3ea_ usually absent (Fig. 32E). Abd. 3 macroseta a_1_, m_3_, am_6_, pm_6_, and p_6_ present (Fig. 32F); a_3_ usually absent. Abd. 4 with 7-8 inner macrosetae (Fig. 32G). Mucronal teeth subequal.

###### Remarks.

*Entomobrya nivalis* can be diagnosed by the presence of a U-shaped or “11” shaped pattern on Abd. 4 combined with the presence of macrosetae head S_4i_, Th. 2 m_5_, Abd. 2 m_3ep_, and Abd. 3 a_1_, and the absence of Abd. 3 a_2_ (see Table 2 for additional diagnostic characters). This species has a highly variable color pattern with many intermediate forms that intergrade with *Entomobrya atrocincta* females, *Entomobrya intermedia*, and *Entomobrya multifasciata*. However, *Entomobrya nivalis* can be separated from these species by chaetotaxy (Table 3) and, with careful consideration, color pattern; the presence of a U-shaped or “11” shaped pattern on Abd. 4 is unique to this species. Therefore, it is critical to evaluate chaetotaxy in addition to color pattern when making a species diagnosis.

It is important to note that the large genetic distances between presumably conspecific individuals (Katz et al. 2015; Feng Zhang, personal communication), differences in chaetotaxy between populations in North America and Europe (Jordana 2012), and variable color pattern among populations across its world-wide distribution, suggests that *Entomobrya nivalis* likely represents a cryptic species complex.

###### Distribution.

North America and Europe. Records of *Entomobrya nivalis* in North America are suspect if diagnosed without considering chaetotaxy given the superficial similarities in color form expressed by *Entomobrya atrocincta* females (See Fig. 9G,H). See Suppl. material 2: L for a distribution map and below for a list of material examined with collection and locality information.

###### Material examined.

USA: 2 on slides, 50+ in vial, Maine, Hancock Co., Acadia National Park (44.353823,-68.224754), moss, veg. sweep (blueberry, juniper, populus), 17.viii.2011 (E. C. Bernard), #2009-37; 3 on slides, 5 in vial, Pennsylvania, Allegheny Co., Allegheny National Forest, Dewdrop campground (41.83092,-7895937), leaf litter, 8.vii.2008 (S. M. Shreve); 1 on slide, Vermont, Chittenden Co., Red Rock, Locality I (44.44493,-73.23040), leaf litter, 6.vi.2011 (J. Fisher); 2 on slides, Vermont, Chittenden Co., Farrell Park, Locality II (44.44454,-73.20178), leaf litter, 13.vi.2001 (J. Fisher); 1 on slide, Vermont, Chittenden Co., Farrell Park, Locality II (44.44454,-73.20178), leaf litter, 4.vii.2001 (J. Fisher); 2 on slides, Vermont, Chittenden Co., Farrell Park, Locality II (44.44454,-73.20178), leaf litter, 26.vii.2001 (J. Fisher); 1 on slide, Vermont, Lamoille Co., Stowe (44.48377,-72.69859), leaf litter, 24.vii.2001 (J. Fisher); 1 on slide, Vermont, Lamoille Co. (44.54858,-72.79393), DNA ID#: 12-FSVTlam-ni-1; 1 on slide, Vermont, Washington Co., Locality II, Barre (44.19968,-72.50135), leaf litter, 8.vi.2001 (J. Fisher); 2 on slides, Vermont, Washington Co., Locality II, Barre (44.19968,-72.50135), leaf litter, 24.vii.2001 (J. Fisher); 2 on slides, 6 in vial, Vermont, Rutland Co., Green Mountain National Forest, Greendale Recreation Area (43.35112,-72.82225), leaf litter, 10.vii.2008 (S. M. Shreve); 1 on slide, 6 in vial, Wisconsin, Dodge Co., Horicon Marsh National Wildlife Refuge, end of Dike Rd (43.52736,-88.64381), 12.vi.2011, AK11-47; 2 on slides, 7 in vial, Wisconsin, Sauk Co., Devil’s Lake State Park, 0.5mi down Steinke Basin Loop trail (43.4255,-89.71039), 12.vi.2011, AK11-50.

##### Entomobrya quadrilineata

Taxon classificationAnimaliaCollembolaEntomobryidae

Bueker, 1939

**Figure 33.**
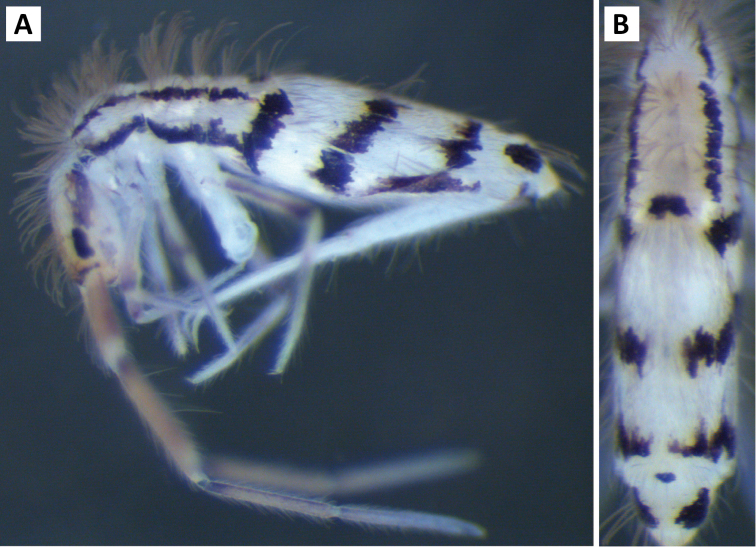
Color pattern of *Entomobrya quadrilineata*. Lateral (**A**) and dorsal (**B**) views of specimen collected from Stewart Co., TN.

**Figure 34.**
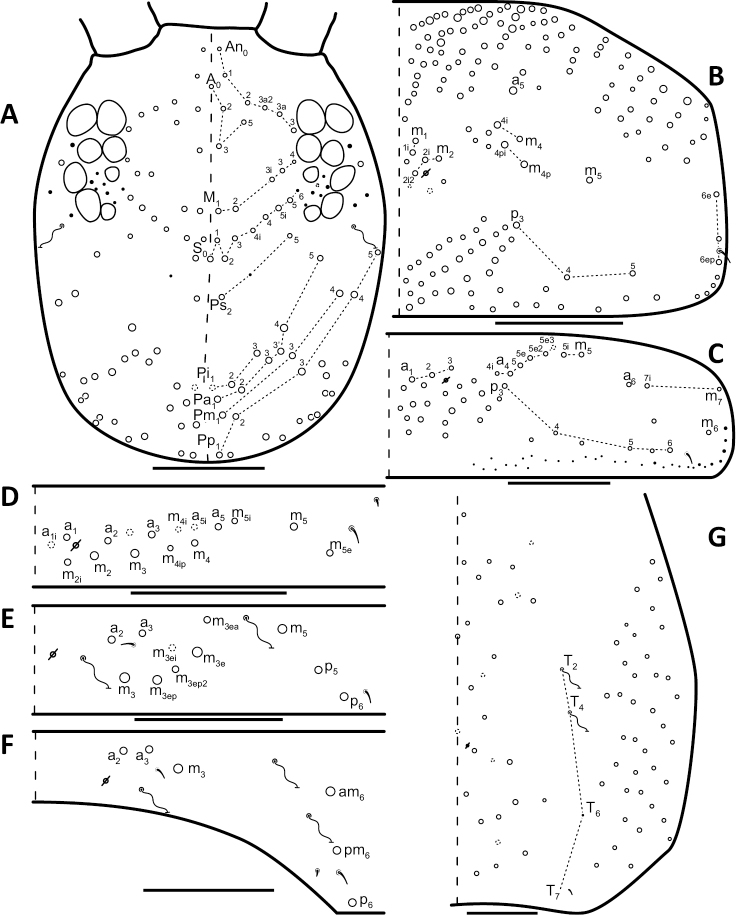
Dorsal chaetotaxy of *Entomobrya quadrilineata*: **A** Head **B** Mesothorax **C** Metathorax **D** 1^st^ abdominal segment **E** 2^nd^ abdominal segment **F** 3^rd^ abdominal segment **G** 4^th^ abdominal segment. Scale bars = 100 µm. See Figure 5 for symbol legend.

Figs 2 , 13C , 33 , 34 , 39

###### Description.

*Body shape and color pattern*. Body very elongate and cylindrical. Color pattern monomorphic (Fig. 33); white or yellow background with black or dark blue or purple pigment forming two dark parallel longitudinal stripes extending from anterior margin of Th. 2 through posterior margin of Abd. 2. Dark pigment present along lateral margins of Th. 2 through Abd. 2, forming 2 additional lateral longitudinal bands. Abd. 3 and Abd. 4. with 2 angled bands. A small patch of dark pigment sometimes occurs medially on Abd. 3. An irregular and (and sometimes incomplete) transverse band present along posterior margin of Abd. 4. Abd. 5 with 2 small lateral patches of pigment sometimes forming 2 triangles. Abd. 6 usually pale, without dark pigment. Apex of femora usually with a patch of dark pigmentation. Antennae usually with some light brown or purple pigment, darkening near apex. Longitudinal bands usually present in juveniles.

*Head*. Apical bulb of 4^th^ antennal segment usually bilobed. Long differentiated smooth setae on ventral side of 1^st ^antennal segment ≈4× short setae. Four prelabral setae ciliate. Ornamentation of the distal margin of the labral papillae with single seta or spine-like projection (Fig. 2). Lateral appendage of labial papilla E almost twice as long as papilla. Labial triangle chaetotaxy slightly irregular and atypical: M1, r, E, L1, L2, all ciliate; r significantly smaller than other setae; a supplementary ciliate seta sometimes present internal to M1, and relatively difficult to observe; A1-A5 smooth. Dorsal head chaetotaxy (Fig. 34A) with macrosetae An’_0_, An_3a3_, A_6_, S’_0_, S_6_, Ps_3_, and Pm_1i_ absent; Pi_1_ sometimes present. Eyes G and H small and subequal. Eye patch with 5 or 6 setae.

*Thorax*. Thoracic chaetotaxy greatly developed, with high levels of variation and many supplemental macrosetae. Th. 2 zone A enlarged and sometimes merging with medial macrosetae forming a single, large patch of setae (Fig. 34B). Th. 3 macrosetae m_4_, m_5p_, a_6i_, and a_7_ absent (Fig. 34C). Both thoracic segments with zone Pm enlarged, with many supplemental macrosetae present forming wing-like patches and extending near anterior row (Fig. 34B,C). Th. 3 pseudopore displaced anteriorly, very close to macrosetae a_1_, a_2_, and a_3 _.

*Legs*. Trochanteral organ with rectangular setal pattern and up to 41 setae.

*Abdomen*. Abdominal chaetotaxy highly developed. Row of microsetae along posterior margin present in all segments (not displayed in figure). Abd. 1 with 12-16 macrosetae (Fig. 34D). Abd. 2 macrosetae a_2_, a_3_, m_3_, m_3e_, m_3ep_, m_3ep2_, m_3ea_, m_5_, p_5_, and p_6_ present (Fig. 34E); m_3ei_ usually present. Abd. 3 macrosetae a_2_, a_3_, m_3_, am_6_, pm_6_, and p_6_ present (Fig. 34F); a_1_ absent. Abd. 4 elongate, with at least 25 macrosetae internal to bothriotricha T_2_ and T_4_ (Fig. 34G), but number of macrosetae extremely variable between individuals and even within single individuals (Fig. 16C). Pseudopores on Abd. 4 with asymmetric relative insertions with respect to macroseta and bothriotricha (Fig. 13C). Mucronal teeth subequal.

###### Remarks.

*Entomobrya quadrilineata* can be identified by the presence of 2 parallel longitudinal thoracic stripes combined with the presence of head macrosetae M_3i_ and ps_5 _and the absence of Abd. 3 macroseta a_1_ (see Table 2 for additional diagnostic characters). This species is part of the *Entomobrya bicolor* complex. Many supplemental macrosetae and extreme setal variation make it difficult to separate species in this group using chaetotaxy and other traditional morphological characters. However, there are clear differences in color pattern which can be used to differentiate *Entomobrya quadrilineata* from *Entomobrya decemfasciata* and *Entomobrya bicolor* (Table 5); *Entomobrya quadrilineata* has a relatively stable and easily distinguishable color pattern and can be diagnosed by the presence of two parallel longitudinal stripes extending from the anterior margin of Th. 2 through the posterior margin of Abd. 2 (Fig. 33). See remarks for *Entomobrya decemfasciata* for more information regarding species diagnosis and delimitation. Also, see Suppl. material 3: A,B for photographs of the type specimen.

###### Distribution.

Endemic to North America (Suppl. material 2: M). Many records may be misidentifications due to similarity in color pattern and chaetotaxy with *Entomobrya decemfasciata*. Records of *Entomobrya quadrilineata* without thoracic stripes are most likely *Entomobrya decemfasciata*.

###### Material examined.

USA: *Neo-holotype* (Christiansen 1951), 1 on slide, Fountain Bluff, Ill. 5-15-32, Coll. Ross + Mohr, INHS Cat. No. 529,188; *Neo-paratype* (Christiansen 1951), 1 on slide, Fountain Bluff, Ill. 5-15-32, Coll. Ross + Mohr, INHS Cat. No. 529,189; *Neo-paratype* (Christiansen 1951), 1 in vial, Fountain Bluff, Ill. 5-15-32, Coll. Ross + Mohr; 4 on slides, 13 in vial, Illinois, Monroe Co., Kidd Lake Marsh State Natural Area (37.97211,-89.80135), leaf litter #42, 29.ix.2009 (S. Taylor & F. Soto-Adames), sjt09-114; 1 in vial, Illinois, Pope Co., Bell Smith Springs (37.51882,-88.65782), bare sandstone bedrock above canyon in mosses and lichens, 28.viii.2011 (J. Cech); 1 on slide, Illinois, Vermilion Co., Kennekuk Cove County Park, Windfall Prairie Nature Preserve (40.20995,-87.74181), vacuum hill prairie, 16.vi.2011, AK11-59a; 1 in vial, Illinois, Wayne Co., County Road 580N (38.34122,-88.23992), leaf litter from north side of road, 15.vii.2011 (A. Katz & F. Soto-Adames), AK11-61; 4 in vial, Illinois, Wayne Co. (38.32500,-88.25016), 15.vii.2011 (A. Katz & F. Soto-Adames), AK11-63; 1 in vial, Tennessee, Stewart Co., Land Between the Lakes National Recreation Area,. 25mi down Neville Bay Rd. (36.60757,-87.93457), leaf litter on forest floor, 31.v.2011, AK11-44; 1 on slide, 2 in vial, Tennessee, Stewart Co., Land Between the Lakes National Recreation Area, Fox Ridge Rd (36.66392,-87.98596), leaf litter, 7.viii.2011, AK11-105.

##### Entomobrya unifasciata

Taxon classificationAnimaliaCollembolaEntomobryidae

Katz & Soto-Adames sp. n.

http://zoobank.org/A68AD8F0-1545-471C-812D-2F066A08858F

**Figure 35.**
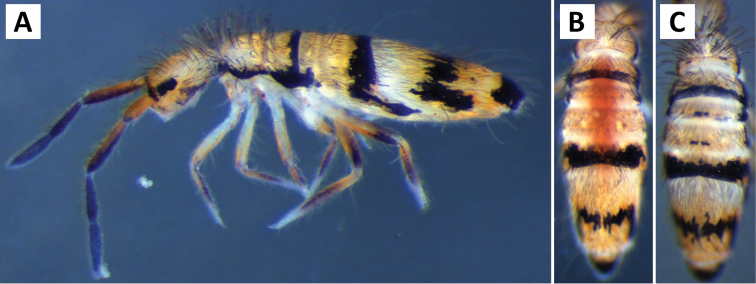
Color pattern of *Entomobrya unifasciata* sp. n. collected from: **A** Sevier Co., TN **B** Union Co, GA **C** Laurel Co., KY.

**Figure 36.**
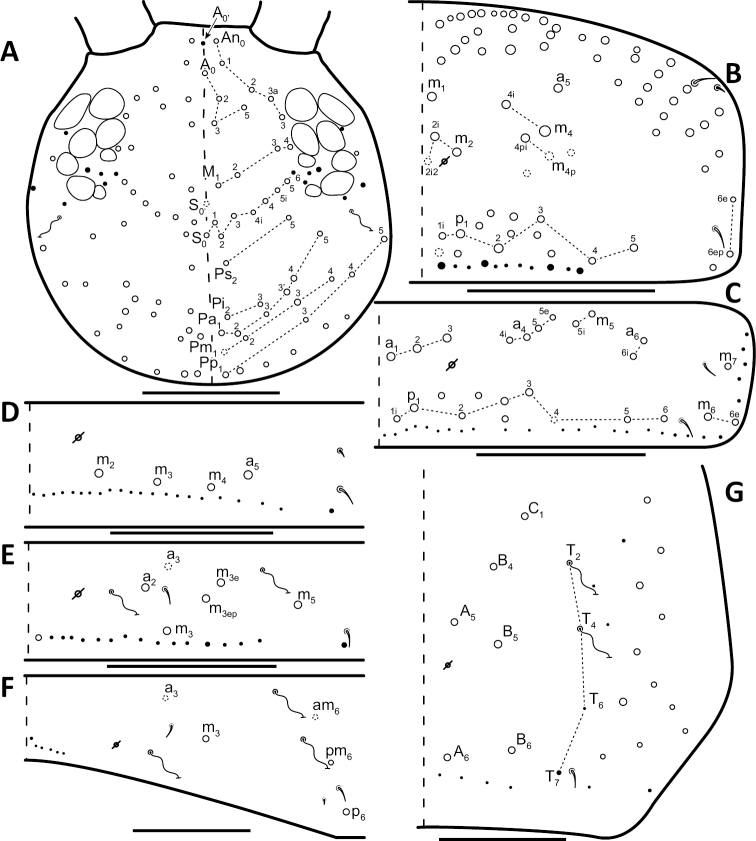
Dorsal chaetotaxy of *Entomobrya unifasciata* sp. n.: **A** Head **B** Mesothorax **C** Metathorax **D** 1^st^ abdominal segment **E** 2^nd^ abdominal segment **F** 3^rd^ abdominal segment **G** 4^th^ abdominal segment. Scale bars = 100 µm. See Figure 5 for symbol legend.

Figs 2 , 16D , 35 , 36 , 39

###### Etymology.

 From the Latin words *uno* and *fasciatus*, which translates to “one band”. This species has only one band found along the posterior margin of the metathorax, a character that distinguishes it from *Entomobrya ligata*, which has two bands; one along each posterior margin of the meta- and mesothorax.

###### Type material.

*Holotype*, ♂, USA: Kentucky, Laurel County, Levi Jackson State Park (37.08247,-84.04528), leaf litter collected at night, 28.v.2011, AK11-37.

*Paratypes*, USA: 2 on slides, 1 in vial, Georgia, Union Co., Brasstown Bald Rd., tiny water trickle near road surrounded by dryish leaves (34.86040,-83.80193), leaf litter, 26.v.2011 (E. C. Bernard) #2011-28; 1 on slide, Kentucky, Laurel Co., Levi Jackson State Park (37.08247,-84.04528), leaf litter collected at night, 28.v.2011, AK11-37; 1 on slide, 2 in vial, North Carolina, Henderson Co., Blue Ridge Parkway, Mill River Overlook (35.4482,-82.71963), under bark on logs, 4.vi.2007 (E. C. Bernard), 07031EB; 10 in vial, North Carolina, Swain Co., Great Smoky Mountains National Park, Balsom Mountain, Heintooga Ridge Rd. (35.57030,-83.16917), leaf litter along road, 29.v.2011, AK11-38; 1 on slide, 35 in vial, North Carolina, Swain Co., Great Smoky Mountains National Park, Balsom Mountain, Heintooga Ridge Rd. (35.57030,-83.16917), under bark, 29.v.2011, AK11-39;11 in vial, North Carolina, Swain Co., Great Smoky Mountains National Park, Balsom Mountain, Heintooga Ridge Rd. (35.57030,-83.16917), leaf litter by river, 29.v.2011, AK11-40; 1 on slide, 1 in vial, Tennessee, Sevier Co., Great Smoky Mountains National Park, 1 mi down greenbrier Rd. (35.72640,-83.40173), leaf litter by stream, 30.v.2011, AK11-41; 1 in vial, Tennessee, Sevier Co., Great Smoky Mountains National Park, 1 mi down greenbrier Rd. (35.72640,-83.40173), leaf litter stuck in nook of tree, 30.v.2011, AK11-42.

###### Description.

*Body shape and color pattern*. Body oval and cylindrical. Length up to 1.85 mm. Color pattern stable (Fig. 35), always with 3 transverse bands, 1 thin regular band along posterior margin of Th. 3, an irregular, patchy, thick band covering most of Abd. 3, and a highly irregular and sometimes broken band across medial section of Abd. 4. A small patch of pigment covers Abd. 5 and 6. Patterns usually consisting of black or dark blue pigment on a yellow background. Dark pigment usually occurring in patches along lateral margins of Th. 2 through Abd. 4. Small, rectangular black patches may occur in pairs on posterior margin of Th. 2, Abd. 1, and Abd. 2. A faint transverse band sometimes on posterior margin of Th. 2, but if present, always much lower in opacity compared to transverse band along posterior margin of Th. 3. Antennae with purple pigment, darker near apex. Legs usually white, with small purple patches on apical end of femora. A medial ring of purple pigment also occurs on tibiotarsus of hind legs.

*Head*. Apical bulb of 4^th^ antennal segment usually bilobed, sometimes simple. Long differentiated smooth setae on ventral side of 1^st ^antennal segment ≈3× short setae. Four prelabral setae finely ciliate, seemingly smooth at low magnification under light microscopy. Ornamentation of the distal margin of the labral papillae with single seta or spine-like projection (Fig. 2). Lateral appendage of labial papilla E short, extending only ¾ papilla length. Dorsal head chaetotaxy (Fig. 36A) with macrosetae A_6_, M_3i_, S_6_, Ps_3_, Pi_1_, Pm_1i_, and Pp_2_, absent; S’_0_ usually absent, but observed in 2 individuals; An’_0_, a short mesoseta present medially between both An_0_. Eyes G and H small and subequal. Eye patch with 3 setae.

*Thorax*. Th. 2 macrosetae a5, m_1_, m_2_, m_4_, m_4pi_, and m_4i_ present (Fig. 36B). Macrosetae m_2i2_, m_4p_, and m_5_ sometimes present; 2 additional macrosetae sometimes present on either side of m_4p_. Th. 3 macrosetae a_1_, a_2_, a_3_, a_4_, a_4i_, a_5_, a_5e_, a_6_, a_6i_, m_5_, m_5i_, m_6_, m_6e_, and m_7 _present (Fig. 36C); most posterior (series P) macrosetae present, p_4_ present or absent. Both thoracic segments with moderate number of supplemental macrosetae on zone Pm (Fig. 36B,C).

*Legs*. Trochanteral organ with triangular setal pattern and up to 22 setae. Unguis with 4 internal teeth; basal teeth located approximately middle of inner claw length (Fig. 16D). Unguiculus acuminate with small serrations on internal edge.

*Abdomen*. Abdominal chaetotaxy reduced and slightly variable. Abd. 1 with 4 macrosetae: a_5_, m_2_, m_3_, and m_4_ (Fig. 36D). Abd. 2 with 5 macrosetae: a_2_, m_3_, m_3e_, m_3ep_, and m_5_ (Fig. 36E); a_3_ usually absent. Abd. 3 with 3 macrosetae: m_3_, pm_6_, and p_6_ (Fig. 36F); a_3_ and am_6_ usually absent. Abd. 4 with 6 inner macrosetae (Fig. 36G).

###### Remarks.

*Entomobrya unifasciata* sp. n. can be diagnosed by the presence of only three dark transverse bands (no band across the posterior margin of Th. 2), presence of head mesoseta An’_0_, 4 macrosetae on Ab. 1, and 3 eye patch setae (see Table 2 for additional diagnostic characters). This species is part of the *Entomobrya ligata* complex (see remarks for *Entomobrya ligata*) and is very similar to *Entomobrya ligata* and *Entomobrya neotenica* sp. n., but can be separated by characters outlined in Table 6. Though identical in chaetotaxy, molecular evidence (Katz et al. 2015) and the absence of a dark transverse band on the posterior margin of Th. 2 separate this species from *Entomobrya ligata*.

###### Distribution.

Endemic to North America (Suppl. material 2: N). Many records of *Entomobrya ligata*, especially those collected from the Smokey Mountain region west to the Mississippi River, are likely to be *Entomobrya unifasciata* sp. n.

##### Entomobrya unostrigata

Taxon classificationAnimaliaCollembolaEntomobryidae

Stach, 1930

**Figure 37.**
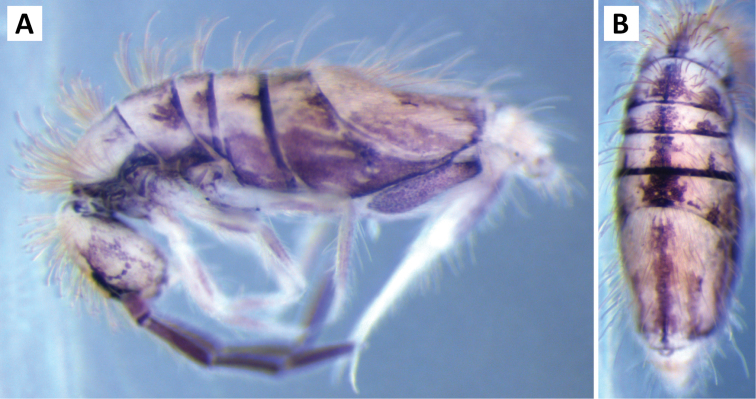
Color patterns of *Entomobrya unostrigata*. Lateral (**A**) and dorsal (**B**) views of specimen collected from Chittenden Co., VT.

**Figure 38.**
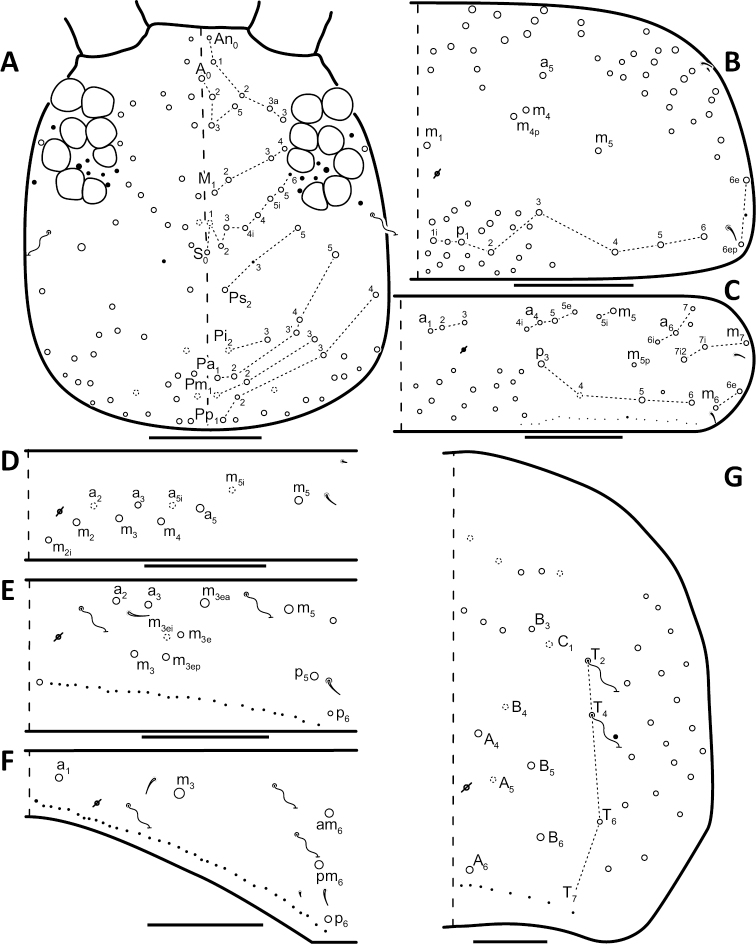
Dorsal chaetotaxy of *Entomobrya unostrigata*: **A** Head **B** Mesothorax **C** Metathorax **D** 1^st^ abdominal segment **E** 2^nd^ abdominal segment **F** 3^rd^ abdominal segment **G** 4^th^ abdominal segment. Scale bars = 100µm. See Figure 5 for symbol legend.

**Figure 39.**
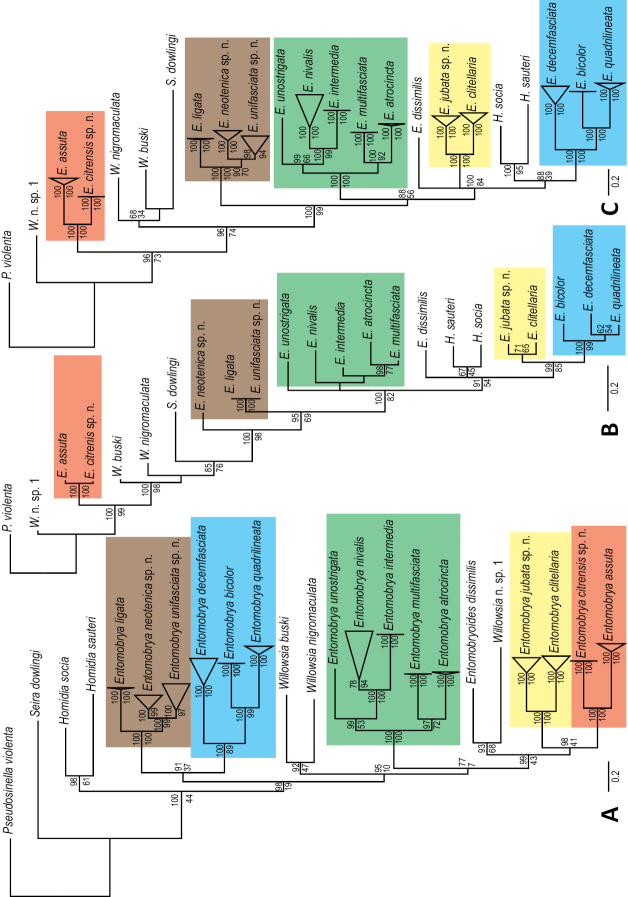
Bayesian 50% majority rules consensus trees with *Pseudosinella violenta* as the outgroup: **A** phylogeny resulting from the analysis of cytochrome *c* oxidase I DNA sequences (complete gene, 1539 bp) from Katz et al. (2015) **B** phylogeny resulting from the analysis of 179 morphological characters **C** combined phylogeny (COI and morphology). *Entomobrya* species groups are highlighted with colored boxes. Branches of representing taxa from the same species were collapsed into triangles (triangle length represents branch lengths between collapsed branches) for simplicity. Branch labels include posterior probabilities (above) and maximum likelihood bootstrap support (below). Scale bars represent 0.2 base substitutions.

Figs 2 , 3C , 37 , 38 , 39

###### Description.

*Body shape and color pattern*. Body relatively robust and cylindrical. Color form largely monomorphic (Fig. 37): white, pale green or yellow background with purple or black pigment forming a thin medial longitudinal stripe from anterior margin of Th. 2 to posterior margin of Abd. 5; band ostensibly thicker on Th. 3 through Abd. 3. Patches of pigment forming a lateral line along margins of Th. 2 through Abd. 5, with variable spots of pigment present throughout. Abd. 5 and Abd. 6 usually lack dark pigment. Legs and furcula white and/or with light purple pigment. Antennae with light purple pigment.

*Head*. Apical bulb of 4^th^ antennal segment usually bilobed. Long differentiated smooth setae on ventral side of 1^st ^antennal segment ≈3× short setae. Four prelabral setae ciliate. Ornamentation of the distal margin of the labral papillae with 2 seta or spine-like projections (Fig. 2). Lateral appendage of labial papilla E extending just past tip of papilla. Dorsal head chaetotaxy (Fig. 38A) with macrosetae An’_0_, A_3a2_, A_3a3_, A_6_, M_3i_, S’_0_, S_6_, Ps_3_, Pi_1_, Pa_3_, and Pm_1i_ absent; S_1_, Pi_2_, and Pm_1_ usually present. Eyes G and H enlarged and similar in size to eyes C-F. Eye patch with 5 setae.

*Thorax*. Thoracic chaetotaxy well-developed and relatively stable. Th. 2 macrosetae a_5_, m_1_, m_4_, m_4p_, and m_5_ present (Fig. 38B); posterior macrosetae (series P) present. Th. 3 macrosetae a_5e2_, a_5e3_, m_4_, m_5p_, and a_7 _absent (Fig. 38C); P_4_ usually present. Both thoracic segments with many supplemental macrosetae on zone Pm (Figs 3C; 38B,C).

*Legs*. Trochanteral organ with triangular setal pattern and up to 21 setae.

*Abdomen*. Abdominal chaetotaxy highly developed and variable. Abd. 1 with 7-10 macrosetae (Fig. 38D). Abd. 2 macroseta a_2_, a_3_, m_3_, m_3ep_, m_3e_, m_3ea_, and m_5_ present (Fig. 38E); m_3ei_ usually present. Abd. 3 macroseta a_1_, m_3_, am_6_, pm_6_, and p_6_ present (Fig. 38F). Abd.4 with 10-16 macrosetae present interior to bothriotricha T_2_ and T_4_ (Fig. 38G). Anterior half of Abd. 4 usually with 1 or 2 conspicuous rows of macrosetae. Apical mucronal tooth enlarged.

###### Remarks.

*Entomobrya unostrigata* can be easily diagnosed by color pattern, a thin medial longitudinal stripe from the anterior margin of Th. 2 to the posterior margin of Abd. 5, combined with the absence of macrosetae Th. 2 m_2_ and Abd. 3 a_2 _and the presence of macrosetae m_5_ on Th. 2 and a_1 _on Abd. 3 (see Table 2 for additional diagnostic characters). This species exhibits atypical morphology for a Nearctic *Entomobrya*. Eyes G and H are greatly enlarged and similar in size to eyes C-F, an uncommon trait unobserved in other Nearctic *Entomobrya*. Additionally, macrosetae usually form 2 rows on the anterior half of Abd. 4; a pattern shared with many Palearctic *Entomobrya* and also with members in the genus *Homidia*, but not with other *Entomobrya* treated here. *Entomobrya unostrigata* is a recently introduced species, now with a widespread Nearctic distribution (Christiansen and Bellinger 1998), which may explain its distinction from other North American *Entomobrya* species included in the present study. The *Entomobrya unostrigata* specimens observed for this study have only two large seta or spine-like projections on each labral papillae (Fig. 2). However, multiple variations of the labral papillae have been reported (Christiansen 1958b; Christiansen and Bellinger 1992; Christiansen and Bellinger 1998; Jordana 2012).

###### Distribution.

Nearctic, Palearctic, and Australia (Suppl. material 2: O).

###### Material examined.

USA: 2 on slides, Vermont, Chittenden Co., South Burlington, Vegetable garden on south side of Swift St. at intersection with Spear St. (44.4433,-73.1893), 3.viii.2003 (F. Soto-Adames); 1 on slide, Wisconsin, Dodge Co., Horicon Marsh National Wildlife Refuge, end of Dike Rd. (43.52736,-88.64381), 12.vi.2011, AK11-47.

### Morphological phylogeny

Bayesian analysis of 179 morphological characters from 23 taxa produced a single consensus tree with high support (Fig. 39B). The most likely tree inferred by the maximum likelihood analysis is congruent with the Bayesian tree and bootstrap values were added to the Bayesian consensus tree (Fig. 39B). When *Pseudosinella violenta* is designated as the outgroup, the topology of the tree assumes a ladder-like evolutionary progression that is associated with an increase in the number of dorsal macrosetae. *Entomobrya assuta* and *Entomobrya citrensis* sp. n., the two species with the smallest number of macrosetae, form a monophyletic group at the base of the tree, whereas *Entomobrya bicolor*, *Entomobrya decemfasciata*, and *Entomobrya quadrilineata*, which are characterized by an abundance of dorsal macrosetae, form the most derived monophyletic group. This tree indicates that *Entomobrya* is paraphyletic, as the genera *Seira*, *Homidia*, *Entomobryoides*, and most representatives of *Willowsia* are interspersed among clades of *Entomobrya* species. The two species in the genus *Homidia* form a monophyletic group, but the genus *Willowsia* is not monophyletic. Other clades identified by the molecular COI analysis in Katz et al. (2015) (Fig. 39A) were not resolved as monophyletic groups based on morphological characters.

### Combined phylogeny (COI and morphology)

Bayesian analysis based on combined morphology and COI (Katz et al. 2015) datasets yielded a highly supported tree (Fig. 39C). The most likely tree inferred by the maximum likelihood analysis is congruent with the Bayesian tree and bootstrap values were added to the Bayesian consensus tree (Fig. 39C). The addition of molecular characters resolves all monophyletic species groups/clades of *Entomobrya* observed in the COI only tree (Fig. 39A). However, the deeper relationships among clades follow the progressive evolution towards an increased number of macrosetae supported by the morphological analysis. Both analyses (morphology only and combined) indicate that *Entomobrya* is paraphyletic.

## Discussion

### Diagnostic and phylogenetic utility of chaetotaxy

Detailed examination of the adult chaetotaxy of 15 species of North American *Entomobrya* suggests that the exclusive use of chaetotaxy for species diagnosis and as phylogenetic characters can potentially cause serious confusion. This study uncovered high levels of previously undocumented intraspecific variation and asymmetry of dorsal macrosetae (Fig. 13), and has made it clear that examination of many individuals is critical to properly identify variable chaetotaxy in order to choose appropriate characters for species delimitation. For example, Christiansen and Bellinger (1998) separate many species based on the chaetotaxy of Abd. 2 and Abd. 3, but in some species groups both regions contain significant intraspecific variation. In the Collembola of North America (Christiansen and Bellinger 1998), species within the *nivalis* group (*Entomobrya nivalis*, *Entomobrya atrocincta*, *Entomobrya multifasciata*, and *Entomobrya intermedia*) are separated by a combination of color pattern and by the presence or absence of four macrosetae: m_3ep_ on Abd. 2 and a_1_, a_2_, and a_3_ on Abd. 3. This study shows that these characters must be used in combination to provide sufficient separation of these species due to overlapping intergrades of color forms and the presence macrosetae polymorphisms. Species in the *bicolor* group (*Entomobrya bicolor*, *Entomobrya decemfasciata*, and *Entomobrya quadrilineata*) present the most obvious example of the failure of chaetotaxy to provide an adequate means for species separation. Large numbers of macrosetae, extreme levels of inter-individual variation, and the common occurrence of asymmetries, cause significant overlap and obscure the homology of macrosetae otherwise considered important in species separation. The present study shows that color pattern is absolutely critical for the identification of species within this group.

These problems are cause for concern considering the important role of dorsal chaetotaxy in diagnosis and delimitation of species in the family Entomobryidae. Many studies are based on chaetotaxy as primary (or sole) evidence for species separation, following Szeptycki’s (1979) nomenclatural system (Chen and Christiansen 1993; Jordana 2012). The homology of macrosetae as defined by Szeptycki (1979) assumes that a fixed number of macrosetae occur in strict, predefined positions rather than randomly distributed within a given area. Homologies are easier to determine between species in genera characterized by small numbers of macrosetae, and chaetotaxy likely provides accurate phylogenetic estimation in these groups. However, in groups such as *Entomobrya*, intraspecific variation, differences in setae arrangements, asymmetries, and large numbers of supplemental setae, render homology assessment a subjective and arbitrary process (Potapov and Kremenista 2008). Incorrect homology assignments obscure any useful phylogenetic information provided by chaetotaxy.

Post-embryonic studies that test Szeptycki’s (1979) hypotheses have been successful in identifying and refining setae homologies in some pluri- and polychaetotic species and groups (Soto-Adames 2008; Zhang et al. 2011), but these studies are laborious and are only conclusive for species and/or groups examined. Information regarding the post-embryonic development of *Entomobrya* chaetotaxy is very limited and mainly concerned with Palearctic species (Szeptycki 1979). Explicit hypotheses concerning the underlying molecular mechanisms governing macrosetae development and position, to our knowledge, have only been tested in Diptera. These mechanisms are generally very complex and highly regulated processes, controlled by multiple genes that ensure macrosetae do occur in strictly defined locations (Simpson 1990; Heitzler et al. 1996; Leyns et al. 1996; Simpson et al. 1999; Furman and Bukharina 2008). However, it is uncertain (and unlikely) that Collembola macrosetae, and/or the genes that regulate them, are homologous to those of Diptera.

The phylogenies based on morphological and molecular data illustrate the effects that chaetotaxy has on phylogeny estimation. The trend of progressive evolution towards an increased number of macrosetae presented in the morphology and combined phylogenies is suspicious and may be driven by outgroup choice and character coding strategy. *Entomobrya assuta* and *Entomobrya citrensis* sp. n., sister species characterized by reduced chaetotaxy, are basal to all other *Entomobrya*, together with all scaled species included in the analysis (*Pseudosinella*, *Willowsia*, and *Seira*), which also have few macrosetae. This association is not reflected in the analysis of COI alone (Katz et al. 2015; Fig. 39A) and is likely due to the accumulation of characters (each seta in a multiplet was defined and scored as a separate character, see Suppl. material 1) present in polychaetotic species but absent in the *Entomobrya assuta* group and the outgroup. Evaluation of coding strategies for chaetotaxy, and the effect of outgroup choice is clearly needed to exact the maximum amount of phylogenetic information while minimizing homoplasy. Despite the conflict in branching pattern for deep nodes estimated by morphology and COI, the combined analysis retains the species composition and relationship of the five species groups identified by the COI analysis. These relationships are quite obvious and were proposed by Christiansen (1958b) before modern sequencing and dorsal chaetotaxy systems were available.

### Phylogenetic relationships within Entomobryini and *Willowsia*

The result of the combined phylogenetic analysis (COI and morphology) of 15 species of North American *Entomobrya* in addition to 6 species representing 3 closely related genera (*Entomobryoides*, *Homidia* and *Willowsia*) and 2 outgroup species (*Pseudosinella violenta* and *Seira dowlingi*), generated a highly supported phylogeny of the North American Entomobryini and *Willowsia*. Several interesting results were observed concerning relationships among some currently recognized *Entomobrya* species. *Entomobrya nivalis*, *Entomobrya intermedia*, *Entomobrya multifasciata*, and *Entomobrya atrocincta* were resolved as a monophyletic clade; all are closely related but distinct species diagnosable using morphology and COI sequences. Overlapping intraspecific variation in color pattern and chaetotaxy has caused many to consider these species synonymous (See Christiansen 1958b and Jordana 2012 for revision history). This study has, for the first time, combined substantial molecular (Katz et al. 2015) and morphological evidence in support of their separation. It is also worth noting that these were the only *Entomobrya* species included in the analysis with Holarctic distribution, all commonly reported throughout North America (Christiansen and Bellinger 1998) and Europe (Jordana 2012). The monophyly of the group and separation from species endemic to North America suggest a common origin in Europe or Palearctic region. It is possible that the group is either part of a shared, relictual Laurasian fauna or that the species were introduced from Europe in historical times. Evaluation of these two hypotheses will require molecular analysis of extensive samples of North American and European populations.

The resulting phylogeny also raises questions regarding the generic relationships within the tribe Entomobryini (here represented by the genera *Entomobrya*, *Entomobryoides* and *Homidia*) and *Willowsia*, a closely related genus. Research concerning the systematics of these groups is limited, and most phylogenetic studies have focused on suprageneric relationships by utilizing morphology, allozymes, and/or ribosomal markers that provided limited resolution and support of generic relationships (Lee and Park 1991; Lee et al. 1995; D’Haese 2002; Xiong et al. 2008). However, more recent work by Zhang et al. (2014b) indicated the non-monophyly of Entomobryini and Willowsiini based on molecular phylogeny. Zhang et al. (2014a) also questioned the monophyly of Willowsiini. Further analyses of S-chaetae (dorsal sensilla) dismissed Willowsiini as monophyletic (Zhang and Deharveng 2015).

This study supports Zhang et al.’s (2014a, 2014b) and Zhang and Deharveng’s (2015) findings regarding the non-monophyly of Entomobryini and Willowsiini. Our phylogenetic analysis places both *Willowsia nigromaculata* (Lubbock) and *Willowsia buski* (Lubbock) within the *Entomobrya* clade, further supporting the paraphyly of *Entomobrya*. This is not entirely unexpected considering the means by which these genera are differentiated. The genus *Entomobrya* is considered the most morphologically generalized group of Entomobryinae without distinct apomorphies while morphologically similar species with autapomorphic characters are separated into different genera (e.g., *Willowsia* with scales, *Entomobryoides* with smooth tibiotarsal setae and *Homidia* with dental spines). In light of the relationships inferred from the phylogeny, the classification of *Entomobrya*, along with some other genera within Entomobryidae, may need to be reevaluated in the future.

Results also indicate that the genus *Willowsia* may be polyphyletic. A new *Willowsia* species (*Willowsia* n. sp. 1) collected in Citrus Co., Florida, is resolved as a sister species to the *Entomobrya* clade, while *Willowsia buski* and *Willowsia nigromaculata* seem to have evolved from lineages within the *Entomobrya* clade. Considering their world-wide distribution, presumably spread by humans, and their similarity to Asian species (Zhang et al. 2011), *Willowsia buski* and *Willowsia nigromaculata* most likely were introduced into North America from Asia. However, *Willowsia* n. sp. 1 shares characteristics (i.e., scale type; see Zhang et al. 2011) with *Willowsia mexicana* Zhang, Palacios-Vargas & Chen, the only other *Willowsia* species known to be endemic to North America (Zhang et al. 2007). *Willowsia* n. sp. 1’s morphological similarity to *Willowsia mexicana* may support an independent origin of New World *Willowsia*. Further exploration by utilizing additional markers and more complete taxon-sampling among closely related genera is needed in order to establish appropriate generic relationships and classifications.

## Supplementary Material

XML Treatment for Entomobrya assuta

XML Treatment for Entomobrya atrocincta

XML Treatment for Entomobrya bicolor

XML Treatment for Entomobrya citrensis

XML Treatment for Entomobrya clitellaria

XML Treatment for Entomobrya decemfasciata

XML Treatment for Entomobrya intermedia

XML Treatment for Entomobrya jubata

XML Treatment for Entomobrya ligata

XML Treatment for Entomobrya multifasciata

XML Treatment for Entomobrya neotenica

XML Treatment for Entomobrya nivalis

XML Treatment for Entomobrya quadrilineata

XML Treatment for Entomobrya unifasciata

XML Treatment for Entomobrya unostrigata

## Supplementary materials

Supplementary material 1 Morphology matrix Data type: Nexus file optimized for Mesquite Explanation note: Alignment nexus file for all morphological characters used for phylogentic analysis. This file is optimized for viewing in mesquite and includes all character state information. This dataset is made available under the Open Database License (http://opendatacommons.org/licenses/odbl/1.0/). The Open Database License (ODbL) is a license agreement intended to allow users to freely share, modify, and use this Dataset while maintaining this same freedom for others, provided that the original source and author(s) are credited.Aron D. Katz, Rosanna Giordano, Felipe Soto-Adames

Supplementary material 2 Distribution maps Data type: PDF file Explanation note: Distribution maps: **A**, *Entomobrya assuta*; **B**, *Entomobrya atrocincta*; **C**, *Entomobrya bicolor*; **D**, *Entomobrya citrensis* sp. n.; **E**, *Entomobrya clitellaria*; **F**, *Entomobrya decemfasciata*; **G**, *Entomobrya intermedia*; **H**, *Entomobrya jubata* sp. n.; **I**, *Entomobrya ligata*; **J**, *Entomobrya multifasciata*; **K**, *Entomobrya neotenica* sp. n.; **L**, *Entomobrya nivalis*; **M**, *Entomobrya quadrilineata*; **N**, *Entomobrya unifasciata* sp. n.; **O**, *Entomobrya unostrigata*. This dataset is made available under the Open Database License (http://opendatacommons.org/licenses/odbl/1.0/). The Open Database License (ODbL) is a license agreement intended to allow users to freely share, modify, and use this Dataset while maintaining this same freedom for others, provided that the original source and author(s) are credited.Aron D. Katz, Rosanna Giordano, Felipe Soto-Adames

Supplementary material 3 Photographs of type specimens Data type: PDF file Explanation note: Photographs of type specimens stored at the Illinois Natural History Survey, Champaign, IL. Lateral (**A**) and dorsal (**B**) views of a neo-paratype specimen of *Entomobrya quadrilineata* Bueker, 1939, Fountain Bluff, IL., V.15.1932, Ross & Mohr. Lateral view of a co- type specimen of *Entomobrya decemfasciata* Packard 1873 (**C**), no locality information. *Entomobrya ligata* Folsom 1924: Co-type specimens, Roorheesville?, NY 30Aug1923, M. J. Leonard (**D, E, F**); Co-type specimen, NY, July 1923, A. Wolf (**G**). This dataset is made available under the Open Database License (http://opendatacommons.org/licenses/odbl/1.0/). The Open Database License (ODbL) is a license agreement intended to allow users to freely share, modify, and use this Dataset while maintaining this same freedom for others, provided that the original source and author(s) are credited.Aron D. Katz, Rosanna Giordano, Felipe Soto-Adames

## References

[B1] Bonet F (1934) Colémbolos de la República Argentina. EOS 9: 123–194.

[B2] Brook G (1883) A revision of the genus *Entomobrya*, Rond. (Degeeria, Nic.). Journal of the Linnean Society of London (Zoology) 17: 270–283. doi: 10.1111/j.1096-3642.1884.tb02023.x

[B3] Bueker ED (1939) Springtails (Collembola) of the St. Louis Area. Transactions of the Academy of Science of Saint Louis 30: 3–30.

[B4] Burkhardt U Filser J (2005) Molecular evidence for a fourth species within the *Isotoma viridis* group (Insecta, Collembola). Zoologica Scripta 34: 177–185. doi: 10.1111/j.1463-6409.2005.00181.x

[B5] Carapelli A Fanciulli PP Frati F Dallai R (1995) The use of genetic markers for the diagnosis of sibling species in the genus *Isotomurus* (Insecta, Collembola). Italian Journal of Zoology 62: 71–76. doi: 10.1080/11250009509356053

[B6] Carapelli A Frati F Fanciulli PP Dallai R (2001) Taxonomic revision of 14 south-western European species of *Isotomurus* (Collembola, Isotomidae), with description of four new species and the designation of the neotype for *I. palustris*. Zoologica Scripta 30: 115–143. doi: 10.1046/j.1463-6409.2001.00055.x

[B7] Carapelli A Frati F Fanciulli PP Nardi F Dallai R (2005) Assessing species boundaries and evolutionary relationships in a group of south-western European species of *Isotomurus* (Collembola, Isotomidae) using allozyme data. Zoologica Scripta 34: 71–79. doi: 10.1111/j.1463-6409.2005.00174.x

[B8] Chen J-X Christiansen K (1993) The genus *Sinella* with special reference to *Sinella* *s.s.* (Collembola: Entomobryidae) of China. Oriental Insects 27: 1–54. doi: 10.1080/00305316.1993.10432236

[B9] Chen J-X Christiansen K (1997) Subgenus *Coecobrya* of the genus *Sinella* (Collembola: Entomobryidae) with special reference to the species of China. Annals of the Entomological Society of America 90: 1–19. doi: 10.1093/aesa/90.1.1

[B10] Christiansen K (1954) Ratios as a means of specific differentiation in Collembola. Entomological News 65: 177–178.

[B11] Christiansen K (1958a) The Entomobryiform male genital plate. Proceedings of the Iowa Academy of Science 65: 474–476.

[B12] Christiansen K (1958b) The Nearctic members of the genus *Entomobrya* (Collembola). Bulletin of the Museum of Comparative Zoology 118: 439–594.

[B13] Christiansen K Bellinger P (1980) The Collembola of North America north of the Rio Grande; A taxonomic analysis. Grinnell College, Grinnell, IA.

[B14] Christiansen K Bellinger P (1992) Insects of Hawaii: A manual of the Insects of the Hawaiian Islands, including an enumeration of the species and notes on their origin, distribution, hosts, parasites, etc. Volume 15. Collembola. University of Hawaii Press, Honolulu, 445 pp.

[B15] Christiansen K Bellinger P (1998) The Collembola of North America north of the Rio Grande; A taxonomic analysis. 2nd Edition Grinnell College, Grinnell, IA.

[B16] Cicconardi F Fanciulli PP Emerson BC (2013) Collembola, the biological species concept and the underestimation of global species richness. Molecular ecology 22: 5382–5396. doi: 10.1111/mec.12472 24112308 10.1111/mec.12472

[B17] Cicconardi F Nardi F Emerson BC Frati F Fanciulli PP (2010) Deep phylogeographic divisions and long-term persistence of forest invertebrates (Hexapoda: Collembola) in the North-Western Mediterranean basin. Molecular Ecology 19: 386–400. doi: 10.1111/j.1365-294X.2009.04457.x 20015142 10.1111/j.1365-294X.2009.04457.x

[B18] D’Haese CA (2002) Were the first springtails semi-aquatic? A phylogenetic approach by means of 28S rDNA and optimization alignment. Proceedings of the Royal Society of London. Series B, Biological sciences 269: 1143–1151. doi: 10.1098/rspb.2002.1981 10.1098/rspb.2002.1981 PMC1691003 12061958

[B19] Denis JR (1923) Sur la faune française des Aptérygotes, IV. Note préliminaire. Bulletin de la Société Entomologique de France 92: 53–58.

[B20] Felderhoff KL Bernard EC Moulton JK (2010) Survey of *Pogonognathellus* Börner (Collembola: Tomoceridae) in the Southern Appalachians based on morphological and molecular data. Annals of the Entomological Society of America 103: 472–491. doi: 10.1603/AN09105

[B21] Folsom JW (1924) New species of Collembola From New York State. American Museum Novitates 108: 1–12.

[B22] Frati F Carapelli A Fanciulli PP (1995) The genus *Isotomurus*: where molecular markers help to evaluate the importance of morphological characters for the diagnosis of species. Polskie Pismo Entomologiczne 64: 41–51.

[B23] Frati F Dell’Ampio E Casasanta S Carapelli A Fanciulli PP (2000) Large amounts of genetic divergence among Italian species of the genus *Orchesella* (Insecta, collembola) and the relationships of two new species. Molecular Phylogenetics and Evolution 17: 456–461. doi: 10.1006/mpev.2000.0854 11133199 10.1006/mpev.2000.0854

[B24] Frati F Fanciulli PP Dallai R (1994) Further acquisitions on systematic relationships within the genus *Orchesella* (Collembola, Entomobryidae) using allozymes. Acta Zoologica Fennica 195: 35–43.

[B25] Furman DP Bukharina TA (2008) Genetic control of macrochaetae development in *Drosophila melanogaster*. Russian Journal of Developmental Biology 39: 195–206. doi: 10.1134/S1062360408040012 18792637

[B26] Guthrie JE (1903) The Collembola of Minnesota. (Vol. 4). Geological and Natural History Survey of Minnesota, Minneapolis, Minnesota, 110 pp. doi: 10.5962/bhl.title.1701

[B27] Heitzler P Haenlin M Ramain P Calleja M Simpson P (1996) A genetic analysis of pannier, a gene necessary for viability of dorsal tissues and bristle positioning in *Drosophila*. Genetics 143: 1271–1286. 8807299 10.1093/genetics/143.3.1271 PMC1207396

[B28] Jordana R (2012) Synopses of Palaearctic Collembola: Capbryinae & Entomobryini. Soil Organisms 84: 1–390.

[B29] Jordana R Baquero E (2005) A proposal of characters for taxonomic identification of *Entomobrya* species (Collembola, Entomobryomorpha), with description of a new species. Abhandlungen und Berichte des Naturkundemuseums Görlitz 76: 117–134.

[B30] Katz AD Giordano R Soto-Adames FN (2015) Operational criteria for cryptic species delimitation when evidence is limited, as exemplified by North American *Entomobrya* (Collembola: Entomobryidae). Zoological Journal of the Linnean Society 173: 810–840. doi: 10.1111/zoj.12220

[B31] Lee B-H Hwang U-W Kim W Park K-H Kim J-T (1995) Phylogenetic study of the suborder Arthropleona (Insecta: Collembola) based on morphological characters and 18S rDNA sequence analysis. Polskie Pismo Entomologiczne 64: 261–277.

[B32] Lee B-H Park K-H (1991) A systematic study of Korean Entomobryidae (Collembola, Insecta) based on cladistic analysis of phenotypic and allozyme data. The Korean Journal of Zoology 34: 265–288.

[B33] Lewis PO (2001) A likelihood approach to estimating phylogeny from discrete morphological character data. Systematic biology 50: 913–925. doi: 10.1080/106351501753462876 12116640 10.1080/106351501753462876

[B34] Leyns L Gómez-Skarmeta JL Dambly-Chaudière C (1996) Iroquois: A prepattern gene that controls the formation of bristles on the thorax of *Drosophila*. Mechanisms of Development 59: 63–72. doi: 10.1016/0925-4773(96)00577-1 8892233 10.1016/0925-4773(96)00577-1

[B35] Linnaeus C (1758) Systema Naturae, Ed. 10: 608–609.

[B36] Mari-Mutt JA (1979) A revision of the genus *Dicranocentrus* Schött (Insecta: Collembola: Entomobryidae). Bulletin of the University of Puerto Rico 259: 1–79.

[B37] Mari-Mutt JA (1986) Puerto Rican species of *Lepidocyrtus* and *Pseudosinella* (Collembola: Entomobryidae). Caribbean Journal of Science 22: 1–48.

[B38] Miller MA Pfeiffer W Schwartz T (2010) Creating the CIPRES Science Gateway for inference of large phylogenetic trees. In: 2010 Gateway Computing Environments Workshop (GCE), 1–8. doi: 10.1109/GCE.2010.5676129

[B39] Packard AS (1873) Synopsis of the Thysanura of Essex County, Mass., with descriptions of a few extralimital forms. Annual Report of the Trustees of the Peabody Academy of Science 5: 23–51.

[B40] Porco D Potapov M Bedos A Busmachiu G Weiner WM Hamra-Kroua S Deharveng L (2012) Cryptic diversity in the ubiquist species *Parisotoma notabilis* (Collembola, Isotomidae): a long-used chimeric species? PLoS ONE 7: . doi: 10.1371/journal.pone.0046056 10.1371/journal.pone.0046056 PMC3458803 23049931

[B41] Posada D (2008) jModelTest: phylogenetic model averaging. Molecular Biology and Evolution 25: 1253–1256. doi: 10.1093/molbev/msn083 18397919 10.1093/molbev/msn083

[B42] Potapov M Kremenitsa A (2008) Comments on the chaetotaxy of the genus *Orchesella* (Collembola, Entomobryomorpha) with a redefinition of the ‘*spectabilis*’ group and description of a new species of *Orchesella* from the Caucasus. Soil Organisms 80: 99–115.

[B43] Ramel G Baquero E Jordana R (2008) Biodiversity of the Collembola Fauna of Wetland Kerkini (N. Greece), with description of the sexual dimorphism of *Entomobrya atrocincta* Schött 1896 (Collembola: Entomobryomorpha). Annales de la Société Entomologique de France 44: 113–128. doi: 10.1080/00379271.2008.10697548

[B44] Rondani C (1861) Dipterlogiae Italicae Prodromus. 4:40. A. Stochi, Parma.

[B45] Ronquist F Teslenko M van der Mark P Ayres DL Darling A Höhna S Larget B Liu L Suchard MA Huelsenbeck JP (2012) MrBayes 3.2: efficient Bayesian phylogenetic inference and model choice across a large model space. Systematic Biology 61: 539–542. doi: 10.1093/sysbio/sys029 22357727 10.1093/sysbio/sys029 PMC3329765

[B46] Schäffer C (1896) Die Collembolen der Umgebung von Hamburg und benachbarter Gebiete. Mitteilungen aus dem Naturhistorischen Museum in Hamburg 13: 149–216.

[B47] Schött H (1896) North American Apterygogenea. Proceedings of the California Academy of Sciences, Series 2 6: 169–196.

[B48] Simonsen V Krogh PH Filser J Fjellberg A (1999) Three species of *Isotoma* (Collembola, Isotomidae) based on morphology, isozymes and ecology. Zoologica Scripta 28: 281–287. doi: 10.1046/j.1463-6409.1999.00025.x

[B49] Simpson P (1990) Lateral inhibition and the development of the sensory bristles of the adult peripheral nervous system of *Drosophila*. Development 109: 509–519. 2205467 10.1242/dev.109.3.509

[B50] Simpson P Woehl R Usui K (1999) The development and evolution of bristle patterns in Diptera. Development 126: 1349–1364. 10068629 10.1242/dev.126.7.1349

[B51] Soto-Adames FN (2002) Molecular phylogeny of the Puerto Rican *Lepidocyrtus* and *Pseudosinella* (Hexapoda: Collembola), a validation of Yoshii’s “color pattern species”. Molecular phylogenetics and evolution 25: 27–42. doi: 10.1016/S1055-7903(02)00250-6 12383748 10.1016/s1055-7903(02)00250-6

[B52] Soto-Adames FN (2008) Postembryonic development of the dorsal chaetotaxy in *Seira dowlingi* (Collembola, Entomobryidae); with an analysis of the diagnostic and phylogenetic significance of primary chaetotaxy in Seira. Zootaxa 1683: 1–31.

[B53] Soto-Adames FN (2010) Two new species and descriptive notes for five *Pseudosinella* species (Hexapoda: Collembola: Entomobryidae) from West Virginian (USA) Caves. Zootaxa 2331: 1–34.

[B54] Soto-Adames FN Barra J-A Christiansen K Jordana R (2008) Suprageneric classification of Collembola Entomobryomorpha. Annals of the Entomological Society of America 101: 501–513. doi: 10.1603/0013-8746(2008)101[501:SCOCE]2.0.CO;2

[B55] South A (1961) The taxonomy of the British species of *Entomobrya* (Collembola). Transactions of the Royal Entomological Society of London 113: 387–416. doi: 10.1111/j.1365-2311.1961.tb00798.x

[B56] Stach J (1963) The Apterygotan Fauna of Poland in relation to the World-fauna of this group of Insects: Tribe: Entomobryini. Acta monographica Musei Historiae naturalis, Polska Akademia Nauk. Inst. Zool. Karakow, 125 pp.

[B57] Szeptycki A (1979) Chaetotaxy of the Entomobryidae and its phylogenetical significance. Morpho-systematic studies on Collembola (Vol. 4). Państwowe Wydawnictwo Naukowe, Warsaw, 1–218.

[B58] Tullberg T (1871) Forteckning ofver Svenska Podurider. Ofversigt af Kongliga Vetenskaps-akademiens forhandlingar 28: 143–145.

[B59] Will KW Rubinoff D (2004) Myth of the molecule: DNA barcodes for species cannot replace morphology for identification and classification. Cladistics 20: 47–55. doi: 10.1111/j.1096-0031.2003.00008.x 10.1111/j.1096-0031.2003.00008.x 34892971

[B60] Xiong Y Gao Y Yin W Luan Y (2008) Molecular phylogeny of Collembola inferred from ribosomal RNA genes. Molecular Phylogenetics and Evolution 49: 728–735. doi: 10.1016/j.ympev.2008.09.007 18835455 10.1016/j.ympev.2008.09.007

[B61] Zhang F Bedos A Deharveng L (2014a) Disjunct distribution of *Szeptyckiella* gen. n. from New Caledonia and South China undermines the monophyly of Willowsiini (Collembola: Entomobryidae). Journal of Natural History 48: 1299–1317. doi: 10.1080/00222933.2013.859317.

[B62] Zhang F Chen Z Dong RR Deharveng L Stevens MI Huang YH Zhu CD (2014b) Molecular phylogeny reveals independent origins of body scales in Entomobryidae (Hexapoda: Collembola). Molecular Phylogenetics and Evolution 70: 231–239. doi: 10.1016/j.ympev.2013.09.024 24099889 10.1016/j.ympev.2013.09.024

[B63] Zhang F Deharveng L (2015) Systematic revision of Entomobryidae (Collembola) by integrating molecular and new morphological evidence. Zoologica Scripta 44: 298–311. doi: 10.1111/zsc.12100

[B64] Zhang F Palacios-Vargas JG Chen J-X (2007) The genus *Willowsia* and its Mexican species (Collembola: Entomobryidae). Annals of the Entomological Society of America 100: 36–40. doi: 10.1603/0013-8746(2007)100[36:TGWAIM]2.0.CO;2

[B65] Zhang F Yu D Luo Y Ho SYW Wang B Zhu C (2014c) Cryptic diversity, diversification and vicariance in two species complexes of *Tomocerus* (Collembola, Tomoceridae) from China. Zoologica Scripta 43: 393–404.

[B66] Zhang F Yu D Xu G (2011) Transformational homology of the tergal setae during postembryonic development in the *Sinella*-*Coecobrya* group (Collembola: Entomobryidae). Contributions to Zoology 80: 213–230.

