# Supplementary material for: Taxonomic review and phylogenetic analysis of fifteen North American Entomobrya (Collembola, Entomobryidae), including four new species
Source: Zookeys. 2015 Oct 5;(525):1–75. doi: 10.3897/zookeys.525.6020 (PMC4607850; doi:10.3897/zookeys.525.6020)
Supplement: Supplementary material 2 — Distribution maps [file zookeys-525-001-s002.pdf]

Supplementary material 2. Distribution maps: A, Entomobrya assuta; B, *Entomobrya atrocincta*; C, *Entomobrya bicolor*; D, *Entomobrya citrensis* sp. n.; E, *Entomobrya clitellaria*; F, *Entomobrya decemfasciata*; G, *Entomobrya intermedia*; H, *Entomobrya jubata* sp. n.; I, *Entomobrya ligata*; J, *Entomobrya multifasciata*; K, *Entomobrya neotenica* sp. n.; L, *Entomobrya nivalis*; M, *Entomobrya quadrilineata*; N, *Entomobrya unifasciata* sp. n.; O, *Entomobrya unostrigata*.

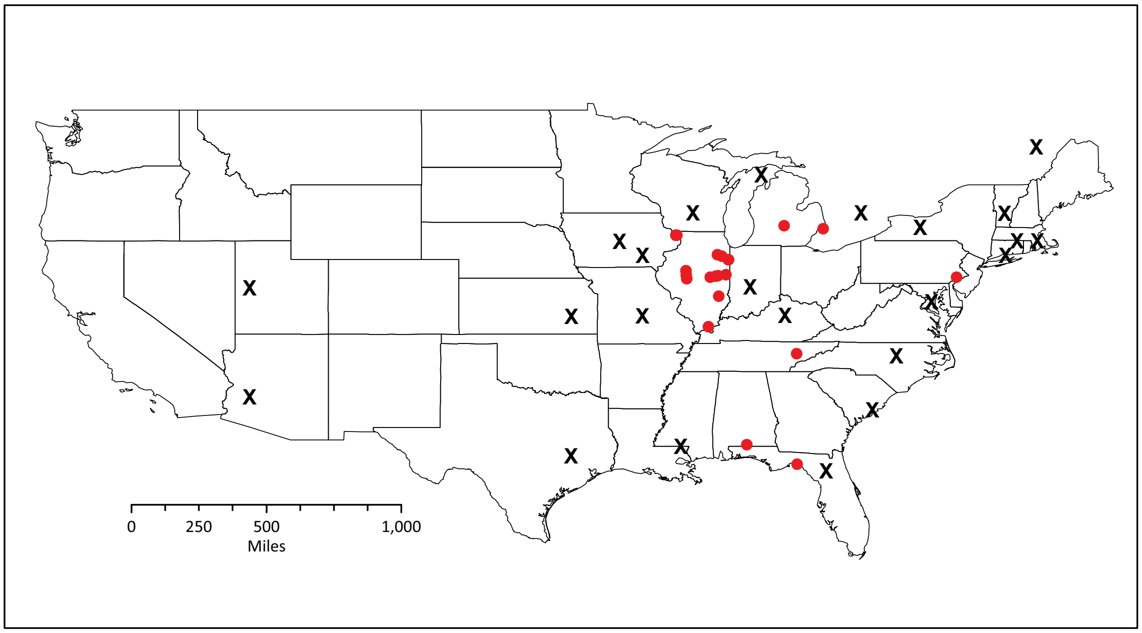

A. Distribution map for *Entomobrya assuta*. Sample localities for specimens examined and collected for this study are marked as red circles. Additional reported localities (Christiansen & Bellinger, 1998) are labeled X.

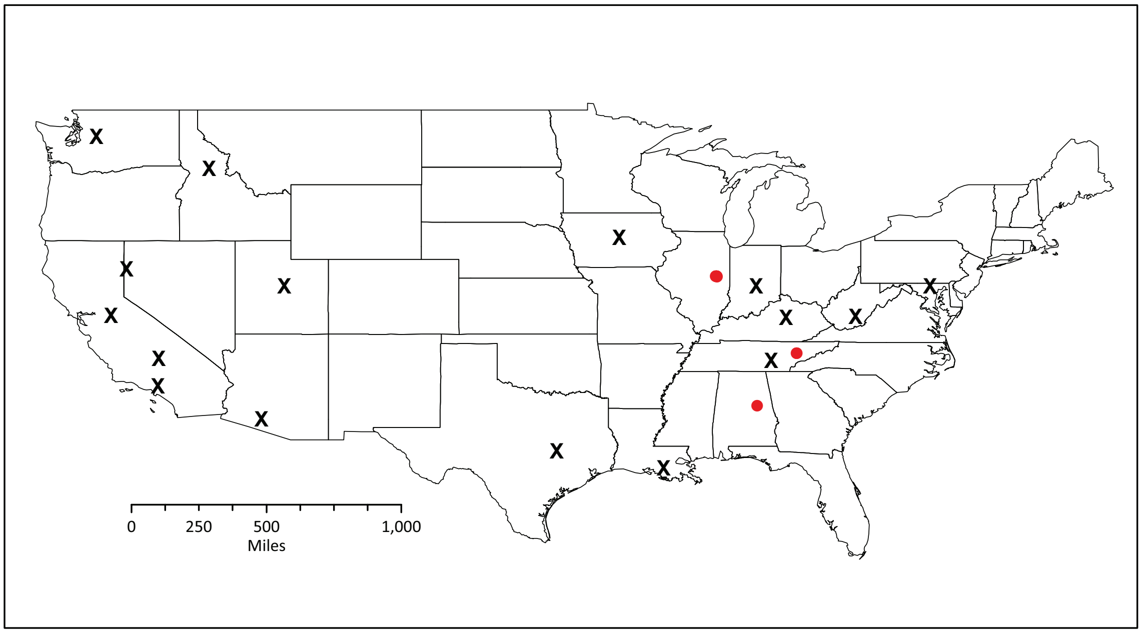

B. Distribution map for *Entomobrya atrocincta*. Sample localities for specimens examined and collected for this study are marked as red circles. Additional reported localities (Christiansen & Bellinger 1998) are labeled X.

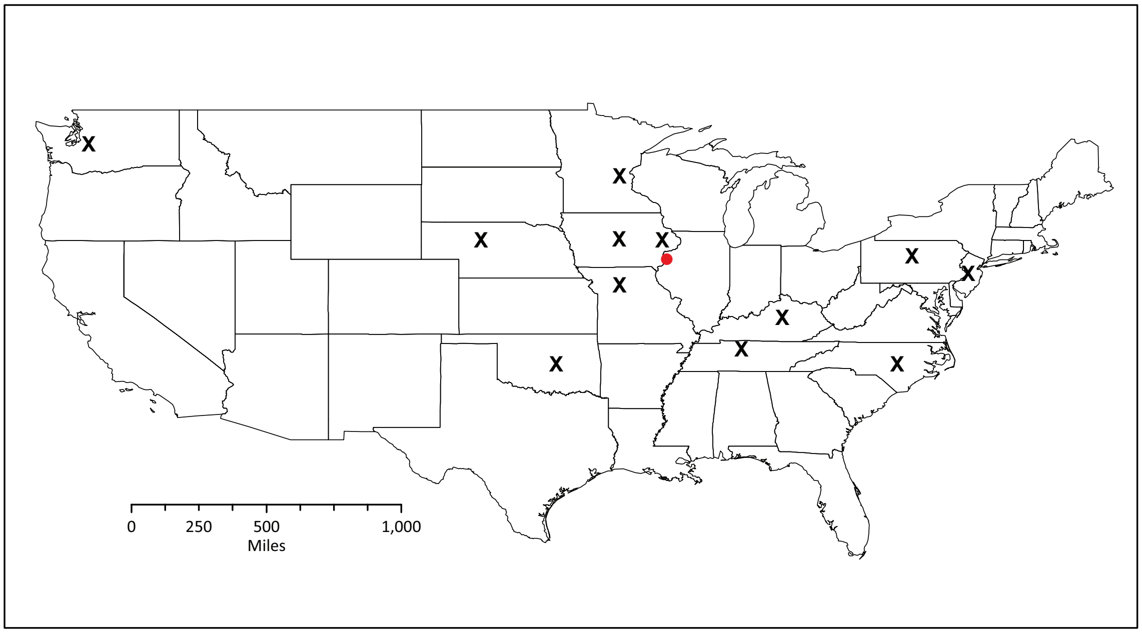

C. Distribution map for *Entomobrya bicolor*. The sample locality for specimens examined in this study is marked as a red circle. Additional reported localities (Christiansen & Bellinger 1998) are labeled X.

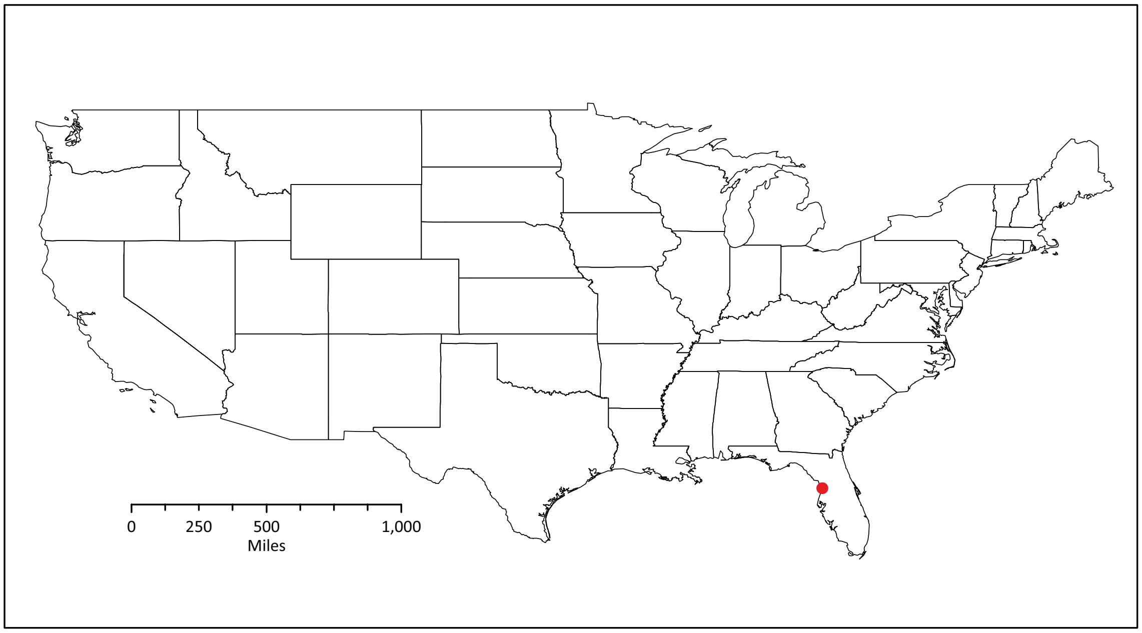

D. Distribution map for *Entomobrya citrensis* sp. n. The sample locality for specimens examined in this study is marked as a red circle.

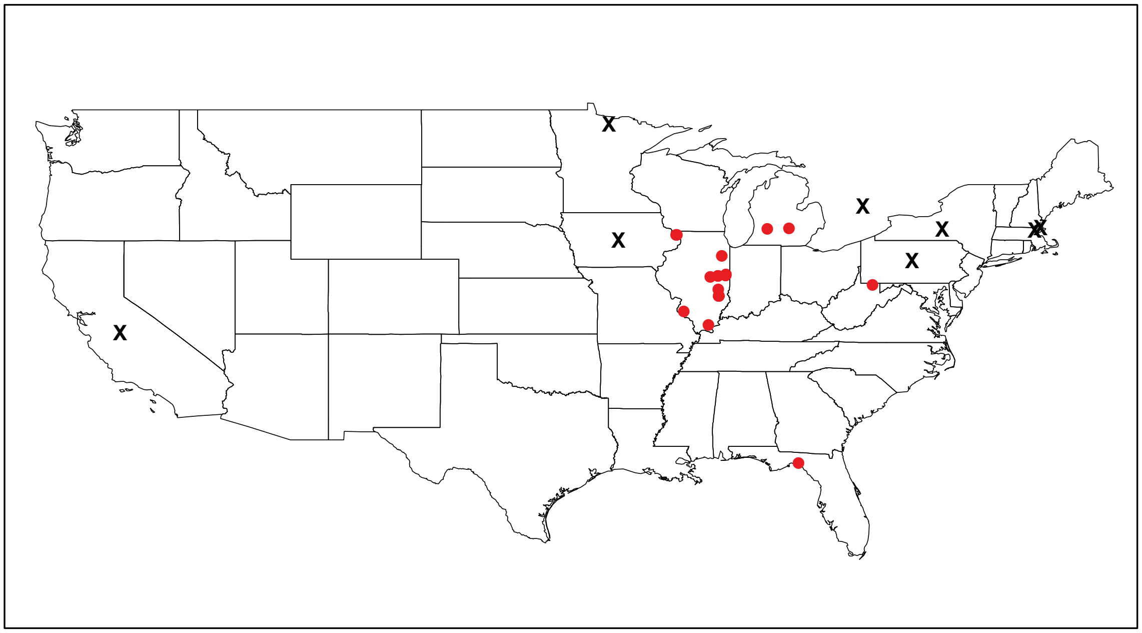

E. Distribution map for *Entomobrya clitellaria*. Sample localities for specimens examined and collected for this study are marked as red circles. Additional reported localities (Christiansen & Bellinger 1998) are labeled X.

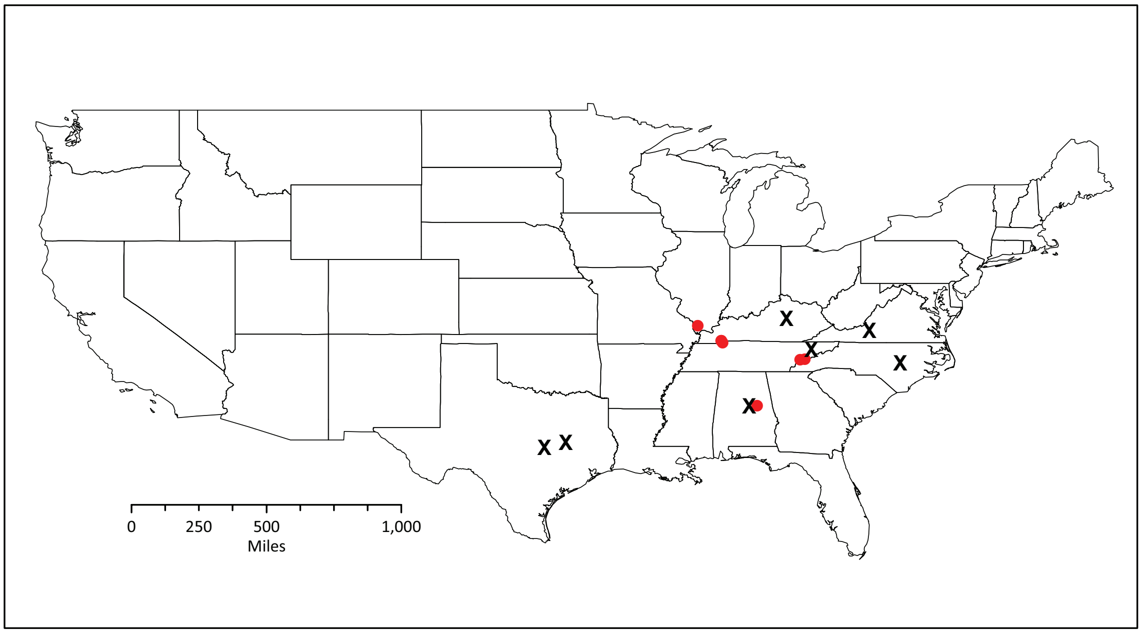

F. Distribution map for *Entomobrya decemfasciata*. Sample localities for specimens examined and collected for this study are marked as red circles. Additional reported localities (Christiansen & Bellinger 1998) are labeled X.

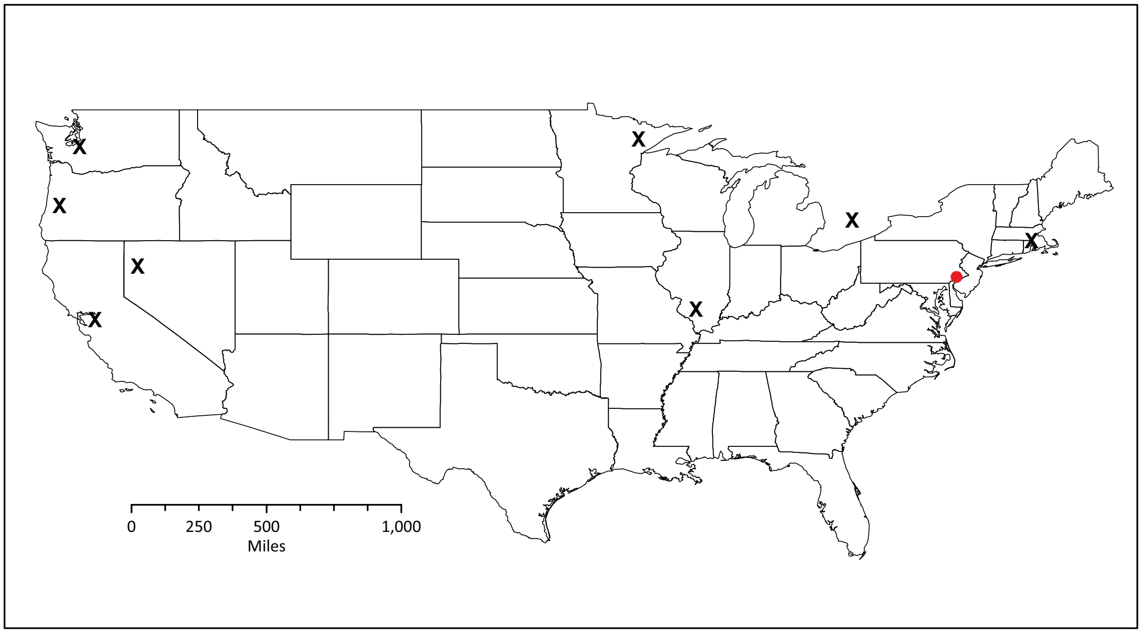

G. Distribution map for *Entomobrya intermedia*. The sample locality for specimens examined and collected for this study is marked as a red circle. Additional reported localities (Christiansen & Bellinger 1998) are labeled X.

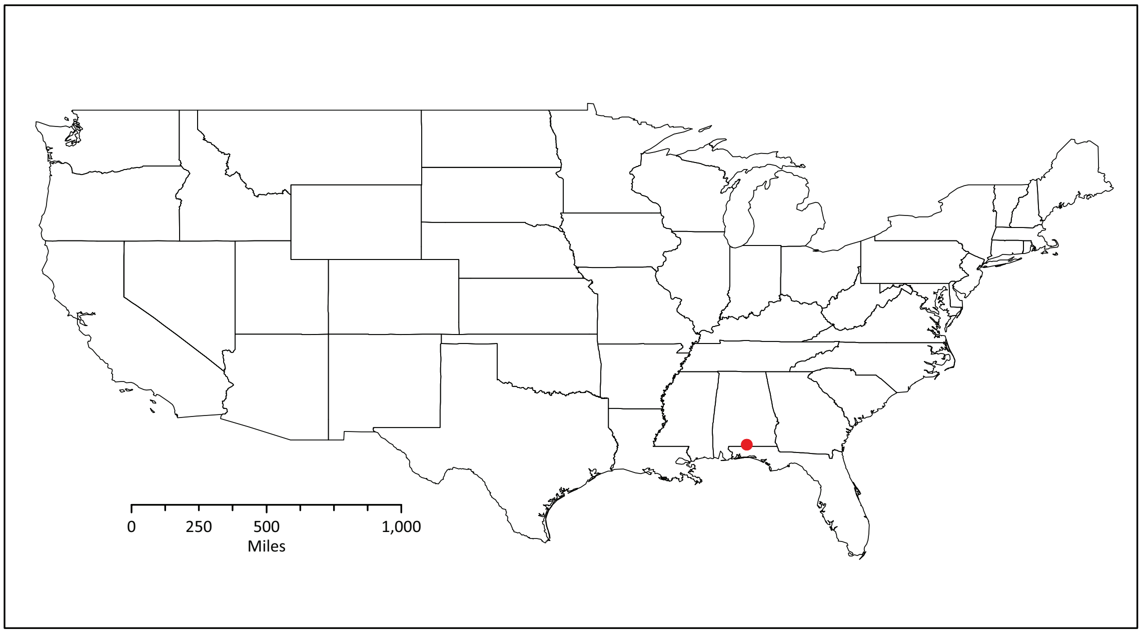

H. Distribution map for *Entomobrya jubata* sp. n. The sample locality for specimens examined in this study is marked as a red circle.

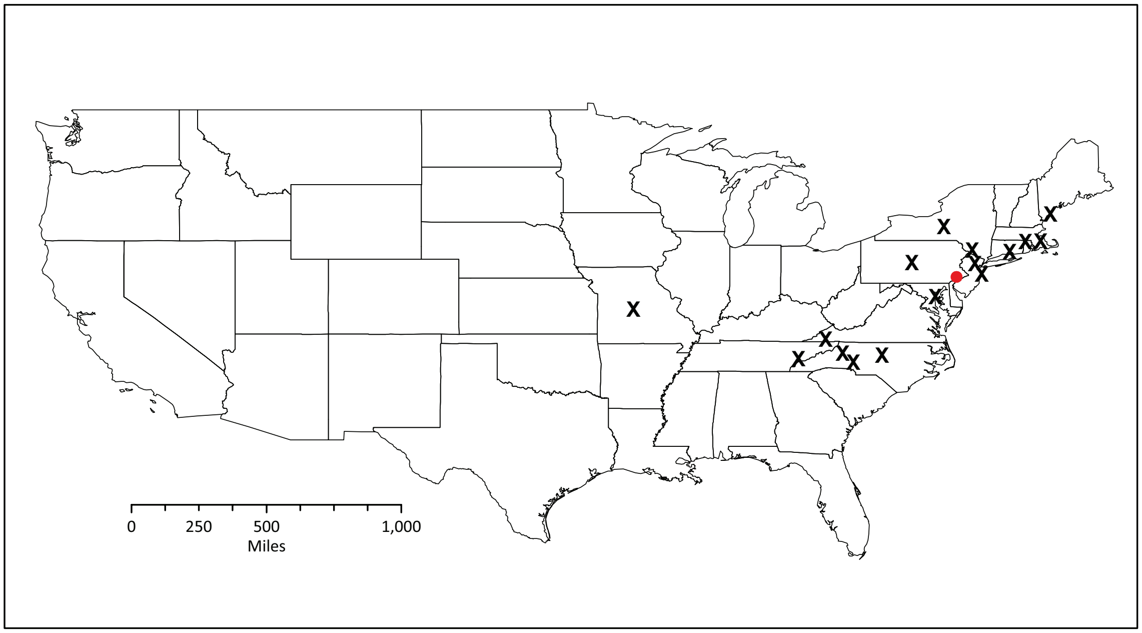

I. Distribution map for *Entomobrya ligata*. The sample locality for specimens examined and collected for this study is marked as a red circle. Additional reported localities (Christiansen & Bellinger 1998) are labeled X. Much of this range may represent *E. unifasciata* sp. n., especially records from the Midwest.

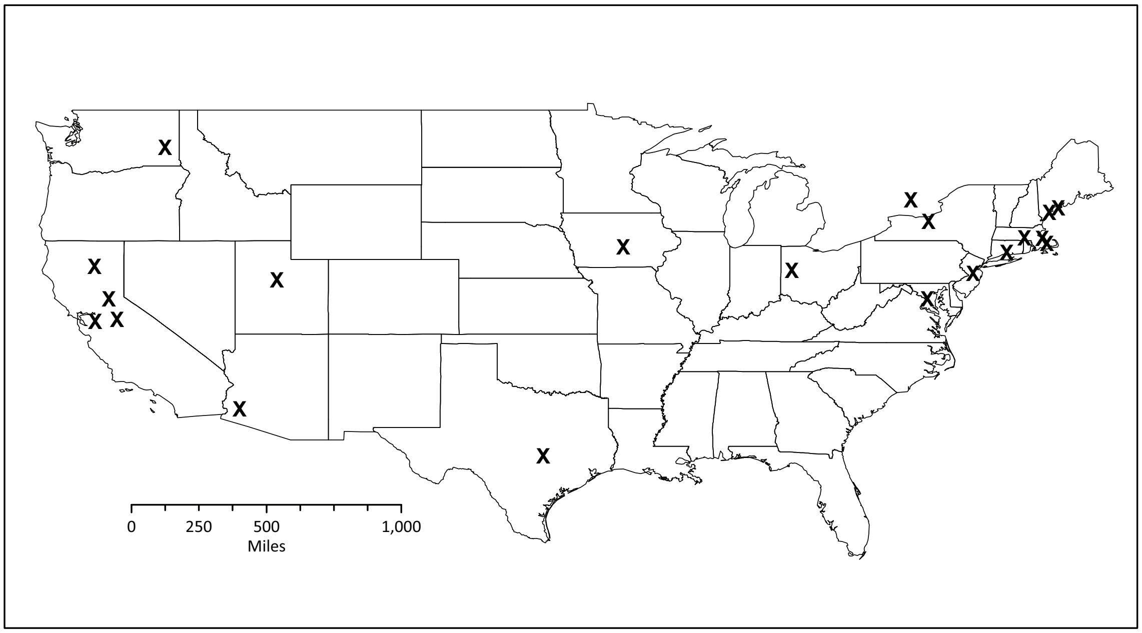

J. Distribution map for *Entomobrya multifasciata*. Reported localities (Christiansen & Bellinger 1998) are labeled X. Note these localities are highly suspect. Specimens may have been misidentified considering their color pattern and chaetotaxy is very similarity to *E. atrocincta* females. All *E. multifasciata s*pecimens examined in this study were collected from São Miguel Island, Azores, Portugal.

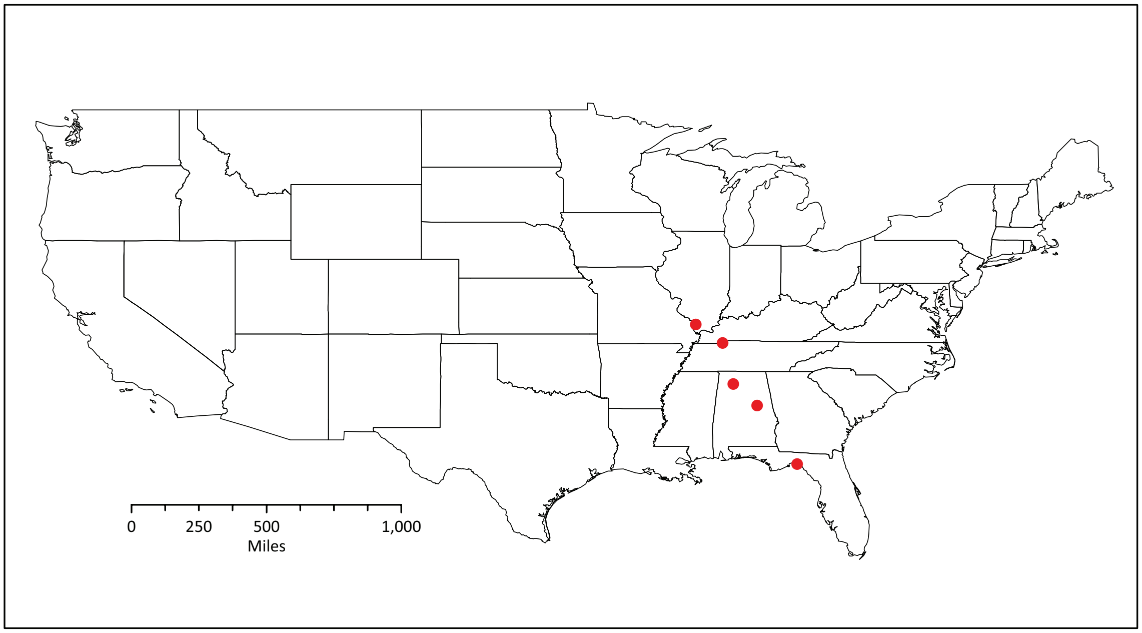

K. Distribution map for *Entomobrya neotenica* sp. n. Sample localities for specimens examined and collected for this study are marked as red circles.

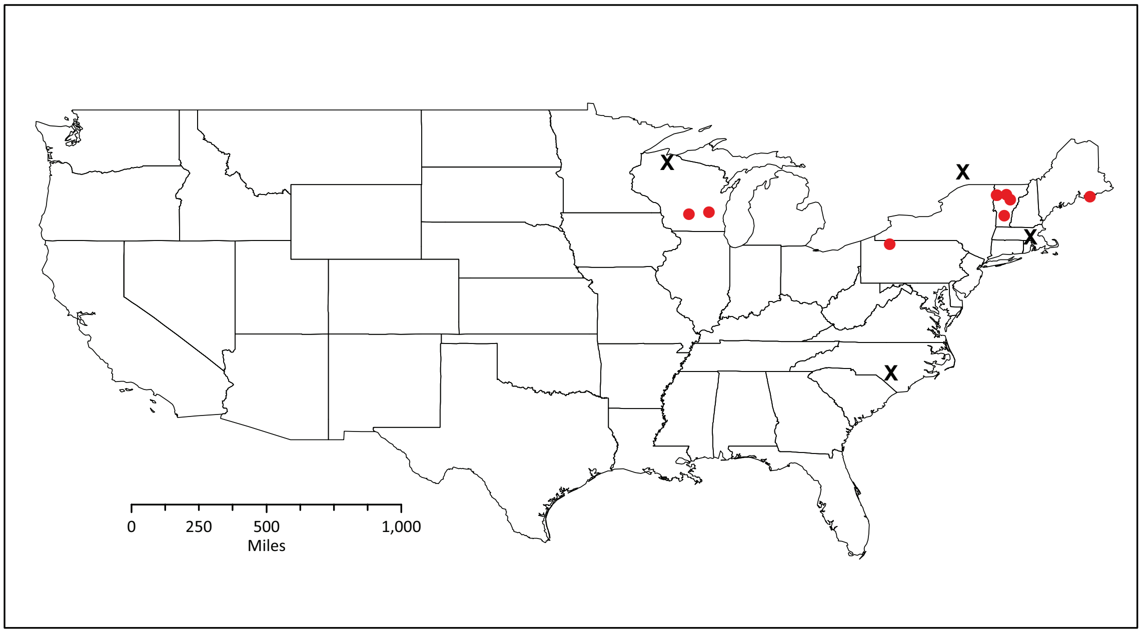

L. Distribution map for *Entomobrya nivalis*. Sample localities for specimens examined and collected for this study are marked as red circles. Additional reported localities (Christiansen & Bellinger 1998) are labeled X.

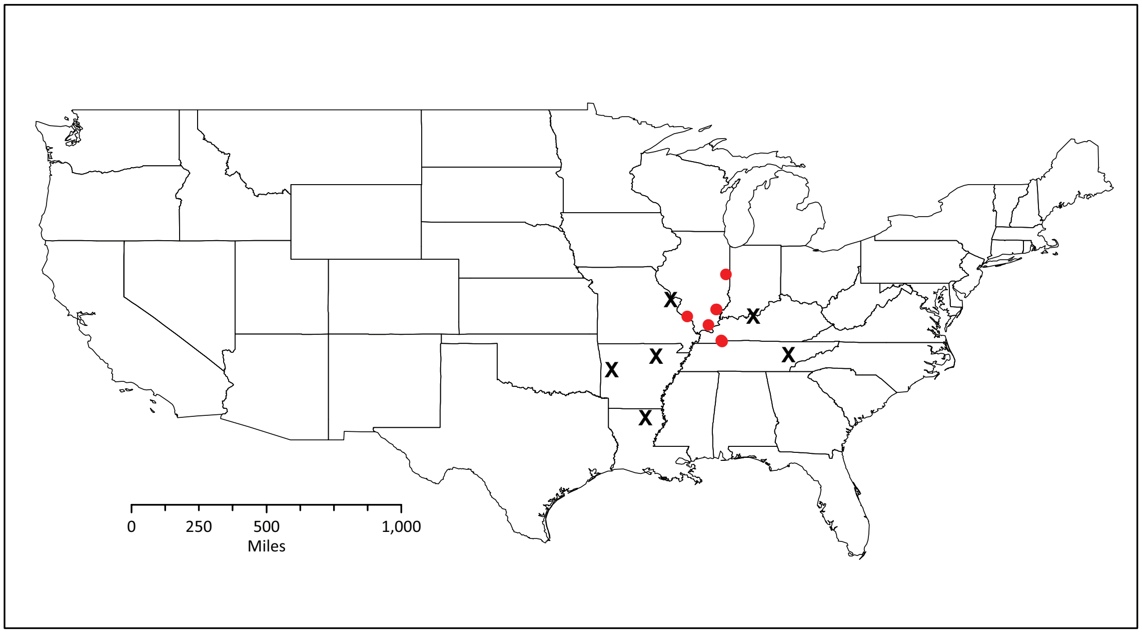

M. Distribution map for *Entomobrya quadrilineata*. Sample localities for specimens examined and collected for this study are marked as red circles. Additional reported localities (Christiansen & Bellinger, 1998) are labeled X.

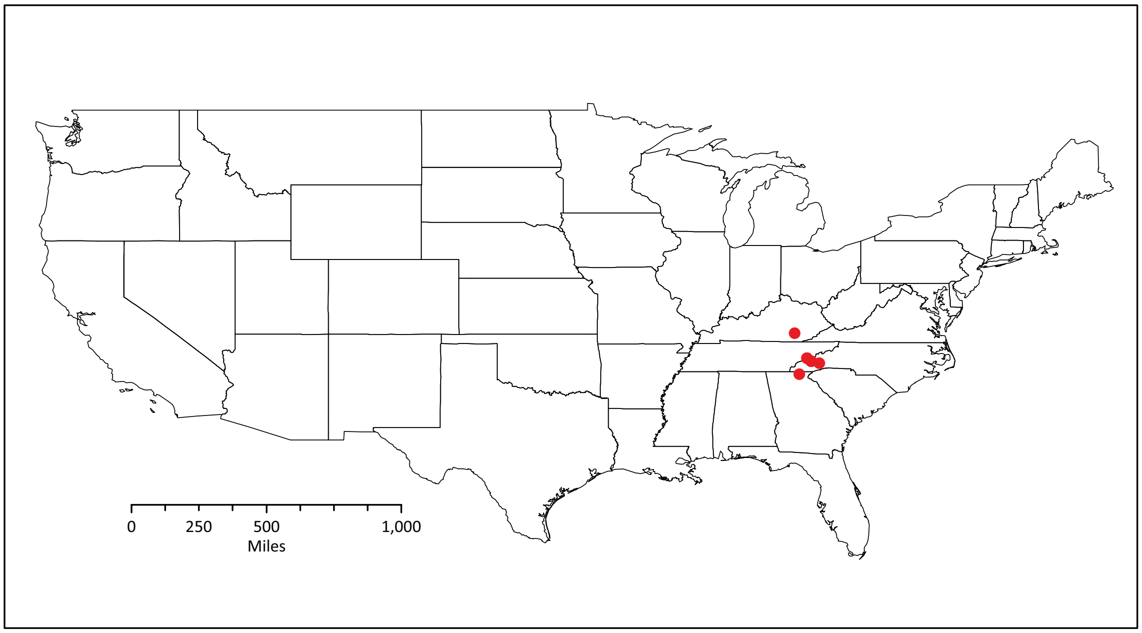

N. Distribution map for *Entomobrya unifasciata* sp. n. Sample localities for specimens examined and collected for this study are marked as red circles.

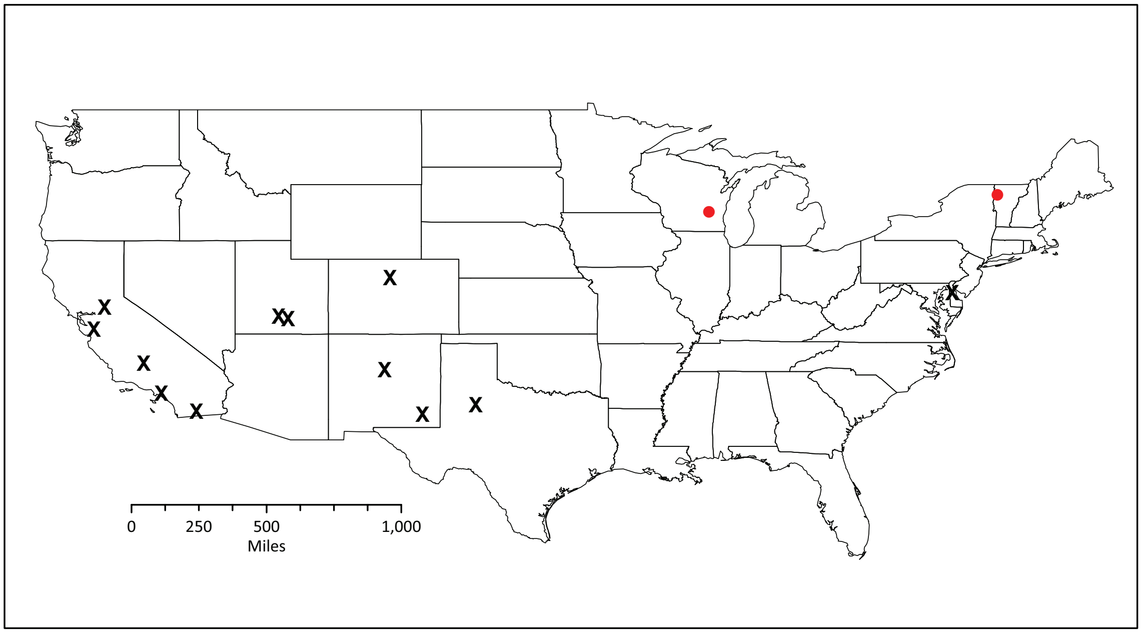

O. Distribution map for *Entomobrya unostrigata*. Sample localities for specimens examined and collected for this study are marked as red circles. Additional reported localities (Christiansen & Bellinger, 1998) labeled X.
