# Supplementary material for: Taxonomic review and phylogenetic analysis of fifteen North American Entomobrya (Collembola, Entomobryidae), including four new species
Source: Zookeys. 2015 Oct 5;(525):1–75. doi: 10.3897/zookeys.525.6020 (PMC4607850; doi:10.3897/zookeys.525.6020)

Supplementary material 3. Photographs of type specimens stored at the Illinois Natural History Survey, Champaign, IL. Lateral (A) and dorsal (B) views of a neo-paratype specimen of *Entomobrya quadrilineata* Bueker, 1939, Fountain Bluff, IL., V.15.1932, Ross & Mohr. Lateral view of a co- type specimen of *Entomobrya decemfasciata* Packard 1873 (C), no locality information. *Entomobrya ligata* Folsom 1924: Co-type specimens, Roorheesville?, NY 30Aug1923, M. J. Leonard (D,E,F); Co-type specimen, NY, July 1923, A. Wolf (G).

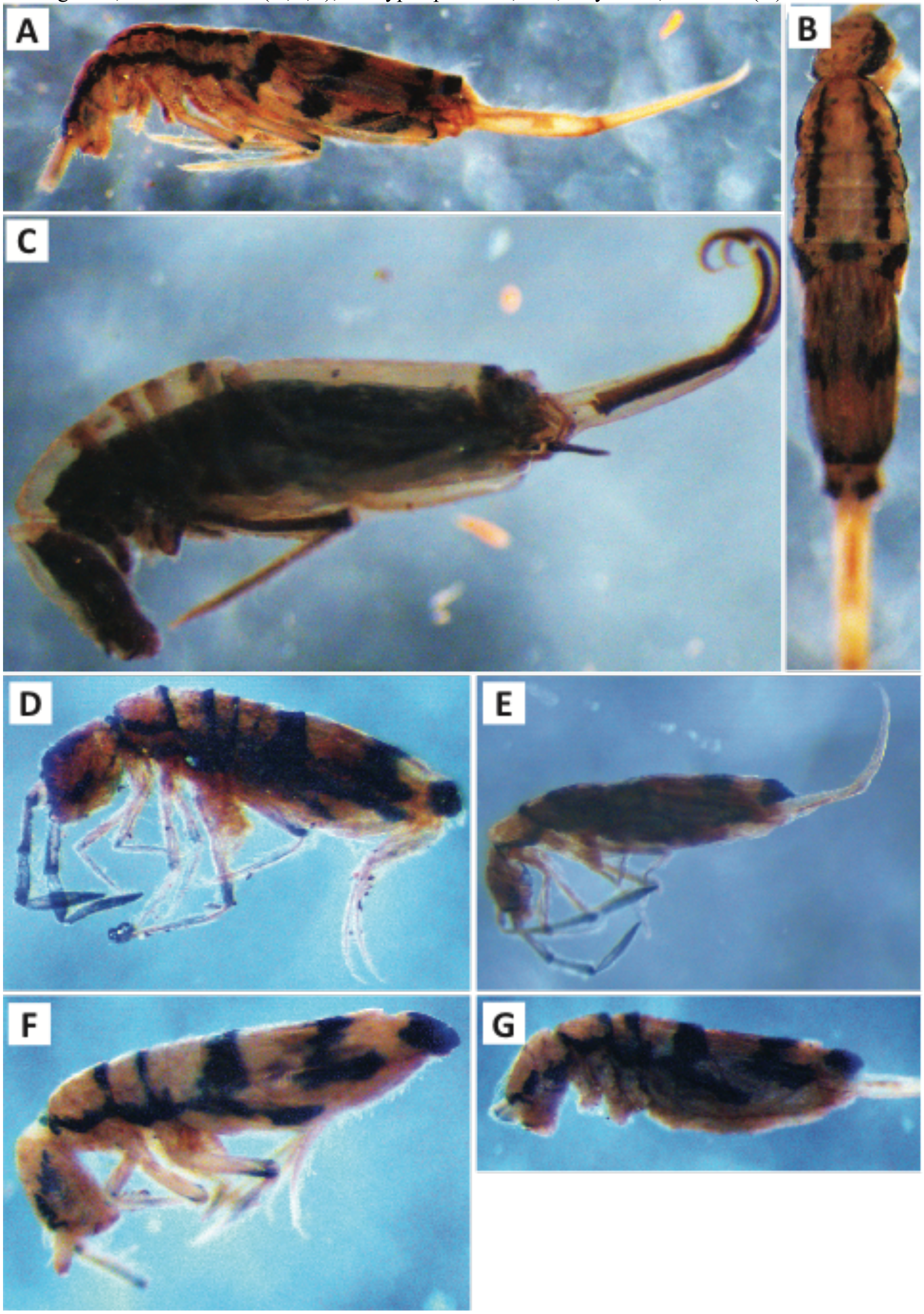

Supplement: Supplementary material 3 — Photographs of type specimens [file zookeys-525-001-s003.pdf]
